# The Effects of Mindfulness‐Based Programs on Pain‐Related Cognitions in Individuals With Chronic Musculoskeletal Pain: A Systematic Review and Meta‐Analysis

**DOI:** 10.1155/prm/6495015

**Published:** 2026-11-09

**Authors:** Talia Barnet-Hepples, Fereshteh Pourkazemi, Evangelos Pappas, Linting Ruan, James Puterflam, Jemma Masterton, Anita Amorim

**Affiliations:** ^1^ Sydney School of Health Sciences Faculty of Medicine and Health The University of Sydney Sydney Australia sydney.edu.au; ^2^ School of Health and Biomedical Sciences Royal Melbourne Institute of Technology University Melbourne Australia rmit.edu.au; ^3^ Shanghai University of Medicine and Health Sciences Shanghai China sumhs.edu.cn; ^4^ Sydney School of Medical Sciences Faculty of Medicine and Health The University of Sydney Sydney Australia sydney.edu.au; ^5^ University Centre for Rural Health Faculty of Medicine and Health The University of Sydney Lismore Australia sydney.edu.au

**Keywords:** chronic musculoskeletal pain, meta-analysis, mindfulness-based programs, pain-related cognitions, systematic review

## Abstract

**Objectives:**

Chronic musculoskeletal pain is a complex condition influenced by psychological factors, including pain‐related cognitions. This systematic review and meta‐analysis of randomized controlled trials aimed to evaluate the effects of mindfulness‐based programs on pain‐related cognitions in individuals with chronic musculoskeletal pain. Secondary outcomes included depression, anxiety, psychological distress, pain intensity, disability, intervention adherence, and mindfulness‐related adverse events.

**Methods:**

Eight databases were searched from inception to January 2026. Evidence was synthesized using random‐effects models and reported as standardized mean differences with 95% confidence intervals. Risk of bias was assessed using the revised Cochrane risk of bias tool. Evidence certainty was rated using GRADE.

**Results:**

Fifty‐one studies (*n* = 5997) were included and had a moderate‐to‐high risk of bias. Compared to no intervention, mindfulness‐based programs reduced pain catastrophizing (SMD: 1.00, 95% CI: 0.56–1.45) and increased pain acceptance (SMD: 1.12, 95% CI: 0.31–1.92) post‐intervention. They also reduced depression (SMD: 0.94, 95% CI: 0.50–1.38), anxiety (SMD: 1.08, 95% CI: 0.57–1.59), psychological distress (SMD: 0.75, 95% CI: 0.12–1.38), pain intensity (SMD: 0.80, 95% CI: 0.38–1.23), and disability (SMD: 0.48, 95% CI: 0.04–0.92) compared to no intervention. Minimal significant differences were observed between the effect of mindfulness‐based programs and cognitive behavioral therapy on these outcomes.

**Discussion:**

Mindfulness‐based programs appear to be an effective self‐management strategy for individuals with chronic musculoskeletal pain. However, the certainty of evidence is mostly low with intervention adherence varying. Future research should clarify the direction and nature of the relationship between adherence and outcomes. Reporting of mindfulness‐related adverse events was insufficient to evaluate safety; future trials should incorporate comprehensive measurement.

## 1. Introduction

Chronic musculoskeletal pain can profoundly impact psychological well‐being, increasing the risk of mental health disorders such as depression and anxiety [1–6]. In turn, psychological factors such as stress [2, 7, 8], depression [9, 10], and anxiety [11] can contribute to the onset and persistence of chronic musculoskeletal pain. This affective distress is associated with increased pain intensity and worse physical function [12, 13].

There is also substantial evidence that other psychological factors, particularly pain‐related cognitions, contribute to the trajectory of chronic musculoskeletal pain. Pain‐related cognitions are the thoughts, beliefs, and attitudes individuals hold regarding their pain experience. Maladaptive pain‐related cognitions such as pain catastrophizing and kinesiophobia can exacerbate pain and disability in people with chronic musculoskeletal pain [14–18]. Conversely, positive cognitions such as pain acceptance and pain self‐efficacy are associated with lower pain intensity and better physical function [19–21]. Further, several studies suggest that among people with comorbid chronic musculoskeletal pain and depression or anxiety, pain‐related cognitions can mediate the bidirectional relationship between these conditions [22–24]. Addressing psychological factors within chronic musculoskeletal pain management is crucial [25–29].

Cognitive behavioral therapy (CBT) is widely considered the gold‐standard psychological treatment for depression and anxiety [30, 31] and is the main psychological intervention recommended in chronic musculoskeletal pain management guidelines [25, 26]. However, barriers to widespread implementation persist. CBT is typically delivered by mental health professionals, such as psychologists and psychiatrists, yet growing workforce shortages and rising demand have limited service availability [32–34]. Moreover, the conventional one‐on‐one format of CBT is resource‐intensive and more costly than group‐based approaches [35]. Additional scalable, accessible, and cost‐effective options are needed.

Mindfulness‐based programs have emerged as one potential alternative for addressing psychological well‐being in individuals with chronic musculoskeletal pain [36, 37]. Practicing mindfulness involves paying attention, on purpose, as nonjudgmentally and open‐heartedly as possible, to the present moment—to what one is experiencing in their body, their mind, and the world around them [38]. Mindfulness‐based programs involve sustained, intensive training in mindfulness meditation, complemented by inquiry—a reflective practice that employs questioning to examine one’s experiences with curiosity and nonjudgment and distinguish direct experiences from habitual reactions and conceptions. They are thought to reduce the stress associated with living with a chronic condition by fostering decentering and equanimity—defined as even‐mindedness in the face of challenging experiences—and by enhancing emotional and behavioral self‐regulation [39]. Various mindfulness‐based programs are described in the literature; the two most widely adopted are mindfulness‐based stress reduction (MBSR) and mindfulness‐based cognitive therapy (MBCT) [40].

Hundreds of studies have investigated the health impacts of mindfulness across various populations [36, 40, 41]. Among people with chronic musculoskeletal pain, they have been shown in randomized controlled trials (RCTs) to positively influence pain‐related cognitions [42–44], reduce symptoms of depression and anxiety [45], decrease perceptions of pain severity, and improve physical function [46]. However, findings across studies have been inconsistent.

The rapid growth of research and inconsistent findings in this area present challenges for clinicians and people living with chronic musculoskeletal pain by contributing to uncertainty in decision‐making and creating barriers to evidence‐based practice. While several reviews have endeavored to synthesize the evidence for the impact of mindfulness among people with chronic pain, important limitations remain.

First, no reviews have specifically focused on the effect of mindfulness‐based programs on pain‐related cognitions, despite their known relevance to clinical outcomes. Additionally, limited attention has been given to intervention adherence and mindfulness‐related adverse events (MRAEs). Second, many reviews examining important outcomes—including psychological well‐being, pain intensity, and disability—have been narrow in scope, focusing on a single pain condition such as chronic back pain or rheumatoid arthritis [47–52], or overly broad, including interventions with minimal mindfulness content such as acceptance and commitment therapy [53–55]. Third, as advised in the Cochrane Handbook for Systematic Reviews of Interventions [56], the inclusion of nonrandomized studies in several reviews increases the risk of bias, potentially leading to inaccurate conclusions [50, 54, 57, 58]. Finally, many meta‐analyses have pooled heterogeneous comparators, for instance, passive and active controls [51, 59–61] or diverse active controls [58, 59, 62], limiting the precision and interpretability of the findings. A rigorous synthesis of evidence addressing these limitations is needed to clarify the effects of mindfulness‐based programs in people with chronic musculoskeletal pain and to inform clinical practice.

Therefore, this systematic review and meta‐analysis of RCTs aimed to evaluate the effects of mindfulness‐based programs on pain‐related cognitions among people with chronic musculoskeletal pain. Secondary outcomes included depression, anxiety, psychological distress, pain intensity, disability, intervention adherence, and MRAEs.

## 2. Methods

This systematic review is reported in accordance with the Preferred Reporting Items for Systematic Reviews and Meta‐Analyses (PRISMA) guidelines [63]. The protocol was registered prospectively with PROSPERO (CRD42023495026); no deviations occurred. There was no patient or public involvement in this review.

### 2.1. Search Strategy

Eight databases were searched from inception to January 9, 2026: Medline (Ovid), Embase (Ovid), Cochrane Central Register of Controlled Trials (CENTRAL) (Ovid), Cumulative Index of Nursing and Allied Health Literature (CINAHL) (EBSCO), APA PsycINFO (Ovid), Allied and Complementary Medicine Database (AMED) (Ovid), Web of Science, and Scopus. Additionally, three clinical trial registries were searched from inception to January 9, 2026: the United States National Institutes of Health Ongoing Trials Register (ClinicalTrials.gov), the World Health Organization International Clinical Trials Registry Platform, and the Australian New Zealand Clinical Trials Registry. Search strategies incorporated a combination of keywords and Medical Subject Headings related to mindfulness and chronic musculoskeletal pain (Supporting Digital Content 1). The search strategies were reviewed by an experienced librarian and validated by confirming their ability to retrieve two known eligible studies [46, 64]. Reference lists of relevant studies were also hand searched by TBH.

Titles and abstracts of retrieved studies were screened by two of six independent reviewers using predefined eligibility criteria (TBH, FP, EP, LR, JP, and AA). Full‐text articles of potentially eligible studies were subsequently assessed by two of four independent reviewers (TBH, FP, LR, and AA). Disagreements regarding eligibility at either stage were resolved through discussion to reach consensus or arbitration by a third reviewer not involved in the initial decision. Authors of identified studies were contacted via email if additional information was required.

### 2.2. Eligibility Criteria

#### 2.2.1. Study Design

Only published RCTs were included. No restrictions were placed on language or publication date.

#### 2.2.2. Participants

Eligible trials were those involving participants of any age reporting chronic primary or secondary musculoskeletal pain, as defined by the International Classification of Diseases, 11th revision [65]. Trials involving participants with chronic back pain were included when all participants had back pain classified as chronic primary or secondary musculoskeletal pain or when a subset of participants may have had radicular pain. Trials that exclusively included participants with radicular pain as well as trials involving participants with other forms of chronic neuropathic pain were excluded. Trials involving participants with chronic cancer‐related pain, chronic postsurgical pain, chronic headache (including tension‐type headache), or chronic visceral pain were excluded.

#### 2.2.3. Interventions

Trials were included if the intervention was aligned with Crane et al.’s [39] definition of a mindfulness‐based program as an intervention that

a. is informed by theories and practices that draw from a confluence of contemplative traditions, science, and the major disciplines of medicine, psychology, and education;
b. is underpinned by a model of human experience which addresses the causes of human distress and the pathways to relieving it;
c. develops a new relationship with experience characterized by present‐moment focus, decentering, and an approach orientation;
d. supports the development of qualities such as joy, compassion, wisdom, equanimity, and greater attentional, emotional, and behavioral self‐regulation; and
e. engages participants in sustained intensive training in mindfulness meditation practice, in an experiential inquiry‐based learning process, and in exercises to develop understanding [39].

Examples include MBSR and MBCT. Adaptations of these programs were eligible if an emphasis on the practice of mindfulness meditation remained the central pedagogical component. Interventions that incorporated practices informed by mindfulness but did not have training in mindfulness meditation as the primary focus, such as acceptance and commitment therapy, dialectical behavioral therapy, or compassion‐focused therapy, were excluded.

#### 2.2.4. Comparators

No restrictions were placed on the type of comparator.

#### 2.2.5. Outcome Measures

Trials were required to measure at least one of the following: pain‐related cognitions (i.e., pain catastrophizing, pain self‐efficacy, chronic pain acceptance, kinesiophobia, or pain control), depression, anxiety, psychological distress, pain intensity, disability, intervention adherence, or MRAEs.

### 2.3. Data Extraction

Data were independently extracted by two of three reviewers (TBH and LR or JM) and verified by a further reviewer (AA). Discrepancies were resolved by consensus. Extracted information included study design, participant characteristics, comparator characteristics, funding sources, and conflicts of interest. Intervention characteristics were extracted according to the Template for Intervention Description and Replication (TIDieR) framework [66]. Outcome data collected immediately following intervention completion were extracted for analysis of post‐intervention effects. If data were also available from a time point ≥ 12 months post‐randomization, these scores were used to analyze long‐term effects. If multiple eligible outcome measures were available for an outcome domain, the measure more commonly used across the included trials was extracted. Where this guideline could not be applied, the most appropriate measure was determined through consensus among the reviewers.

### 2.4. Data Analysis

Clinical heterogeneity was assessed through structured comparison of key study characteristics, including participant characteristics, intervention and comparator characteristics, outcome measures, and assessment time points. This assessment was used to inform decisions about whether studies were sufficiently similar to combine in meta‐analyses, whether subgroup or sensitivity analyses were appropriate and feasible, and how findings should be interpreted. Disagreements regarding the implications of clinical heterogeneity for evidence synthesis were discussed among reviewers until consensus was reached.

Meta‐analyses were conducted only for outcomes and comparisons for which at least four eligible studies were available, in line with current standards in the field [67]. Random‐effects models were used to account for anticipated heterogeneity. All meta‐analyses included studies that used varied outcome measures. As such, treatment effects were expressed as standardized mean differences (SMDs; Hedges’ g) with 95% confidence intervals (CIs). SMDs were calculated using the post‐intervention mean and standard deviation (SD) for each group. Effect sizes were categorized as small (0.2–0.4), medium (0.5–0.7), or large (≥ 0.8) [68]. Effect directions were coded so that positive SMDs indicated effects favoring mindfulness‐based programs for all outcomes. Study authors were contacted via email regarding missing data. If no response was received, the following strategies were employed. Results reported as post‐intervention medians and ranges were converted to means and SDs using Cochrane Handbook guidelines [69]. Standard errors or 95% CIs were used to calculate missing SDs, following Cochrane Handbook recommendations [69]. If algebraic recalculation was not feasible, SDs were imputed based on the mean of the observed SDs from comparable trials using the same outcome measure, in accordance with established recommendations [70, 71]. In cases where a study reported the baseline SD and the SD of the change score but not the post‐intervention SD and where comparable trials using the same outcome measure were unavailable, post‐intervention SDs were estimated assuming a within‐person correlation (*r*) of 0.7 for the primary analysis, in line with recommendations from the Cochrane Handbook [69]. Sensitivity analyses were conducted assuming *r* values of 0.5 and 0.9. For two trials reporting post‐intervention means and SDs for subscales of the Pain Catastrophizing Scale (PCS) [42, 72], total post‐intervention means and SDs were calculated using variance sum law [73]. For one trial reporting separate results for participants with and without a history of recurrent depression [74] and another trial reporting separate results for participants favoring conservative versus surgical usual care [75], subgroup data were pooled using Cochrane Handbook guidelines [69].

Statistical heterogeneity was assessed through visual inspection of forest plots and evaluation of *I* ^2^ statistics. Sensitivity analyses examined (1) the effect of excluding studies with imputed SDs from meta‐analyses and (2) the robustness of results to the removal of outlier studies, defined as those with CIs not overlapping with the CI of the pooled effect [76]. For meta‐analyses involving ≥ 10 studies, publication bias was assessed using funnel plots of effect estimates from the included trials. Funnel plots were evaluated through visual inspection and Egger’s test. For meta‐analyses involving < 10 studies, publication bias was suspected if the body of evidence consisted of only small trials reporting favorable results and if eligible studies were identified in trial registries but remained unpublished [77].

Data analysis was performed using Comprehensive Meta‐Analysis (Version 4, Biostat, Englewood, NJ, 2022). STATA (Release 18, StataCorp LLC, College Station, TX) was used to generate forest and funnel plots. Data not included in the statistical synthesis were summarized narratively.

### 2.5. Assessment of Risk of Bias

Risk of bias in the included studies was assessed using the revised Cochrane risk of bias tool for randomized trials [78]. Two of four reviewers (TBH and FP, JP, or AA) independently applied the tool to the relevant outcomes in each included study and recorded justifications for domain‐level judgments of risk of bias (low, some concerns, or high). An overall judgment of risk of bias was determined for each of the outcomes assessed based on the highest level of risk of bias in any of the five domains. Any disagreements regarding judgments of risk of bias were resolved by consensus or arbitration by a third reviewer not involved in the initial assessment.

### 2.6. Assessment of Evidence Certainty

Two independent reviewers (TBH and LR) assessed the certainty of the evidence for each outcome using the Grading of Recommendations Assessment, Development, and Evaluation (GRADE) approach [79]. Certainty ratings were classified as high, moderate, low, or very low. All outcomes were initially rated as high certainty and were then downgraded based on four factors: (1) risk of bias (certainty downgraded one level when > 25% of participants in the meta‐analysis were from studies with a high risk of bias); (2) inconsistency (certainty downgraded one level when there was substantial heterogeneity, defined as *I* ^2^ > 50%); (3) imprecision (certainty downgraded one level when the pooled sample was < 400 participants); and (4) publication bias (certainty downgraded one level when publication bias was suspected, following the assessment methods described above). Indirectness was not considered as this review involves a specific population, relevant patient‐important outcomes, and direct comparisons. Disagreements regarding evidence certainty were resolved by consensus.

## 3. Results

### 3.1. Flow of Reports Through the Review

The literature search identified 20,706 reports. Following the removal of duplicates, 10,041 reports remained. After the title and abstract screening, 275 full‐text articles were obtained for consideration. Screening of these led to the identification of 51 studies that met the eligibility criteria and were included in the review. The flow of reports through the review is presented in Figure 1. Reasons for exclusion at the full‐text screening stage are detailed in Supporting Digital Content 2.

**FIGURE 1:**
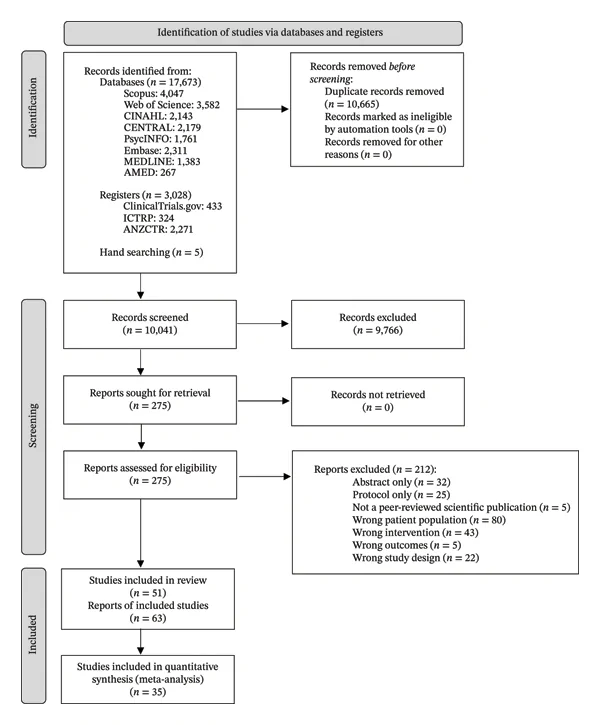
Flow of reports through the review.

Thirty‐five studies were included in one or more meta‐analyses. Seven studies were excluded from any meta‐analyses due to the absence of other studies with sufficiently similar comparators [80–86]. Nine studies were excluded from any meta‐analyses due to missing data [57, 87–94].

During full‐text screening, the authors of 19 reports were contacted to request additional information to clarify eligibility. Responses were received from nine authors and informed the exclusion of all nine reports [95–103]. The remaining 10 reports were excluded based on the available information (Supporting Digital Content 2) [104–113].

### 3.2. Characteristics of Included Studies

Detailed information regarding the characteristics of each included trial is presented in Supporting Digital Content 3.

#### 3.2.1. Participants

The pooled sample of this review included 5997 participants. Among trials reporting age, the mean age of participants was 51.7 years (range: 28.3–75.3). Seventeen trials included only female participants [42, 87, 88, 91, 114–126]. One trial did not report participant gender [89]. The remaining studies included both male and female participants. Overall, among the trials reporting gender, 73% of participants were female.

Seventeen trials recruited participants with chronic back pain [43, 44, 46, 57, 82, 84, 86, 87, 91, 93, 94, 114, 127–131]. Fifteen trials recruited participants with fibromyalgia [42, 72, 80, 81, 90, 115–117, 119, 120, 122, 124, 125, 132, 133]. Twelve trials recruited participants with arthritis [45, 64, 74, 83, 89, 118, 121, 134–138]. Four trials recruited participants with any type of chronic musculoskeletal pain [85, 88, 92, 139]. One trial recruited participants with chronic patellofemoral pain [123], one trial recruited participants with frozen shoulder [75], and one trial recruited participants with chronic painful temporomandibular disorder [126].

#### 3.2.2. Interventions

Most included trials used a broadly conventional mindfulness‐based program format, typically involving eight weekly group‐based sessions lasting between 1.5 and 2.5 h [40]; this format was used in 37 of 51 trials (72.5%). In 35 of these trials, sessions were delivered in person. One trial used live online group sessions [136], and one trial initially used in‐person group sessions before transitioning to live online group sessions due to public health orders related to the COVID‐19 pandemic [129].

A further nine trials delivered interventions that were similar to this conventional format, with mild variation in session frequency, session length, or number of sessions. Two trials delivered eight group‐based sessions over 4 weeks, with sessions held twice weekly; these sessions were delivered live online in Day et al. [84] and in‐person in Safavi‐Farokhi et al. [91]. Five trials delivered fewer weekly group‐based in‐person sessions: seven two‐hour sessions in Amutio et al. and Amutio et al. [42, 125], five 1.5‐h sessions in Pal et al. and Pal et al. [128, 139], and four one‐hour sessions in Zou et al. [83]. One trial, Liu et al. [75], delivered eight weekly sessions of shorter duration, with sessions lasting 50 min; the mode of delivery was not clearly reported. Finally, Taub et al. delivered 11 two‐hour group‐based in‐person sessions over 10 weeks [72].

Five trials used more distinct delivery formats. Zangi et al. delivered ten 4.5‐h group‐based in‐person sessions over 15 weeks [137]. Davis et al. delivered twelve 15‐min online asynchronous individual sessions over 6 weeks [90]. Sharpe et al. delivered five 20‐ to 30‐min online asynchronous individual sessions over 8 weeks [134]. Chaharmahali et al. delivered six weekly live online individual sessions, with the initial session lasting 45–60 min and subsequent sessions lasting 15 min [121]. Finally, Sannigrahi et al. delivered a 3‐month intervention involving four in‐person sessions with group and individual components and an unspecified number of online individual sessions; session lengths were not reported [118]. Across the included trials, eleven mindfulness‐based programs included a half‐ or full‐day silent retreat [44–46, 82, 116, 117, 122, 124, 131, 132, 135].

Program facilitators varied and included accredited MBSR and MBCT instructors (professional backgrounds not specified) (*n* = 15) [44–46, 94, 117, 119, 122, 124, 126–128, 131, 132, 135, 139], psychologists (mindfulness‐related qualifications not specified) (*n* = 10) [74, 75, 84, 87, 91, 93, 116, 121, 123, 130], “experienced mindfulness teachers” (qualifications not specified) (*n* = 7) [42, 57, 64, 82, 125, 129, 133], and healthcare professionals, including occupational therapists, physical therapists, social workers, medical doctors, and nurses, who received training in program delivery (*n* = 3) [75, 83, 137]. In one trial, the mindfulness‐based program was delivered by a study investigator who had attended unspecified courses and training [120]. In one trial, the mindfulness‐based program was jointly delivered by a clinical psychologist with extensive experience facilitating the program under investigation and a long‐term personal mindfulness meditation practice and a psychology student who assisted with session logistics [86]. In another, the mindfulness‐based program was jointly delivered by a physical therapist and a mindfulness teacher [136]. Twelve studies did not report who delivered the mindfulness‐based program [43, 72, 80, 81, 85, 88, 89, 92, 114, 115, 118, 138]. In 19 trials, the mindfulness‐based programs included content or practices specifically related to the experience of chronic pain [44, 57, 72, 75, 81, 85–87, 90, 93, 94, 119, 128–131, 133, 135, 136].

In all included studies, participants were encouraged to regularly practice the mindfulness meditation exercises taught during the program sessions. The dose of prescribed home practice ranged from ten to 60 min per day. Twenty‐one studies described providing audio recordings of guided meditations to facilitate home practice [44–46, 57, 64, 84, 86, 87, 90, 92, 94, 121–124, 126, 127, 131–133, 135]. Detailed intervention characteristics are presented in Supporting Digital Content 4.

#### 3.2.3. Comparators

One study employed a four‐arm design [93]. Twelve studies employed a three‐arm design [46, 74, 82, 84, 86, 92, 115, 117, 120, 121, 132, 134]. The remaining studies used a two‐arm design. Thirteen trials compared a mindfulness‐based program to a waitlist control [42, 44, 45, 72, 114, 116, 117, 120, 122, 125, 127, 134, 135]. Three trials compared a mindfulness‐based program to usual care [82, 89, 92], while nine trials compared a mindfulness‐based program plus usual care to usual care alone [46, 57, 64, 75, 87, 119, 124, 132, 137]. Six trials compared a mindfulness‐based program to no intervention [85, 88, 93, 115, 126, 131].

Seven trials compared a mindfulness‐based program to health and pain education [43, 74, 81, 90, 94, 128, 139]. Four trials assessed a mindfulness‐based program plus an exercise intervention against the exercise intervention alone [91, 118, 121, 123]. Five trials compared a mindfulness‐based program to CBT or a mindfulness‐based program plus usual care to CBT plus usual care [46, 74, 129, 133, 134]. In four of these trials, the mindfulness‐based programs and CBT interventions adopted group‐based formats [46, 74, 129, 133]. In the remaining trial, both interventions were delivered via online asynchronous individual sessions [134]. Three trials compared a mindfulness‐based program to cognitive therapy [84, 86, 93]. Two trials compared a mindfulness‐based program to behavior therapy [84, 93]. Finally, eleven trials compared a mindfulness‐based program to a distinct active comparator (Supporting Digital Content 3).

#### 3.2.4. Outcome Measures

Twenty‐one trials measured one or more pain‐related cognitions. Specifically, fourteen trials measured pain catastrophizing [42–44, 46, 72, 74, 123, 124, 126, 128, 131, 132, 136, 139], with the 13‐item PCS being the most used measure (*n* = 11). Seven trials measured pain self‐efficacy [43, 46, 74, 90, 94, 136, 137], with the Chronic Pain Self‐Efficacy Scale being the most used measure (*n* = 3). Seven trials measured chronic pain acceptance [44, 46, 127, 128, 130, 131, 139] using the Chronic Pain Acceptance Questionnaire (CPAQ) Revised (*n* = 4) and the CPAQ 8 (*n* = 3). Three trials measured kinesiophobia [44, 91, 123] using various self‐report measures (Supporting Digital Content 3), and two trials measured pain control [74, 85].

Depression was measured in 25 trials [44–46, 64, 72, 74, 81, 84, 86, 89, 93, 114, 116, 117, 119, 120, 124–126, 128, 131, 134–136, 138], with the depression subscale of the Depression, Anxiety, and Stress Scale‐21 Items being the most used measure (*n* = 6). Anxiety was measured in 17 trials [44, 46, 64, 80, 82, 84, 89, 114, 117, 124–126, 128, 131, 134, 135, 138], and psychological distress was measured in five trials [45, 115, 132, 133, 137], using various self‐report measures (Supporting Digital Content 3). Pain intensity was measured in 32 trials [43, 44, 46, 57, 64, 74, 75, 80, 84, 86–88, 90, 92–94, 115, 118, 119, 121–123, 127–130, 133–137, 139]. The most used measures were a visual analog scale (*n* = 10) and numerical rating scale (*n* = 9). Disability was measured in 23 trials [43, 46, 64, 72, 82–84, 86, 88, 91, 93, 94, 114, 118, 121–123, 127–131, 134], with the Roland–Morris Disability Questionnaire (*n* = 6) being the most used measure.

#### 3.2.5. Risk of Bias

Figures 2–10 and Supporting Digital Content 6 summarize the domain‐level and overall judgments of risk of bias for the relevant outcomes in each included study. All trials used random allocation; however, only 47% (24/51) reported adequate information for the reviewers to be confident that allocation was concealed. Intention‐to‐treat or modified intention‐to‐treat analysis was used in approximately 70% (35/51) of the studies. None of the included studies blinded the participants or persons delivering the intervention, which is a common limitation in studies involving psychosocial interventions. Approximately 55% of the outcomes assessed lacked complete follow‐up data. Overall, all outcomes assessed had some concerns or a high risk of bias.

**FIGURE 2:**
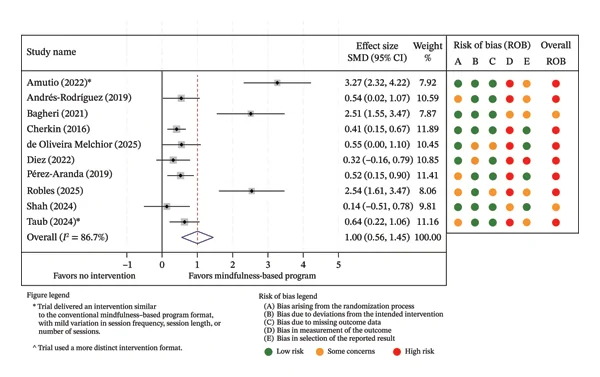
Forest plot with risk of bias summary for the effect of mindfulness‐based programs on pain catastrophizing immediately post‐intervention compared with no intervention.

**FIGURE 3:**
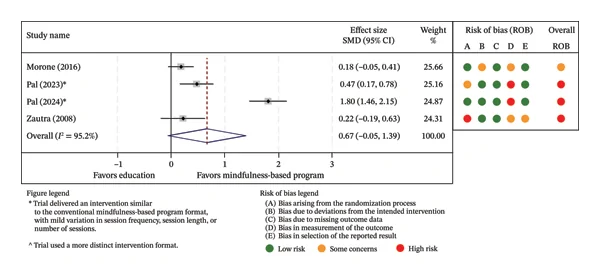
Forest plot with risk of bias summary for the effect of mindfulness‐based programs on pain catastrophizing immediately post‐intervention compared with education.

**FIGURE 4:**
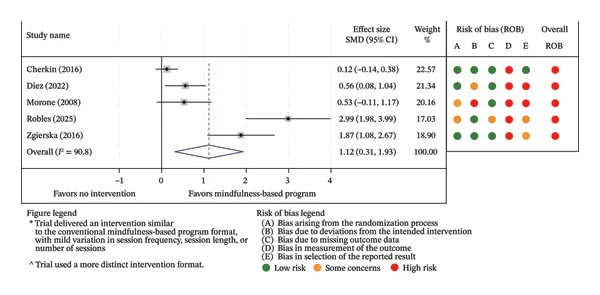
Forest plot with risk of bias summary for the effect of mindfulness‐based programs on chronic pain acceptance immediately post‐intervention compared with no intervention.

**FIGURE 5:**
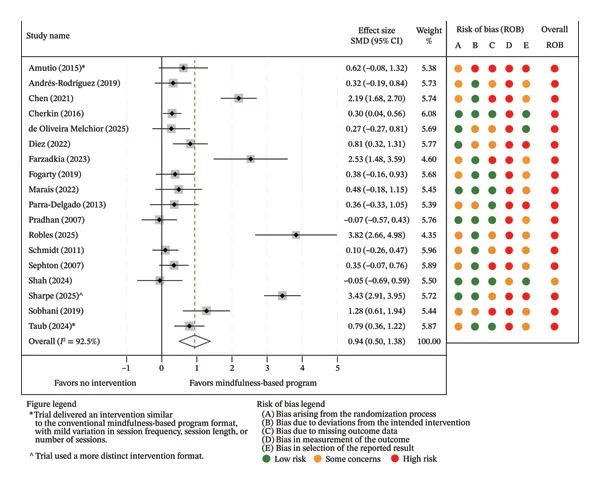
Forest plot with risk of bias summary for the effect of mindfulness‐based programs on depression immediately post‐intervention compared with no intervention.

**FIGURE 6:**
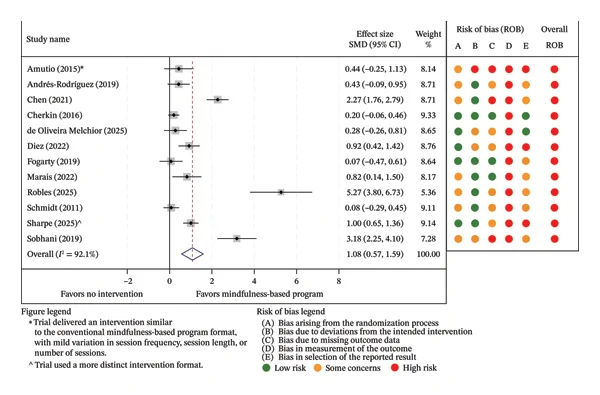
Forest plot with risk of bias summary for the effect of mindfulness‐based programs on anxiety immediately post‐intervention compared with no intervention.

**FIGURE 7:**
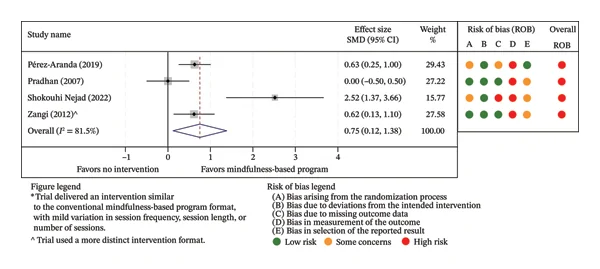
Forest plot with risk of bias summary for the effect of mindfulness‐based programs on psychological distress immediately post‐intervention compared with no intervention.

**FIGURE 8:**
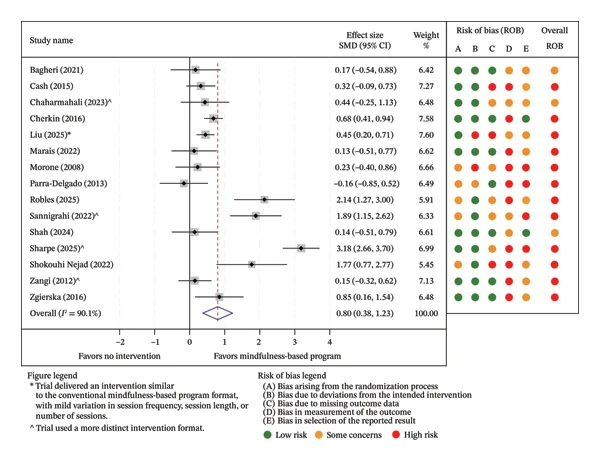
Forest plot with risk of bias summary for the effect of mindfulness‐based programs on pain intensity immediately post‐intervention compared with no intervention.

**FIGURE 9:**
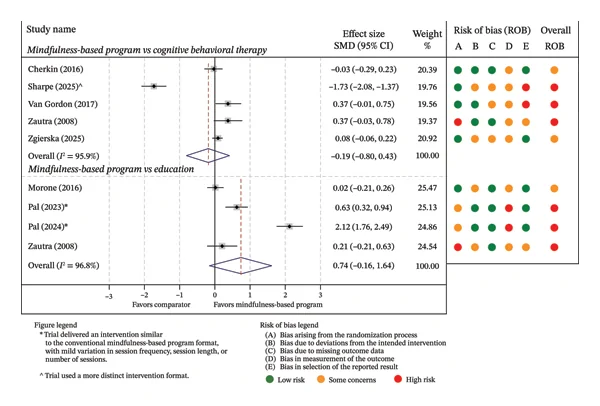
Forest plot with risk of bias summary for the effect of mindfulness‐based programs on pain intensity immediately post‐intervention compared with cognitive behavioral therapy and education.

**FIGURE 10:**
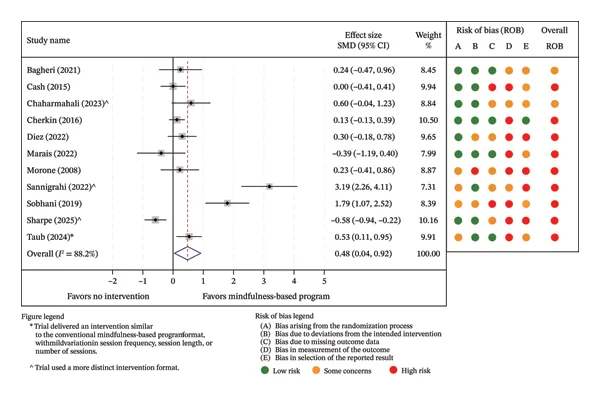
Forest plot with risk of bias summary for the effect of mindfulness‐based programs on disability immediately post‐intervention compared with no intervention.

### 3.3. Intervention Adherence

Across the included studies, reporting on intervention adherence varied considerably, preventing meta‐analysis. Twenty‐eight studies reported data on attendance at intervention sessions [43, 45, 46, 57, 64, 74, 81, 82, 86, 90, 92–94, 116–119, 122–124, 127, 128, 130, 132, 134–137]. Briefly, four studies found that 81%–91% of participants attended at least one session [46, 81, 130, 136]. Eight studies reported completion rates—defined as attending at least three‐quarters of the provided sessions—ranging from 43% to 100% [46, 64, 93, 119, 124, 132, 135, 136].

Eleven studies reported on adherence to home practice [42, 45, 64, 82, 94, 125, 127, 130, 132, 133, 136]. Briefly, five studies reported the frequency of home practice; this ranged from 2.5 to 5.1 days per week [94, 127, 130, 132, 136]. Four studies reported the average duration of home mindfulness meditation practices; this ranged from 31 to 53 min [94, 127, 132, 133]. Two studies measured home practice rates following completion of the mindfulness‐based program, reporting that 70%–90% of participants continued meditating regularly [64, 130].

Sixteen studies described strategies used to promote intervention adherence, focusing on either session attendance (*n* = 5) [80, 116, 122, 124, 134], home practice (*n* = 10) [42, 45, 93, 94, 125, 126, 128, 132, 133, 135], or both (*n* = 1) [123], though their effectiveness could not be determined due to limited reporting.

Further details are presented in Table 1.

**TABLE 1:** Descriptions of intervention adherence and strategies to promote adherence across the included studies.

Study	Adherence to intervention sessions	Adherence to home practice	Strategies to promote adherence
Akbarzadeh et al., 2022	—	—	Participants were verbally encouraged to attend all sessions.
Amutio et al., 2015	—	71% of participants reported having practiced the body‐scan homework, and 83% reported having practiced the mindfulness breathing homework.	Participants were encouraged to record their practice of the assigned homework exercises using a register sheet designed for this purpose.
Amutio et al., 2022	—	The mean degree of compliance with the body‐scan exercise was 68%, while that for the mindful breathing exercise was 79%.	Participants were encouraged to keep a daily record of their engagement with the home practices by means of a record sheet designed for this purpose.
Andrés‐Rodríguez et al., 2019	61.8% of participants (31/34) attended six or more sessions (out of a possible eight sessions). 50% of participants (17/34) attended the optional silent retreat session.	—	Minor modifications were made to the standard mindfulness‐based stress reduction program to better fit the participants’ needs and increase adherence.
Astin et al., 2003	81.2% of participants attended at least one session (out of a possible eight sessions).	—	—
Bagheri et al., 2021	The attendance rate for the mindfulness‐based program sessions was 100%.	—	When a participant had difficulty learning any skill, the mindfulness teacher spent more time with that person at the end of the session as needed.
Burns et al., 2022	57.3% of participants (82/143) attended six or more sessions (out of a possible eight sessions).	—	Participants were taught problem‐solving skills for identifying possible problems and developing solutions for dealing with obstacles related to practicing mindfulness. Participants developed a written maintenance plan that included a list of short‐term and long‐term goals for applying mindfulness methods and a plan for dealing with possible setbacks.
Cash et al., 2015	The mean number of mindfulness‐based program sessions attended was 5.5 (SD: 2.1) out of a possible eight sessions. 82.4% of participants (42/51) attended at least half of the sessions.	—	Participants who were absent from a session received a reminder phone call to attend subsequent sessions.
Cherkin et al., 2016	88.8% of participants (103/116) attended at least one mindfulness‐based program session. 50.9% of participants (59/116) attended at least six sessions. 25.8% of participants (30/116) attended the silent retreat.	—	—
Davis et al., 2013	The mean number of mindfulness‐based program sessions completed was 8.23 (SD: 4.57) out of a possible 12 sessions. 49% of participants (19/39) completed all 12 sessions.	—	—
Day et al., 2019	73.9% of participants in the mindfulness meditation group (17/23) and 91.3% of participants in the mindfulness‐based cognitive therapy group (21/23) attended four or more sessions (out of a possible eight sessions).	—	—
de Oliveira Melchior et al., 2025	—	—	Participants were provided with a diary to record their engagement with the home practices.
Donnino et al., 2021	The median number of mindfulness‐based program sessions attended was seven out of nine.	73% of participants spent between one and 6 hours per week on home practice. Two to 4 hours was the most common response, chosen 42% of the time.	—
Fogarty et al., 2019	80.8% of participants (21/26) completed the mindfulness‐based program, defined as attending at least six out of eight sessions. 76.9% (20/26) attended the silent retreat.	—	Participants were encouraged to keep a log of their home practice which was submitted to the mindfulness teacher at the start of each session.
Marais et al., 2022	90% of participants (18/20) attended six or more mindfulness‐based program sessions. 10% of participants (2/20) attended fewer than four sessions.	26.3% of participants (5/19) reported completing an average of 45 min of home practice daily, as prescribed. A further 26.3% of participants (5/19) reporting completing home practice daily for variable durations. 31.5% of participants (6/19) reported completing home practice 4‐5 times per week, while 10.5% (2/19) reported completing home practice 2‐3 times per week, and 5.3% (1/19) reported completing home practice 0‐1 times per week. 70% of participants (14/20) continued practicing mindfulness meditation two or more times per week in the 6 months following the completion of the mindfulness‐based program.	—
Morone et al., 2008	The mean number of mindfulness‐based program sessions attended was 6.7 (range: 5–8), out of a possible eight sessions.	Home mindfulness meditation was practiced, on average, on 4.3 days per week (range: 0–7), of a prescribed 6 days. The average minutes per day spent meditating was 31.6 (range: 0–52).	—
Morone et al., 2009	The mean number of mindfulness‐based program sessions attended was 7.5 (range: 6–8) out of a possible eight sessions.	Home mindfulness meditation was practiced, on average, on 5 days per week (range: 1–7), of a prescribed 6 days. The average minutes per day spent meditating was 31 (range: 22–48).	Participants were encouraged to maintain a log of their home practice.
Morone et al., 2016	The mean number of mindfulness‐based program sessions attended was 6.6 (range: 0–8), out of a possible eight sessions.	—	—
Pal et al., 2024	91.8% of participants (90/98) completed at least three out of five sessions.	—	Participants were encouraged to maintain a daily log of their home practice, which was monitored by the mindfulness teacher.
Parra‐Delgado and Latorre‐Postigo, 2023	The mean number of mindfulness‐based program sessions attended was 5.53 out of a possible eight sessions. 88.2% of participants completed the program, defined as attended at least four sessions. 58.8% of participants attended at least six sessions. 11.8% of participants attended all eight sessions.	—	—
Pérez‐Aranda et al., 2019	The mean number of mindfulness‐based program sessions attended was 5.0 (SD: 2.74) out of a possible eight sessions. 54.7% of participants (41/75) attended six or more sessions. 42.7% of participants (32/75) attended the optional silent retreat.	On average, participants practiced mindfulness meditation on 2.5 days per week (SD: 2.17), with a mean duration of 53 min per day of practice (SD: 49). 30.7% of participants (23/75) reported no home practice any day during the intervention, while 54.7% of participants (41/75) reported practicing on at least 2 days per week.	Participants were encouraged to record their home practice in a log. Practice experiences were reviewed, discussed, and reinforced during intervention sessions.
Plews‐Ogan et al., 2005	50% of participants (5/10) attended seven out of a possible eight sessions.	—	—
Pradhan et al., 2007	The median number of mindfulness‐based program sessions completed was eight (interquartile range: 6–8), out of a possible eight.	The mean total time spent on home practice was 47.12 h (SD: 21.26), or just over 1 hour per day on 6 days per week. Of 49 possible days on which practice could have been undertaken, the median was 42 days (interquartile range: 40–48).	Participants were encouraged to document the time spent on home practice per day.
Reiner et al., 2019	The mean number of mindfulness‐based program sessions completed was 6.3 (range: 5–8), out of a possible eight sessions.	—	—
Sannigrahi et al., 2022	—	Three participants did not practice the meditation regimen regularly.	—
Schmidt et al., 2011	The mean number of mindfulness‐based program sessions attended was 6.7 out of a possible eight sessions.	—	—
Sephton et al., 2007	82% of participants (42/51) were considered to have completed the mindfulness‐based program, defined as attending at least four out of eight sessions.	—	Absent participants received a reminder phone call to attend subsequent sessions.
Shah et al., 2024	On average, participants attended 54% of the mindfulness‐based program sessions. 43% of participants (9/21) attended at least six sessions. 19% of participants (4/21) did not attend any sessions.	On average, participants engaged in home practice on 4.9 days per week (95% CI: 4.5–5.3), of a prescribed 7 days.	—
Sharpe et al., 2025	The mean number of mindfulness‐based program sessions completed was 3.9 (SD: 1.48), out of a possible five sessions.	—	Telephone calls were placed to participants to encourage them to continue with the program, praise their efforts thus far, trouble shoot any difficulties, and answer any questions. If a participant missed a session, they received a reminder call to complete subsequent sessions.
Van Gordon et al., 2017	—	Participants practiced meditation for an average of 41.11 min per day (SD: 15.26).	Participants attended individual support sessions with the mindfulness teacher in weeks three and eight, during which they were guided on an individual basis to find the optimum frequency and duration of home meditation practice sessions.
Zangi et al., 2012	The mean number of mindfulness‐based program sessions attended was nine (range: 6–10), out of a possible ten sessions.	—	—
Zautra et al., 2008	The mean number of mindfulness‐based program sessions attended was 5.94 out of a possible eight sessions.	—	—
Zgierska et al., 2016	9.5% of participants (2/21) did not attend any mindfulness‐based program sessions.	During the intervention, participants performed 188.3 (SD: 94.4) minutes per week of formal practice and 110.1 (SD: 78.8) minutes per week of informal practice. During the intervention, formal mindfulness was practiced on 5.1 (SD: 2.1) days per week, and informal mindfulness was practiced on 4.9 (SD: 2.0) days per week. In the 18 weeks following the intervention, participants averaged 153.3 (SD: 139.6) minutes per week of formal practice and 99.5 (SD: 131.3) minutes per week of informal practice. In this period, formal mindfulness was practiced on 4.1 (SD: 2.6) days per week, and informal mindfulness was practiced on 4.2 (SD: 2.5) days per week. Overall, 90% of participants (18/20) continued mindfulness meditation practice during the 18 weeks following the intervention.	—

### 3.4. Effect of the Intervention

#### 3.4.1. The Effect of Mindfulness‐Based Programs on Pain‐Related Cognitions

Meta‐analyses were conducted to evaluate the immediate effects of mindfulness‐based programs on pain catastrophizing compared with no intervention and education. Due to limited data, the immediate effects of mindfulness‐based programs compared with CBT, as well as long‐term effects on pain catastrophizing, are presented narratively. A meta‐analysis was performed to examine the immediate effect of mindfulness‐based programs on chronic pain acceptance compared with no intervention. Due to limited data, the immediate effects compared with active controls and long‐term effects on chronic pain acceptance are reported narratively. Findings regarding the effects of mindfulness‐based programs on pain‐self efficacy are also summarized narratively. The immediate effects of mindfulness‐based programs on kinesiophobia compared with no intervention are synthesized narratively; no studies evaluated effects relative to active controls or long‐term outcomes. No meta‐analyses were conducted for pain control due to insufficient data; these findings are presented narratively.

##### 3.4.1.1. Pain Catastrophizing

###### 3.4.1.1.1. The Effect of Mindfulness‐Based Programs on Pain Catastrophizing Compared to No Intervention

The effect of mindfulness‐based programs on pain catastrophizing immediately post‐intervention compared with no intervention was estimated to be large (10 studies, 744 participants, SMD: 1.00, 95% CI: 0.56 to 1.45, *I* ^2^ = 86.7%, Figure 2). The certainty of evidence was rated as very low (Supporting Digital Content 5). A sensitivity analysis was performed with Cherkin et al. [46] removed to examine the effect of excluding studies with imputed SDs. This analysis also found that mindfulness‐based programs had a large effect (9 studies, 515 participants, SMD: 1.11, 95% CI: 0.57 to 1.65, *I* ^2^ = 87.4%, Supporting Digital Content 7). A second sensitivity analysis was performed with Amutio et al. [42], Bagheri et al. [123], and Robles et al. [44] removed, as these studies were identified as outliers. This analysis indicated a small effect of mindfulness‐based programs (7 studies, 644 participants, SMD: 0.38, 95% CI: 0.15–0.60), with moderately reduced heterogeneity (*I* ^2^ = 48.0%, Supporting Digital Content 7).

Two studies evaluated the long‐term effects of mindfulness‐based programs on pain catastrophizing compared with no intervention. Both Cherkin et al. [46] and Pérez‐Aranda et al. [132] reported moderate reductions in pain catastrophizing at 12 months post‐randomization among participants receiving the intervention, relative to those in the control group (see Supporting Digital Content 6).

###### 3.4.1.1.2. The Effect of Mindfulness‐Based Programs on Pain Catastrophizing Compared to Active Comparators

The effect of mindfulness‐based programs on pain catastrophizing immediately post‐intervention compared with education was examined by pooling four studies involving 719 participants. This meta‐analysis indicated a medium effect in favor of mindfulness‐based programs (SMD: 0.67). However, this estimate came with substantial heterogeneity and a CI that crossed the line of no effect, indicating the possibility of no significant difference between the interventions (95% CI: −0.05 to 1.39, *I* ^2^  = 95.2%, Figure 3). Evidence certainty was rated as low. A sensitivity analysis was performed with Pal et al. [128] removed, as this study was identified as an outlier. This analysis indicated a small effect in favor of mindfulness‐based programs (3 studies, 538 participants, SMD: 0.28, 95% CI: 0.10–0.47), with much lower heterogeneity (*I* ^2^ = 13.0%).

Two trials examined the effect of mindfulness‐based programs on pain catastrophizing compared to CBT. Zautra et al. [74] found that among participants with a history of recurrent depression, the mindfulness‐based program produced greater reductions in pain catastrophizing at post‐intervention than CBT; however, no significant effects were observed for participants without recurrent depression. Cherkin et al. [46] also reported that participants allocated to the mindfulness‐based program experienced greater improvements in pain catastrophizing at post‐intervention than participants allocated to CBT. At 12 months post‐randomization, both groups maintained statistically significant improvements, with no significant difference between the interventions [46] (see Supporting Digital Content 6).

Single studies examined the effects on pain catastrophizing of mindfulness‐based programs compared with additional comparators. These results are detailed in Supporting Digital Content 6.

##### 3.4.1.2. Chronic Pain Acceptance

###### 3.4.1.2.1. The Effect of Mindfulness‐Based Programs on Chronic Pain Acceptance Compared to No Intervention

The effect of mindfulness‐based programs on chronic pain acceptance immediately post‐intervention compared with no intervention was estimated to be large (5 studies, 400 participants, SMD: 1.12, 95% CI: 0.31 to 1.92, *I* ^2^  = 90.8%, Figure 4). The certainty of evidence for this outcome was rated as very low (Supporting Digital Content 5). For Cherkin et al. [46], post‐intervention SDs were estimated assuming *r* = 0.7. A sensitivity analysis assuming *r* = 0.9 produced minimal change in the results (SMD: 1.11, 95% CI: 0.29 to 1.93, *I* ^2^  = 91.1%, Supporting Digital Content 7). For the no intervention group, an assumed correlation of *r* = 0.5 was incompatible with the reported variability; the minimum compatible correlation for that group was approximately 0.67. A second sensitivity analysis was performed with Robles et al. [44] removed, as this study was identified as an outlier. This analysis found a medium effect of mindfulness‐based programs (4 studies, 368 participants, SMD: 0.69, 95% CI: 0.08 to 1.31, *I* ^2^  = 83.5%, Supporting Digital Content 7). Only one study examined the long‐term effect on chronic pain acceptance of a mindfulness‐based program compared with no intervention, reporting no significant difference between the groups at 12 months (see Supporting Digital Content 6) [46].

###### 3.4.1.2.2. The Effect of Mindfulness‐Based Programs on Chronic Pain Acceptance Compared to Active Comparators

Two studies examined the immediate effect on chronic pain acceptance of a mindfulness‐based program compared with education, reporting greater improvements in chronic pain acceptance among participants receiving the mindfulness‐based programs compared to the education groups [128, 139]. Only one trial examined the effect on chronic pain acceptance of a mindfulness‐based program compared with CBT. Cherkin et al. [46] reported no significant between‐group differences immediately post‐intervention or at 12 months (see Supporting Digital Content 6).

##### 3.4.1.3. Pain Self‐Efficacy

###### 3.4.1.3.1. The Effect of Mindfulness‐Based Programs on Pain Self‐Efficacy Compared to No Intervention

Three trials assessed the immediate effects of mindfulness‐based programs on pain self‐efficacy compared with no intervention. Cherkin et al. [46] and Zangi et al. [137] reported significantly greater improvements from baseline to post‐intervention in the mindfulness‐based program groups than in the control groups. In contrast, Shah et al. [136] found no significant between‐group difference.

Two trials examined long‐term effects. Cherkin et al. [46] reported no significant difference in pain self‐efficacy between the groups at 12 months post‐randomization, whereas Zangi et al. [137] observed a slight positive effect favoring the mindfulness‐based program (see Supporting Digital Content 6).

###### 3.4.1.3.2. The Effect of Mindfulness‐Based Programs on Pain Self‐Efficacy Compared to Active Comparators

Four trials compared mindfulness‐based programs with education for pain self‐efficacy immediately post‐intervention; however, a meta‐analysis was not performed due to missing data in two of the studies [90, 94]. Davis et al. [90], Morone et al. [43], and Zautra et al. [74] reported greater improvements in pain self‐efficacy in the mindfulness‐based program groups than in the education groups, while Morone et al. [94] reported that both groups improved in pain self‐efficacy at program completion, with no significant between‐group difference (see Supporting Digital Content 6). Two trials examined the effect of mindfulness‐based programs compared to CBT. Cherkin et al. [46] and Zautra et al. [74] found that both interventions improved pain self‐efficacy immediately post‐intervention, with no evidence of between‐group differences. Cherkin et al. [46] additionally examined the long‐term effect on pain self‐efficacy of a mindfulness‐based program and CBT, reporting that improvements were not maintained in either group at 12 months post‐randomization (see Supporting Digital Content 6).

##### 3.4.1.4. Kinesiophobia

Three trials examined the immediate effect of mindfulness‐based programs on kinesiophobia compared with no intervention. Bagheri et al. [123] and Safavi‐Farokhi et al. [91] reported significantly greater reductions in kinesiophobia from baseline to post‐intervention in the mindfulness‐based program groups than in the control groups. In contrast, Robles et al. [44] found no significant between‐group difference (see Supporting Digital Content 6). No studies examined long‐term effects on kinesiophobia or the effects of mindfulness‐based programs relative to active controls.

##### 3.4.1.5. Pain Control

One trial examined the effect on pain control of a mindfulness‐based program compared with no intervention. Brown and Jones [85] reported that participants in the mindfulness‐based program group experienced greater perceived control over pain at post‐intervention compared with participants allocated to no intervention (see Supporting Digital Content 6). One trial examined the effect on pain control immediately post‐intervention of (a) a mindfulness‐based program compared with education and (b) a mindfulness‐based program compared with CBT. Zautra et al. [74] reported that participants in the education group and CBT group experienced greater pain control post‐intervention than participants in the mindfulness‐based program group (see Supporting Digital Content 6).

#### 3.4.2. The Effect of Mindfulness‐Based Programs on Depression

A meta‐analysis was conducted to examine the immediate effect of mindfulness‐based programs on depression compared with no intervention. Comparisons with active controls are synthesized narratively. Findings for the long‐term effects are also reported narratively.

##### 3.4.2.1. The Effect of Mindfulness‐Based Programs on Depression Compared to No Intervention

The effect of mindfulness‐based programs on depression immediately post‐intervention compared with no intervention was estimated to be large (18 studies, 1277 participants, SMD: 0.94, 95% CI: 0.50 to 1.38, *I* ^2^ = 92.5%, Figure 5). Evidence certainty was rated as very low (Supporting Digital Content 5). A sensitivity analysis was performed with Cherkin et al. [46] removed to examine the effect of excluding studies with imputed SDs. This analysis also found that mindfulness‐based programs had a large effect (17 studies, 1048 participants, SMD: 0.99, 95% CI: 0.50 to 1.48, *I* ^2^ = 92.5%, Supporting Digital Content 7). A second sensitivity analysis was performed with six outlier studies removed [44, 45, 117, 120, 134, 138]. This analysis indicated a medium effect of mindfulness‐based programs (12 studies, 811 participants, SMD: 0.47, 95% CI: 0.30–0.64), with markedly reduced heterogeneity (*I* ^2^ = 28.2%). A single study that examined the long‐term effect on depression of a mindfulness‐based program compared with no intervention found that there was no significant difference between the groups at 12 months post‐randomization (see Supporting Digital Content 6) [46].

##### 3.4.2.2. The Effect of Mindfulness‐Based Programs on Depression Compared to Active Comparators

Three trials examined the immediate effects on depression of mindfulness‐based programs compared with CBT. Cherkin et al. [46] reported that participants randomized to receive CBT improved more than those randomized to MBSR at post‐intervention. Sharpe et al. [134] and Zautra et al. [74] reported that participants in both groups experienced an improvement in depression at post‐intervention, with no significant difference between the groups. The single study that examined the long‐term effect on depression of a mindfulness‐based program compared with CBT reported no significant difference between the groups at 12 months (see Supporting Digital Content 6) [46].

Two trials examined the immediate effect of a mindfulness‐based program compared with education on depression. Zautra et al. [74] reported that participants in both groups experienced an improvement in depression at post‐intervention, with no significant difference between the groups. Meanwhile, Pal et al. [128] reported that a statistically significant difference between groups was found, favoring the mindfulness‐based program (see Supporting Digital Content 6).

Single studies examined the effects on depression of mindfulness‐based programs compared with a diverse range of comparators. These results are detailed in Supporting Digital Content 6.

#### 3.4.3. The Effect of Mindfulness‐Based Programs on Anxiety

A meta‐analysis was conducted to examine the immediate effect of mindfulness‐based programs on anxiety compared with no intervention. Comparisons with active controls are synthesized narratively. Findings for the long‐term effects are also reported narratively.

##### 3.4.3.1. The Effect of Mindfulness‐Based Programs on Anxiety Compared to No Intervention

Mindfulness‐based programs were found to have a large effect on anxiety immediately post‐intervention compared with no intervention (12 studies, 946 participants, SMD: 1.08, 95% CI: 0.57 to 1.59, *I* ^2^ = 92.1%, Figure 6). Evidence certainty was rated as very low (Supporting Digital Content 5). For Cherkin et al. [46], post‐intervention SDs were estimated, assuming *r* = 0.7. A sensitivity analysis assuming *r* = 0.9 produced minimal change in the results (SMD: 1.08, 95% CI: 0.56 to 1.59, *I* ^2^ = 92.2%, Supporting Digital Content 7). For the intervention group, an assumed correlation of *r* = 0.5 was incompatible with the reported variability; the minimum compatible correlation for that group was approximately 0.64. A second sensitivity analysis was performed with five outlier studies removed [44, 46, 114, 117, 138]. This analysis found a medium effect of mindfulness‐based interventions (7 studies, 439 participants, SMD: 0.59, 95% CI: 0.30–0.88), with moderately reduced heterogeneity (*I* ^2^ = 52.1%, Supporting Digital Content 7). One study investigated the long‐term effect on anxiety of a mindfulness‐based program compared with no intervention, reporting no significant difference between the groups at 12 months (see Supporting Digital Content 6) [46].

##### 3.4.3.2. The Effect of Mindfulness‐Based Programs on Anxiety Compared to Active Comparators

Two trials compared mindfulness‐based programs with CBT for anxiety. Both reported no significant difference between the groups immediately post‐intervention [46, 134]. The single study that examined the long‐term effect on anxiety of a mindfulness‐based program compared with CBT also found no significant between‐group difference at 12 months post‐randomization (see Supporting Digital Content 6) [46].

Only one trial examined the effect on anxiety of a mindfulness‐based program compared with education, reporting that the mindfulness‐based program yielded moderately larger reductions in anxiety post‐intervention than the education program (see Supporting Digital Content 6) [128].

Single studies examined the effects on anxiety of mindfulness‐based programs compared with a diverse range of comparators. These results are detailed in Supporting Digital Content 6.

#### 3.4.4. The Effect of Mindfulness‐Based Programs on Psychological Distress

The effect of mindfulness‐based programs on psychological distress immediately post‐intervention compared with no intervention was estimated to be large (4 studies, 261 participants, SMD: 0.75, 95% CI: 0.12 to 1.38, *I* ^2^ = 81.5%, Figure 7). Evidence certainty was rated as very low (Supporting Digital Content 5). Two studies examined the long‐term effect on psychological distress of mindfulness‐based programs compared with no intervention, with both studies reporting that the mindfulness‐based programs had a moderate effect at 12 months post‐randomization compared with no intervention (see Supporting Digital Content 6) [132, 137].

Single studies examined the effects on psychological distress of mindfulness‐based programs compared with a diverse range of comparators. These results are detailed in Supporting Digital Content 6.

#### 3.4.5. The Effect of Mindfulness‐Based Programs on Pain Intensity

Three meta‐analyses were conducted to examine the immediate effects of mindfulness‐based programs on pain intensity compared with no intervention, education, and CBT. Findings for the long‐term effects are reported narratively. Comparisons with additional active comparators were also synthesized narratively.

##### 3.4.5.1. The Effect of Mindfulness‐Based Programs on Pain Intensity Compared to No Intervention

The effect of mindfulness‐based programs on pain intensity immediately post‐intervention compared with no intervention was estimated to be large (15 studies, 1090 participants, SMD: 0.80, 95% CI: 0.38 to 1.23, *I* ^2^ = 90.1%, Figure 8). The certainty of evidence was rated as low (Supporting Digital Content 5). A sensitivity analysis was performed with Cherkin et al. [46] and Zgierska et al. [130] removed to examine the effect of excluding studies with imputed SDs. This analysis also found that mindfulness‐based programs had a large effect (13 studies, 826 participants, SMD: 0.81, 95% CI: 0.28 to 1.34, *I* ^2^ = 91.5%, Supporting Digital Content 7). A second sensitivity analysis was performed with Robles et al. [44] and Sharpe et al. [134] removed, as these studies were identified as outliers. This analysis indicated a medium effect of mindfulness‐based interventions (13 studies, 927 participants, SMD: 0.49, 95% CI: 0.24–0.73), with moderately reduced heterogeneity (*I* ^2^ = 63.8%). Two trials examined the long‐term effect on pain intensity of mindfulness‐based programs compared with no intervention. Cherkin et al. [46] reported greater improvement in pain intensity at 12 months in the MBSR group. In contrast, Zangi et al. [137] reported no significant difference between the two groups (see Supporting Digital Content 6).

##### 3.4.5.2. The Effect of Mindfulness‐Based Programs on Pain Intensity Compared to Active Comparators

The pooled effect of mindfulness‐based programs on pain intensity immediately post‐intervention compared with CBT was examined by pooling five studies involving 1365 participants. This analysis indicated a small effect in favor of CBT (SMD: −0.19). However, the estimate came with a wide CI that crossed the line of no effect (95% CI: −0.80 to 0.43, *I* ^2^ = 95.9%, Figure 9). Evidence certainty was rated as low (Supporting Digital Content 5). For Cherkin et al. [46], post‐intervention SDs were estimated assuming *r* = 0.7. Sensitivity analyses assuming *r* = 0.5 and *r* = 0.9 produced no change in the results (Supporting Digital Content 7). A further sensitivity analysis was performed with Sharpe et al. [134] removed, as this study was identified as an outlier. This analysis indicated a trivial effect in favor of mindfulness‐based interventions with reduced heterogeneity; however, the wide CI remained (4 studies, 1198 participants, SMD: 0.13, 95% CI: −0.03 to 0.30, *I* ^2^ = 36.1%, Supporting Digital Content 7). Two studies examined the long‐term effect on pain intensity of a mindfulness‐based program compared with CBT. Cherkin et al. [46] and Zgierska et al. [129] reported that participants in both groups experienced an improvement in pain intensity at 12 months, with no significant difference between the groups (see Supporting Digital Content 6).

The effect of mindfulness‐based programs on pain intensity immediately post‐intervention compared with education was examined by pooling four studies with 715 participants. The SMD was 0.74 indicating a medium effect in favor of mindfulness‐based programs. However, that estimate came with substantial heterogeneity and a wide CI that crossed the line of no effect (95% CI: −0.16 to 1.64, *I* ^2^ = 96.8%, Figure 9). The certainty of evidence for this outcome was rated as low (Supporting Digital Content 5). A sensitivity analysis was performed with Pal et al. [128] removed, as this study was identified as an outlier. This analysis indicated a small effect in favor of mindfulness‐based interventions with mildly reduced heterogeneity; however, the wide CI remained (534 participants, 3 studies, SMD: 0.28, 95% CI: −0.11 to 0.67, *I* ^2^ = 78.4%, Supporting Digital Content 7).

Single studies examined the effects on pain intensity of mindfulness‐based programs compared with a diverse range of comparators. These results are detailed in Supporting Digital Content 6.

#### 3.4.6. The Effect of Mindfulness‐Based Programs on Disability

A meta‐analysis was conducted to examine the immediate effect of mindfulness‐based programs on disability compared with no intervention. Comparisons with active controls are synthesized narratively. Findings for the long‐term effects are also reported narratively.

##### 3.4.6.1. The Effect of Mindfulness‐Based Programs on Disability Compared to No Intervention

Mindfulness‐based programs compared with no intervention had a medium effect on disability immediately post‐intervention (11 studies, 815 participants, SMD: 0.48, 95% CI: 0.04 to 0.92, *I* ^2^ = 88.2%, Figure 10). The certainty of evidence for this outcome was rated as very low (Supporting Digital Content 5). A sensitivity analysis was performed with Cherkin et al. [46] removed to examine the effect of excluding studies with imputed SDs. This analysis also found that mindfulness‐based programs had a medium effect (10 studies, 586 participants, SMD: 0.54, 95% CI: 0.01 to 1.07, *I* ^2^ = 89.3%, Supporting Digital Content 7). A second sensitivity analysis was performed with three outlier studies removed [114, 118, 134]. This analysis found mindfulness‐based programs to have a small effect on disability immediately post‐intervention compared with no intervention (8 studies, 604 participants, SMD: 0.21, 95% CI: 0.04–0.38), with significantly reduced heterogeneity (*I* ^2^ = 5.4%).

Only one trial examined the long‐term effect of a mindfulness‐based program on disability compared with no intervention. Cherkin et al. [46] found that participants randomized to receive MBSR were more likely than those randomized to no intervention to show clinically meaningful improvement in disability at 12 months post‐randomization. The difference was not maintained at the 2‐year follow‐up (see Supporting Digital Content 6).

##### 3.4.6.2. The Effect of Mindfulness‐Based Programs on Disability Compared to Active Comparators

Three trials examined the effect on disability immediately post‐intervention of a mindfulness‐based program compared with CBT, all reporting improvements in disability in both groups, with no significant difference between the groups [46, 129, 134]. Similarly, the two trials that examined the long‐term effect on disability of a mindfulness‐based program compared with CBT found no significant difference between the groups at 12 months (see Supporting Digital Content 6) [46, 129].

Three trials examined the effect on disability immediately post‐intervention of a mindfulness‐based program compared with education. Morone et al. [43] and Pal et al. [128] reported slightly greater improvements among the mindfulness‐based program participants. Morone et al. [94] reported improvements in disability in both groups, with no significant difference between the groups (see Supporting Digital Content 6).

Single studies examined the effects on disability of mindfulness‐based programs compared with a diverse range of comparators. These results are detailed in Supporting Digital Content 6.

#### 3.4.7. MRAEs

Seventeen trials reported information on MRAEs [43, 45, 46, 84, 86, 94, 123, 127, 129–132, 134–137, 139]. The methods used for monitoring MRAEs and the specific MRAEs reported in each study are presented in Table 2. All reported MRAEs were mild in nature.

**TABLE 2:** Mindfulness‐related adverse events reported by mindfulness‐based program participants across the included studies.

Study	Method used to measure adverse events	Adverse events reported by mindfulness‐based program participants
Bagheri et al., 2021	Participants instructed to report any adverse events experienced during the study period to the investigators.	Two participants reported temporarily increased pain with mindful movement. No serious adverse events were reported.
Cherkin et al., 2016	Participants asked whether they had experienced any adverse events at each intervention session.	Twenty‐nine percent (30/103) of participants who attended at least one session reported experiencing an adverse event; the most common complaint was temporarily increased pain with mindful movement. No serious adverse events were reported.
Day et al., 2019	Structured, weekly assessments inquired into adverse events and negative effects associated with the mindfulness‐based program. Identified adverse events were documented in accordance with Human Research Ethics Committee (HREC) ethical policies, and HREC staff evaluated if any serious adverse event was study related.	Two mindfulness meditation group participants and two mindfulness‐based cognitive therapy group participants reported an adverse event. All events were deemed unrelated to participation in the trial.
Day et al., 2024	Participants asked whether they had experienced any adverse events at each intervention session.	No adverse events related to the mindfulness‐based program were reported.
Diez et al., 2022	Questionnaires administered every week to evaluate possible adverse effects of the mindfulness‐based program.	Not reported.
Fogarty et al., 2019	Not reported.	No adverse events were reported.
Morone et al., 2008	Not reported.	No serious adverse events were reported.
Morone et al., 2009	Not reported.	No adverse events were reported.
Morone et al., 2016	Not reported.	No adverse events related to the mindfulness‐based program were reported.
Pal et al., 2023	Participants instructed to report any adverse events experienced during the study period to the investigators.	No adverse events were reported.
Pérez‐Aranda et al., 2019	Use of an ad hoc “adverse events of the intervention” checklist.	Three participants reported experiencing notable adverse events during and/or after the intervention period with significant frequency: mild fatigue (*n* = 1), intense palpitations (*n* = 1), and a variety of symptoms including fatigue, tension headaches, dizziness, somnolence, weight gain, and loss of sexual desire (*n* = 1). It was not possible to verify whether these adverse events were related to the mindfulness‐based program. Five participants reported experiencing adverse events after the intervention period at a very low frequency and intensity.
Pradhan et al., 2007	Participants asked whether they had experienced any adverse events at each intervention session.	No study‐related adverse events were reported.
Shah et al., 2024	Not reported.	No study‐related adverse events were reported.
Sharpe et al., 2025	Adverse events recorded only if they were reported spontaneously by participants.	Fourteen percent [95% CI: 12 to 16] of the participants reported a worsening of their symptoms during the intervention period. Only one of these participants attributed their worsening in symptoms to the mindfulness‐based program.
Zangi et al., 2012	Not reported.	No adverse events were reported.
Zgierska et al., 2016	Participants asked whether they had experienced any adverse events at each intervention session.	Anticipated, mild, and self‐limited side effects were reported.
Zgierska et al., 2025	Adverse event information was systematically collected at each assessment and inquired about at each contact.	No serious adverse events were reported.

Only two trials described how MRAEs were managed. Bagheri et al. [123] referred participants to an independent physician for clinical assessment and initiation of the appropriate course of action. Day et al. [84] followed individual adverse events until the event resolved or study participation ended. None of the studies that reported MRAEs indicated whether these led to nonadherence or withdrawal from the intervention.

### 3.5. Publication Bias

Publication bias was assessed statistically for the meta‐analyses examining the effects of mindfulness‐based programs on pain catastrophizing, depression, anxiety, pain intensity, and disability compared with no intervention. Funnel plot inspection for pain catastrophizing, depression, anxiety, and disability revealed mild asymmetry, and Egger’s test indicated potential publication bias (Supporting Digital Content 8 and 9). No evidence of publication bias was observed for pain intensity. To assess potential publication bias for outcomes not covered by statistical tests, the specified clinical trial registries were searched for unpublished relevant trials completed three or more years prior to January 9, 2026. One such trial was identified, evaluating the effect of mindfulness‐based programs on chronic pain acceptance compared with no intervention [140]. Given the limited number of studies included in the meta‐analysis examining the effect of mindfulness‐based programs on chronic pain acceptance compared with no intervention, the absence of this trial may have introduced bias.

A summary of findings table outlining the key findings of this review is provided in Supporting Digital Content 9.

## 4. Discussion

This systematic review and meta‐analysis of RCTs evaluated the effects of mindfulness‐based programs on pain‐related cognitions in individuals with chronic musculoskeletal pain. Compared to no intervention, mindfulness‐based programs reduced pain catastrophizing and improved chronic pain acceptance immediately post‐intervention. Mindfulness‐based programs may additionally improve pain self‐efficacy and pain control and reduce kinesiophobia. Minimal significant differences were observed in the effect on pain‐related cognitions of mindfulness‐based programs compared to CBT. Mindfulness‐based programs may offer benefits beyond education for reducing pain catastrophizing and improving chronic pain acceptance and pain self‐efficacy; however, current evidence is limited.

Beyond pain‐related cognitions, mindfulness‐based programs were associated with large improvements in depression, anxiety, psychological distress, and pain intensity and a medium improvement in disability immediately post‐intervention compared to no intervention. Comparisons with CBT suggest that mindfulness‐based programs likely have comparable effects on these outcomes. However, the certainty of evidence is very low. Comparisons with education for these outcomes were limited with inconsistent findings.

The potential comparable efficacy of mindfulness‐based programs and CBT, as delivered in the included studies, could have important implications for health system planning. CBT is typically delivered by mental health professionals; however, a shortage of these professionals in many countries has limited access to care [32, 33]. In contrast, mindfulness‐based programs can be facilitated by practitioners from a broader range of professional backgrounds—including health, social work, and education—potentially making them a more scalable option [141, 142]. Incorporating these programs into healthcare systems could improve access to psychological support for people with chronic musculoskeletal pain. There is a need for further studies that examine the relative efficacy and resource implications of mindfulness‐based programs, delivered in their conventional group‐based format, compared with CBT delivered in its conventional one‐on‐one format. Further research is also needed to explore the implementation of mindfulness‐based programs in real‐world healthcare settings.

The observed effects of mindfulness‐based programs on pain‐related cognitions may reflect several interconnected biopsychosocial mechanisms. Mindfulness‐based programs encourage individuals to attend to sensations without judgment and to disengage from automatic appraisals of pain as inherently negative or threatening [38, 143]. This shift may reduce resistance to unpleasant sensations and experiential avoidance, thereby enhancing pain acceptance [144]. Cultivating nonjudgment and nonreactivity may also attenuate the magnification of negative appraisals, reducing pain catastrophizing [145]. Training attention regulation through the practice of present‐moment awareness may further reduce pain catastrophizing by disrupting rumination and future‐oriented thought patterns concerning pain worsening [146, 147].

These psychological processes may occur alongside changes in neurobiological systems involved in the affective appraisal and cognitive modulation of pain. For example, mindfulness meditation has been associated with structural changes in the amygdala [148] and altered activation in brain regions such as the orbitofrontal, subgenual anterior cingulate, and anterior insular cortices [149, 150]. These changes could plausibly reduce negative appraisals of pain and reduce the degree to which pain is experienced as threatening. Mindfulness meditation may also enhance parasympathetic regulation [151] and has been associated with increased oxytocin concentrations among people with chronic pain [152]. These changes could contribute to reduced physiological arousal and stress reactivity and support feelings of safety and contentment [153, 154]. However, their direct roles in modifying pain catastrophizing and pain acceptance have not been established.

Social processes may also be relevant, particularly when mindfulness‐based programs are delivered in groups. Shared experiences and social validation may normalize pain‐related experiences, while positive peer modeling may challenge beliefs that pain is unmanageable or inevitably disabling [155, 156]. These processes may, in turn, reduce pain catastrophizing and support greater pain acceptance.

The comparable effects of mindfulness‐based programs and CBT may reflect the presence of shared mechanisms, despite the unique approaches of the interventions. For instance, mindfulness‐based programs foster cognitive reappraisal by encouraging nonjudgmental awareness, whereas CBT employs behavioral experiments [157, 158]. Similarly, emotional regulation may be enhanced across modalities, with mindfulness‐based programs cultivating equanimity and CBT teaching adaptive coping strategies [159].

Recognizing that different approaches can produce comparable outcomes may support a more personalized model of pain management, wherein intervention selection is guided by patient preference and accessibility, rather than efficacy alone. Identifying patient characteristics that predict optimal response to specific interventions could further facilitate the provision of tailored care. Although preliminary moderation analyses have identified a limited set of such characteristics within the chronic musculoskeletal pain population [74, 160, 161], further research is needed to comprehensively address the challenge of matching individuals to the most appropriate psychosocial intervention.

The exception to the patterns described above is perceived pain control, with preliminary evidence suggesting that education and CBT may be more effective than mindfulness‐based programs in improving this outcome. This may reflect the philosophical differences between approaches, with mindfulness‐based programs placing greater emphasis on attending to pain with acceptance [38], whereas education and CBT often foreground active pain management more explicitly [157, 162, 163].

The described findings are consistent with previous meta‐analyses focused on specific musculoskeletal pain conditions, including chronic back pain [47, 164, 165], rheumatoid arthritis [166], and fibromyalgia [58, 167], which suggest that mindfulness‐informed interventions are effective in improving clinical outcomes compared to passive controls and demonstrate comparable efficacy to active controls. The present review extends these findings to all chronic musculoskeletal pain conditions. By limiting inclusion to interventions that meet established criteria for mindfulness‐based programs, specifically those in which mindfulness meditation training is the central therapeutic component [39], and by conducting separate meta‐analyses for comparisons with distinct active controls, this review enhances the specificity and interpretability of the evidence base. Furthermore, the exclusive inclusion of RCTs contributes higher quality evidence to the field.

The long‐term effects of mindfulness‐based programs remain unclear due to a lack of evidence. Chronic musculoskeletal pain requires management strategies that facilitate lasting change. Further investigation into the long‐term efficacy of mindfulness‐based programs in individuals with chronic musculoskeletal pain is warranted. Similarly, few of the studies included in this review employed an online mode of delivery. Internet‐based interventions are a promising approach to scaling up access to psychological care. While emerging evidence indicates that live online group‐based mindfulness programs can be as effective as in‐person group‐based programs [84, 168], further research is needed to evaluate the efficacy and safety of asynchronous online delivery.

Regarding intervention adherence, significant variability was seen between and within studies. Several studies reported associations between greater engagement in mindfulness practice and greater improvement in outcomes [122, 130, 132, 133]. This trend has been observed in other clinical and nonclinical populations [169]. These associations may reflect a dose–response relationship, whereby greater engagement in practice contributes to greater improvement. Alternatively, individuals who experience early benefit may perceive mindfulness practice as more acceptable or useful and therefore be more likely to practice. It is also possible that both explanations contribute, or that participant characteristics influence both adherence and response to mindfulness practice. Future research should clarify the direction and nature of the relationship between adherence and outcomes, including whether greater engagement in practice contributes to improved outcomes, whether adherence is a marker of early response or intervention suitability, and which participant characteristics may predict sustained engagement and benefit. Such work may inform strategies to support engagement, such as health coaching techniques, which have shown promise in improving engagement with various health behaviors among individuals with chronic pain [170–172].

Assessment of MRAEs was insufficient across the included trials. Most trials did not specify how MRAEs were monitored. Those that did primarily relied on spontaneous reporting—a method known to underestimate adverse event frequency [173, 174]. As such, an evaluation of the safety of mindfulness‐based programs for people with chronic musculoskeletal pain was not possible. Given that there are published reports of serious and distressing MRAEs [174–176], future trials should actively and systematically measure them. The use of instruments such as the Meditation‐Related Adverse Effects Scale‐Mindfulness Based Program version [177] or the Inventory of Meditation Experiences [178] is recommended.

A further limitation of the existing evidence base that should be considered is the moderate‐to‐high risk of bias ratings that all assessed outcomes received. This was primarily due to a lack of blinding of participants and individuals delivering the mindfulness‐based programs. While double‐blinding is not feasible when assessing mindfulness‐based programs, bias can be reduced by comparing to an active control that either represents an empirically established intervention or matches the mindfulness‐based program on all nonspecific factors such as structure and dosage. An example of the latter is a “sham mindfulness meditation” intervention that trains individuals to “take slow, deep breaths” in a meditative posture but omits the mindfulness‐specific instructions—such as attending to the breath in a nonjudgmental manner—hypothesized to mediate pain relief [149, 179, 180]. Additionally, participants should be blinded to which arm is the experimental intervention.

This review also has limitations that warrant consideration. First, substantial heterogeneity was observed in many of the meta‐analyses, suggesting variability in treatment effects across the included studies. High heterogeneity reduces the interpretability of a single pooled estimate and suggests that treatment effects may vary across subpopulations or intervention formats. One possible contributor to this heterogeneity is variability in intervention duration and the timing of post‐intervention outcome assessment. As post‐intervention outcomes were assessed at the end of each study’s intervention period rather than at a uniform time point from baseline, the pooled estimates combined outcomes measured after different periods of treatment exposure. In addition, although all interventions involved mindfulness meditation as the central pedagogical component and were consistent with Crane et al.’s definition of a mindfulness‐based program [39], some varied in their content or therapeutic emphasis, including the incorporation of additional practices or chronic pain‐specific adaptations. This variability may have contributed to the heterogeneity observed in treatment effects across studies. Investigation of heterogeneity using meta‐regression or subgroup analysis was not possible due to an inadequate number of studies in the meta‐analyses and uneven distribution of relevant covariates [181]. Second, several of the meta‐analyses included only a small number of studies. While two studies make a meta‐analysis possible [182], larger sample sizes typically lead to stronger conclusions. Third, publication bias could not be assessed statistically for many of the outcomes as fewer than ten studies were available for analysis [183, 184]; however, current guidelines for managing this limitation were followed.

Despite these limitations, this systematic review has notable strengths. It is the first systematic review and meta‐analysis of RCTs to evaluate the effect of mindfulness‐based programs on pain‐related cognitions in individuals with chronic musculoskeletal pain. This review was conducted in accordance with PRISMA guidelines and followed a prospectively registered protocol. A comprehensive search strategy was used. Additionally, sensitivity analyses were performed to assess the robustness of the results to the removal of outlier studies and studies with imputed data.

## 5. Conclusion

This systematic review and meta‐analysis found that mindfulness‐based programs can reduce pain catastrophizing and improve chronic pain acceptance immediately post‐intervention compared to no intervention in individuals with chronic musculoskeletal pain. Additionally, mindfulness‐based programs were associated with reductions in depression, anxiety, psychological distress, pain intensity, and disability. Mindfulness‐based programs demonstrated, at a minimum, comparable effects to established psychosocial interventions, including education and CBT. This finding is particularly relevant in the context of persistent barriers to accessing psychological care and the potential for mindfulness‐based programs to offer an additional option. However, the evidence was limited. There is a need for further trials that compare mindfulness‐based programs to empirically established interventions. Future research should also continue investigating the potential of internet‐delivered mindfulness‐based programs to broaden reach. Evidence certainty was generally low, and the long‐term effects of mindfulness‐based programs remain unclear. Intervention adherence varied; future research should clarify the direction and nature of the relationship between adherence and outcomes. Furthermore, the view that mindfulness‐based programs are associated with minimal adverse events is not well supported by current trials, due to limited reporting.

## Author Contributions

Talia Barnet‐Hepples: conceptualization, methodology, validation, formal analysis, investigation, data curation, writing–original draft, visualization, and project administration; Fereshteh Pourkazemi: conceptualization, methodology, formal analysis, investigation, writing–review and editing, and supervision; Evangelos Pappas: conceptualization, methodology, investigation, writing–review and editing, and supervision; Linting Ruan: validation, formal analysis, investigation, and writing–review and editing; James Puterflam: formal analysis, investigation, and writing–review and editing; Jemma Masterton: investigation and writing–review and editing; and Anita Amorim: conceptualization, methodology, formal analysis, investigation, writing–review and editing, and supervision.

## Funding

This research was supported by an Australian Government Research Training Program (RTP) Scholarship. Open‐access publishing was facilitated by the University of Sydney, as part of the Wiley–the University of Sydney agreement via the Council of Australasian University Librarians.

## Disclosure

An oral presentation of this work was delivered at the 2026 Australian Pain Society 46th Annual Scientific Meeting, held 19–22 April 2026 at the Adelaide Convention Centre, Adelaide, Australia [185]. The funder played no role in the design, conduct, or analysis of the study, nor in the interpretation and reporting of the study findings.

## Ethics Statement

The authors have nothing to report.

## Conflicts of Interest

Anita Amorim is a member of the advisory board for OpenGround, an Australian charity organization that delivers mindfulness meditation and compassion programs. This role is without remuneration. The author does not receive any financial or other compensation. The other authors declare no conflicts of interest.

## Supporting Information

Additional supporting information can be found online in the Supporting Information section.

## Supporting information


**Supporting Information 1** Supporting Digital Content 1: Search Strategies. Supporting Digital Content 2: List of Reports Excluded During Full‐Text Screening with Reasons for Exclusion. Supporting Digital Content 3: Summary of the Characteristics of the Included Studies. Supporting Digital Content 4: Summary of the Characteristics of Interventions in the Included Studies. Supporting Digital Content 5: GRADE Evidence Profile. Supporting Digital Content 6: Supporting Findings. Supporting Digital Content 7: Sensitivity Analyses. Supporting Digital Content 8: Funnel Plots of Standard Error by Hedges’ g. Supporting Digital Content 9: Summary of Findings.

**Supporting Information 2** PRISMA Checklist.

## Data Availability

Additional data that support the findings of this study are available from the corresponding author (TBH) upon reasonable request.
