# Supplementary material for: The Effects of Mindfulness‐Based Programs on Pain‐Related Cognitions in Individuals With Chronic Musculoskeletal Pain: A Systematic Review and Meta‐Analysis
Source: Pain Res Manag. 2026 Nov 9;2026:6495015. doi: 10.1155/prm/6495015 (PMC13746321; doi:10.1155/prm/6495015)
Supplement: Supplementary file 1 [file PRM-2026-6495015-s001.zip › Supplemental Digital Content 1_Search Strategies_v4_09012026.pdf]

Supplemental Digital Content 1: Search Strategies

Ovid MEDLINE(R) ALL <1946 to January 07, 2026>

1 Mindfulness/ 8587
2 Meditation/ 4343
3 mindful*.mp. 23344
4 meditat*.mp. 11497
5 third wave.mp. 2040
6 mind body.mp. 7669
7 stress reduction.mp. 5941
8 vipassana.mp. 90
9 satipatthana.mp. 3
10 anapanasati.mp. 13
11 1 or 2 or 3 or 4 or 5 or 6 or 7 or 8 or 9 or 10 41983
12 Chronic Pain/ 28724
13 ((chronic* or persist* or recur* or ongoing*) adj5 pain*).mp. 146162
14 Musculoskeletal Pain/ 5420
15 (musculoskeletal* or bone* or cartilage* or joint* or muscular*).mp. 2012353
16 musculoskeletal diseases/ or bone diseases/ or cartilage diseases/ or joint diseases/ or muscular diseases/ 95030
17 ((chronic* or persist* or recur* or ongoing*) adj2 (neck* or cervical* or shoulder* or elbow* or wrist* or hand* or thoracic* or lumbar* or low* back* or back* or spin* or hip* or knee* or ankle* or foot* or musculoskeletal* or non-cancer*) adj3 pain).mp. 27414
18 ((bone* or muscle* or muscular* or ligament* or tendon* or joint* or cartilage* or musculoskeletal*) adj2 (disorder* or injur* or pain* or condition* or syndrome* or discomfort* or strain* or sprain* or tenderness* or stiffness* or ache* or numbness* or fatigue* or soreness*)).mp. 210803
19 Arthralgia/ 10690
20 arthralgia*.mp. 20705
21 Fibromyalgia/ 10988
22 fibromyalgia*.mp. 16208
23 exp Back Pain/ 48888
24 back pain*.mp. 85331
25 Neck Pain/ 9646
26 neck pain*.mp. 19022
27 Arthritis/ 38191
28 arthrit*.mp. 282353
29 exp Osteoarthritis/ 87787
30 osteoarthrit*.mp. 134208
31 Arthritis, Rheumatoid/ 117150
32 rheumatoid arthrit*.mp. 135574
33 Spondylarthritis/ 4230
34 spondylarthrit*.mp. 5428
35 12 or 13 or 14 or 15 or 16 or 17 or 18 or 19 or 20 or 21 or 22 or 23 or 24 or 25 or 26 or 27 or 28 or 29 or 30 or 31 or 32 or 33 or 34 2462209
36 exp Randomized Controlled Trial/ 655976
37 controlled clinical trial.pt. 95759
38 random*.ab. 1657874
39 placebo.ab. 265480
40 trial.ab. 795253

41	groups.ab.	2971758
42	Systematic Review/	315672
43	systematic review.tw,pt.	423443
44	36 or 37 or 38 or 39 or 40 or 41 or 42 or 43	4840131
45	11 and 35 and 44	1383

Embase Classic <1947 to 1973> Part 1 of 2
Embase <1974 to 2026 January 07>

1 exp randomized controlled trial/1138577
2 Controlled clinical trial/ 461510
3 random$.ti,ab. 2574218
4 randomization/ 102011
5 intermethod comparison/ 318451
6 placebo.ti,ab. 477074
7 (compare or compared or comparison).ti. 715667
8 ((evaluated or evaluate or evaluating or assessed or assess) and (compare or compared or comparing or comparison)).ab. 3457243
9 (open adj label).ti,ab. 193932
10 ((double or single or doubly or singly) adj (blind or blinded or blindly)).ti,ab. 378267
11 double blind procedure/ 318214
12 parallel group$1.ti,ab. 54252
13 (crossover or cross over).ti,ab. 160274
14 ((assign$ or match or matched or allocation) adj5 (alternate or group$1 or intervention$1 or patient$1 or subject$1 or participant$1)).ti,ab. 528204
15 (assigned or allocated).ti,ab. 627552
16 (controlled adj7 (study or design or trial)).ti,ab. 655974
17 (volunteer or volunteers).ti,ab. 331598
18 human experiment/ 740556
19 trial.ti. 583017
20 or/1-19 7752573
21 (random$ adj sampl$ adj7 ("cross section$" or questionnaire$1 or survey$ or database$1)).ti,ab. not (comparative study/ or controlled study/ or randomi?ed controlled.ti,ab. or randomly assigned.ti,ab.) 10953
22 Cross-sectional study/ not (exp randomized controlled trial/ or controlled clinical study/ or controlled study/ or randomi?ed controlled.ti,ab. or control group$1.ti,ab.) 487781
23 (((case adj control$) and random$) not randomi?ed controlled).ti,ab. 25035
24 Systematic review.ti,ab. not (trial or study).ti. 443889
25 (nonrandom$ not random$).ti,ab. 21718
26 "random field$".ti,ab. 3297
27 (random cluster adj3 sampl$).ti,ab. 1815
28 (review.ab. and review.pt.) not trial.ti. 1363902
29 "we searched".ab. and (review.ti. or review.pt.) 61761
30 "update review".ab. 156
31 (databases adj4 searched).ab. 81884
32 (rat or rats or mouse or mice or swine or porcine or murine or sheep or lambs or pigs or piglets or rabbit or rabbits or cat or cats or dog or dogs or cattle or bovine or monkey or monkeys or trout or marmoset$1).ti. and animal experiment/ 1333595
33 Animal experiment/ not (human experiment/ or human/) 2826034
34 or/21-33 5108435
35 20 not 34 6822176
36 mindfulness/ 23526
37 meditation/ 12827
38 mindfulness meditation/ 2040
39 mindfulness-based stress reduction/ 1925
40 mindfulness-based cognitive therapy/ 1131
41 mindful*.mp. 37622

42 meditat*.mp. 20052
43 third wave.mp. 2595
44 mind body.mp. 7844
45 stress reduction.mp. 9322
46 vipassana.mp. 133
47 satipatthana.mp. 5
48 anapanasati.mp. 21
49 36 or 37 or 38 or 39 or 40 or 41 or 42 or 43 or 44 or 45 or 46 or 47 or 48 62728
50 exp chronic pain/ 101463
51 ((chronic* or persist* or recur* or ongoing*) adj5 pain*).mp. 246828
52 exp musculoskeletal pain/ 231422
53 (musculoskeletal* or bone* or cartilage* or joint* or muscular*).mp. 2932352
54 musculoskeletal disease/ or arthropathy/ or bone disease/ or chondropathy/ or ligament disease/ or muscle disease/ or musculoskeletal injury/ or musculoskeletal stiffness/ or tendon disease/ 146679
55 ((chronic* or persist* or recur* or ongoing*) adj2 (neck* or cervical* or shoulder* or elbow* or wrist* or hand* or thoracic* or lumbar* or low* back* or back* or spin* or hip* or knee* or ankle* or foot* or musculoskeletal* or non-cancer*) adj3 pain).mp. 42013
56 ((bone* or muscle* or muscular* or ligament* or tendon* or joint* or cartilage* or musculoskeletal*) adj2 (disorder* or injur* or pain* or condition* or syndrome* or discomfort* or strain* or sprain* or tenderness* or stiffness* or ache* or numbness* or fatigue* or soreness*)).mp. 359404
57 arthralgia/ 95400
58 arthralgia*.mp. 100434
59 fibromyalgia/ 31638
60 fibromyalgia*.mp. 33818
61 exp backache/ 165201
62 backache*.mp. 81369
63 back pain*.mp. 138404
64 neck pain/ 38672
65 neck pain*.mp. 42923
66 arthritis/ 92864
67 arthrit*.mp. 486372
68 osteoarthritis/ or hand osteoarthritis/ or hip osteoarthritis/ or knee osteoarthritis/ 191008
69 osteoarthrit*.mp. 224296
70 rheumatoid arthritis/ 256789
71 rheumatoid arthrit*.mp. 307312
72 spondylosis/ or cervical spondylosis/ or lumbar spondylosis/ or spinal osteoarthritis/ 11944
73 spondyl*.mp. 117238
74 50 or 51 or 52 or 53 or 54 or 55 or 56 or 57 or 58 or 59 or 60 or 61 or 62 or 63 or 64 or 65 or 66 or 67 or 68 or 69 or 70 or 71 or 72 or 73 3811803
75 35 and 49 and 74 2311

Web of Science

TOPIC: "mindful*" or "meditat*" or "third wave" or "mind body" or "stress reduction" or "vipassana" or "satipatthana" or "anapanasati"

AND

TOPIC: "chronic* pain" or "persist* pain" or "recur* pain" or "ongoing* pain" or "musculoskeletal* pain" or ((“chronic*” or “persist*” or “recur*” or “ongoing*”) NEAR/2 (“neck*” or “cervical*” or “shoulder*” or “elbow*” or “wrist*” or “hand*” or “thoracic*” or “lumbar*” or "low* back" or “back*” or “spin*” or “hip*” or “knee*” or “ankle*” or “foot*” or “musculoskeletal*” or "non-cancer*") NEAR/2 pain) or ((“bone*” or “muscle*” or “muscular*” or “ligament*” or “tendon*” or “joint*” or “cartilage*” or “musculoskeletal*”) NEAR/2 (“disorder*” or “injur*” or “pain*” or “condition*” or “syndrome*” or “discomfort*” or “strain*” or “sprain*” or “tenderness*” or “stiffness*” or “ache*” or “numbness*” or “fatigue*” or “soreness*”)) or “arthralgia*” or “fibromyalgia*” or “backache*” or "back pain*" or "neck pain*" or “arthrit*” or “osteoarthrit*” or “spondylosis*”

3,582 results

Date: 09/01/2026

Scopus

TITLE-ABS-KEY: "mindful*" or "meditat*" or "third wave" or "mind body" or "stress reduction" or "vipassana" or "satipatthana" or "anapanasati"

AND

TITLE-ABS-KEY: "chronic* pain" or "persist* pain" or "recur* pain" or "ongoing* pain" or "musculoskeletal* pain" or ((“chronic*” or “persist*” or “recur*” or “ongoing*”) W/2 (“neck*” or “cervical*” or “shoulder*” or “elbow*” or “wrist*” or “hand*” or “thoracic*” or “lumbar*” or "low* back" or “back*” or “spin*” or “hip*” or “knee*” or “ankle*” or “foot*” or “musculoskeletal*” or "non-cancer*") W/2 pain) or ((“bone*” or “muscle*” or “muscular*” or “ligament*” or “tendon*” or “joint*” or “cartilage*” or “musculoskeletal*”) W/2 (“disorder*” or “injur*” or “pain*” or “condition*” or “syndrome*” or “discomfort*” or “strain*” or “sprain*” or “tenderness*” or “stiffness*” or “ache*” or “numbness*” or “fatigue*” or “soreness*”)) or “arthralgia*” or “fibromyalgia*” or “backache*” or "back pain*" or "neck pain*" or “arthrit*” or “osteoarthrit*” or “spondylosis*”

4,047 results

Date: 09/01/2026

EBM Reviews - Cochrane Central Register of Controlled Trials <December 2025>

1	Mindfulness/	2674
2	Meditation/	1158
3	mindful*.mp.	13090
4	meditat*.mp.	6194
5	third wave.mp.	250
6	mind body.mp.	1978
7	stress reduction.mp.	3449
8	vipassana.mp.	28
9	satipatthana.mp.	1
10	anapanasati.mp.	6
11	1 or 2 or 3 or 4 or 5 or 6 or 7 or 8 or 9 or 10	18513
12	chronic pain/	5124
13	((chronic* or persist* or recur* or ongoing*) adj5 pain).mp.	35742
14	musculoskeletal pain/	819
15	(musculoskeletal* or bone* or cartilage* or joint* or muscular*).mp.	162572
16	musculoskeletal diseases/ or bone diseases/ or cartilage diseases/ or joint diseases/ or muscular diseases/	2862
17	((chronic* or persist* or recur* or ongoing*) adj2 (neck* or cervical* or shoulder* or elbow* or wrist* or hand* or thoracic* or lumbar* or low* back* or back* or spin* or hip* or knee* or ankle* or foot* or musculoskeletal* or non-cancer*) adj3 pain).mp.	12362
18	((bone* or muscle* or muscular* or ligament* or tendon* or joint* or cartilage* or musculoskeletal*) adj2 (disorder* or injur* or pain* or condition* or syndrome* or discomfort* or strain* or sprain* or tenderness* or stiffness* or ache* or numbness* or fatigue* or soreness*)).mp.	43372
19	Arthralgia/	1310
20	arthralgia*.mp.	6766
21	Fibromyalgia/	2138
22	fibromyalgia*.mp.	4640
23	exp back pain/	8254
24	back pain*.mp.	22090
25	neck pain/	2352
26	neck pain*.mp.	6507
27	Arthritis/	2477
28	arthrit*.mp.	32090
29	exp osteoarthritis/	11724
30	osteoarthrit*.mp.	28818
31	Arthritis, Rheumatoid/	7953
32	rheumatoid arthrit*.mp.	20516
33	Spondylarthritis/	377
34	spondylarthrit*.mp.	1126
35	12 or 13 or 14 or 15 or 16 or 17 or 18 or 19 or 20 or 21 or 22 or 23 or 24 or 25 or 26 or 27 or 28 or 29 or 30 or 31 or 32 or 33 or 34	246792
36	11 and 35	2179

CINAHL
Friday, January 9, 2026 10:36:14 AM

#	Query	Limiters/Expanders	Last Run Via	Results
S37	S12 AND S36	Expanders - Apply equivalent subjects<br>Search modes - Boolean/Phrase	Search Screen - Advanced Search<br>Database - CINAHL Complete	2,143
S36	S13 OR S14 OR S15 OR S16 OR S17 OR S18 OR S19 OR S20 OR S21 OR S22 OR S23 OR S24 OR S25 OR S26 OR S27 OR S28 OR S29 OR S30 OR S31 OR S32 OR S33 OR S34 OR S35	Expanders - Apply equivalent subjects<br>Search modes - Boolean/Phrase	Search Screen - Advanced Search<br>Database - CINAHL Complete	Display
S35	"spondyl*"	Expanders - Apply equivalent subjects<br>Search modes - Boolean/Phrase	Search Screen - Advanced Search<br>Database - CINAHL Complete	Display
S34	(MH "Spondylarthritis") OR (MH "Spondylosis") OR (MH "Spondylolysis")	Expanders - Apply equivalent subjects<br>Search modes - Boolean/Phrase	Search Screen - Advanced Search<br>Database - CINAHL Complete	Display
S33	"rheumatoid arthrit*"	Expanders - Apply equivalent subjects<br>Search modes - Boolean/Phrase	Search Screen - Advanced Search<br>Database - CINAHL Complete	Display
S32	(MH "Arthritis, Rheumatoid")	Expanders - Apply equivalent subjects<br>Search modes - Boolean/Phrase	Search Screen - Advanced Search<br>Database - CINAHL Complete	Display
S31	"osteoarthrit*"	Expanders - Apply equivalent subjects<br>Search modes - Boolean/Phrase	Search Screen - Advanced Search<br>Database - CINAHL Complete	Display
S30	(MH "Osteoarthritis+")	Expanders - Apply equivalent subjects<br>Search modes - Boolean/Phrase	Search Screen - Advanced Search<br>Database - CINAHL Complete	Display
S29	"arthrit*"	Expanders - Apply equivalent subjects<br>Search modes - Boolean/Phrase	Search Screen - Advanced Search<br>Database - CINAHL Complete	Display
S28	(MH "Arthritis")	Expanders - Apply equivalent subjects<br>Search modes - Boolean/Phrase	Search Screen - Advanced Search<br>Database - CINAHL Complete	Display
S27	"neck pain*"	Expanders - Apply equivalent subjects<br>Search modes - Boolean/Phrase	Search Screen - Advanced Search<br>Database - CINAHL Complete	Display
S26	(MH "Neck Pain")	Expanders - Apply equivalent subjects<br>Search modes - Boolean/Phrase	Search Screen - Advanced Search<br>Database - CINAHL Complete	Display

S25	"back pain*"	Expanders - Apply equivalent subjects<br>Search modes - Boolean/Phrase	Search Screen - Advanced Search<br>Database - CINAHL Complete	Display
S24	(MH "Back Pain+")	Expanders - Apply equivalent subjects<br>Search modes - Boolean/Phrase	Search Screen - Advanced Search<br>Database - CINAHL Complete	Display
S23	"fibromyalgia*"	Expanders - Apply equivalent subjects<br>Search modes - Boolean/Phrase	Search Screen - Advanced Search<br>Database - CINAHL Complete	Display
S22	(MH "Fibromyalgia")	Expanders - Apply equivalent subjects<br>Search modes - Boolean/Phrase	Search Screen - Advanced Search<br>Database - CINAHL Complete	Display
S21	"arthralgia*"	Expanders - Apply equivalent subjects<br>Search modes - Boolean/Phrase	Search Screen - Advanced Search<br>Database - CINAHL Complete	Display
S20	(MH "Arthralgia+")	Expanders - Apply equivalent subjects<br>Search modes - Boolean/Phrase	Search Screen - Advanced Search<br>Database - CINAHL Complete	Display
S19	((“bone*” OR “muscle*” OR “muscular*” OR “ligament*” OR “tendon*” OR “joint*” OR “cartilage*” OR “musculoskeletal*”) N2 (“disorder*” OR “injur*” OR “pain*” OR “condition*” OR “syndrome*” OR “discomfort*” OR “strain*” OR “sprain*” OR “tenderness*” OR “stiffness*” OR “ache*” OR “numbness*” OR “fatigue*” OR “soreness*”))	Expanders - Apply equivalent subjects<br>Search modes - Boolean/Phrase	Search Screen - Advanced Search<br>Database - CINAHL Complete	Display
S18	((“chronic*” OR “persist*” OR “recur*” OR “ongoing*”) N2 (“neck*” OR “cervical*” OR “shoulder*” OR “elbow*” OR “wrist*” OR “hand*” OR “thoracic*” OR “lumbar*” OR "low* back" OR “back*” OR “spin*” OR “hip*” OR “knee*” OR “ankle*” OR “foot*” OR “musculoskeletal*” OR "non-cancer*") N2 pain)	Expanders - Apply equivalent subjects<br>Search modes - Boolean/Phrase	Search Screen - Advanced Search<br>Database - CINAHL Complete	Display
S17	(MH "Musculoskeletal Diseases") OR (MH "Bone Diseases") OR (MH "Cartilage Diseases") OR (MH "Joint Diseases") OR (MH "Muscular Diseases")	Expanders - Apply equivalent subjects<br>Search modes - Boolean/Phrase	Search Screen - Advanced Search<br>Database - CINAHL Complete	Display
S16	("musculoskeletal*" OR "bone*" OR "cartilage*" OR "joint*" OR "muscular*")	Expanders - Apply equivalent subjects	Search Screen - Advanced Search<br>Database - CINAHL Complete	Display

		Search modes - Boolean/Phrase		
S15	(MH "Musculoskeletal Pain")	Expanders - Apply equivalent subjects<br>Search modes - Boolean/Phrase	Search Screen - Advanced Search<br>Database - CINAHL Complete	Display
S14	(("chronic*" OR "persist*" OR "recur*" OR "ongoing*") N2 pain)	Expanders - Apply equivalent subjects<br>Search modes - Boolean/Phrase	Search Screen - Advanced Search<br>Database - CINAHL Complete	Display
S13	(MH "Chronic Pain")	Expanders - Apply equivalent subjects<br>Search modes - Boolean/Phrase	Search Screen - Advanced Search<br>Database - CINAHL Complete	Display
S12	S1 OR S2 OR S3 OR S4 OR S5 OR S6 OR S7 OR S8 OR S9 OR S10 OR S11	Expanders - Apply equivalent subjects<br>Search modes - Boolean/Phrase	Search Screen - Advanced Search<br>Database - CINAHL Complete	Display
S11	"anapanasati"	Expanders - Apply equivalent subjects<br>Search modes - Boolean/Phrase	Search Screen - Advanced Search<br>Database - CINAHL Complete	Display
S10	"satipatthana"	Expanders - Apply equivalent subjects<br>Search modes - Boolean/Phrase	Search Screen - Advanced Search<br>Database - CINAHL Complete	Display
S9	"vipassana"	Expanders - Apply equivalent subjects<br>Search modes - Boolean/Phrase	Search Screen - Advanced Search<br>Database - CINAHL Complete	Display
S8	"stress reduction"	Expanders - Apply equivalent subjects<br>Search modes - Boolean/Phrase	Search Screen - Advanced Search<br>Database - CINAHL Complete	Display
S7	"mind body"	Expanders - Apply equivalent subjects<br>Search modes - Boolean/Phrase	Search Screen - Advanced Search<br>Database - CINAHL Complete	Display
S6	"third wave"	Expanders - Apply equivalent subjects<br>Search modes - Boolean/Phrase	Search Screen - Advanced Search<br>Database - CINAHL Complete	Display
S5	"meditat*"	Expanders - Apply equivalent subjects<br>Search modes - Boolean/Phrase	Search Screen - Advanced Search<br>Database - CINAHL Complete	Display
S4	"mindful*"	Expanders - Apply equivalent subjects<br>Search modes - Boolean/Phrase	Search Screen - Advanced Search<br>Database - CINAHL Complete	Display
S3	(MH "Mind Body Techniques")	Expanders - Apply equivalent subjects<br>Search modes - Boolean/Phrase	Search Screen - Advanced Search<br>Database - CINAHL Complete	Display

S2	(MH "Meditation")	Expanders - Apply equivalent subjects<br>Search modes - Boolean/Phrase	Search Screen - Advanced Search<br>Database - CINAHL Complete	Display
S1	(MH "Mindfulness")	Expanders - Apply equivalent subjects<br>Search modes - Boolean/Phrase	Search Screen - Advanced Search<br>Database - CINAHL Complete	Display

APA PsycInfo <1806 to December 2025 Week 4>

1 Mindfulness/ 14998
2 Meditation/ 6349
3 Mindfulness Meditation/ 305
4 Mindfulness-Based Interventions/ 5054
5 Mindfulness-Based Stress Reduction/ 175
6 Mindfulness-Based Cognitive Therapy/ 280
7 mindful*.mp. 30091
8 meditat*.mp. 13583
9 third wave.mp. 1445
10 mind body.mp. 7004
11 stress reduction.mp. 4571
12 vipassana.mp. 218
13 satipatthana.mp. 40
14 anapanasati.mp. 11
15 1 or 2 or 3 or 4 or 5 or 6 or 7 or 8 or 9 or 10 or 11 or 12 or 13 or 14 47320
16 Chronic Pain/ 18438
17 ((chronic* or persist* or recur* or ongoing*) adj5 pain*).mp. 34074
18 (musculoskeletal* or bone* or cartilage* or joint* or muscular*).mp. 87884
19 musculoskeletal disorders/ 4002
20 ((chronic* or persist* or recur* or ongoing*) adj2 (neck* or cervical* or shoulder* or elbow* or wrist* or hand* or thoracic* or lumbar* or low* back* or back* or spin* or hip* or knee* or ankle* or foot* or musculoskeletal* or non-cancer*) adj3 pain).mp. 5154
21 ((bone* or muscle* or muscular* or ligament* or tendon* or joint* or cartilage* or musculoskeletal*) adj2 (disorder* or disease* or injur* or pain* or condition* or syndrome* or discomfort* or strain* or sprain* or tenderness* or stiffness* or ache* or numbness* or fatigue* or soreness*)).mp.19859
22 arthralgia*.mp. 424
23 Fibromyalgia/ 2718
24 fibromyalgia*.mp. 4377
25 back pain/ 5215
26 back pain*.mp. 8157
27 neck pain*.mp. 1671
28 arthritis/ 3120
29 arthrit*.mp. 8593
30 osteoarthrit*.mp. 2900
31 rheumatoid arthritis/ 2249
32 rheumatoid arthrit*.mp. 3327
33 spondyl*.mp. 540
34 16 or 17 or 18 or 19 or 20 or 21 or 22 or 23 or 24 or 25 or 26 or 27 or 28 or 29 or 30 or 31 or 32 or 33 132690
35 15 and 34 1761

AMED (Allied and Complementary Medicine) <1985 to November 2025>

1 meditation/ 509
2 mindful*.mp. 833
3 meditat*.mp. 922
4 third wave.mp. 11
5 Mind body medicine/ 245
6 mind body.mp. 1001
7 stress reduction.mp. 264
8 vipassana.mp. 7
9 satipatthana.mp. 0
10 anapanasati.mp. 1
11 1 or 2 or 3 or 4 or 5 or 6 or 7 or 8 or 9 or 10 2538
12 Pain intractable/ 460
13 ((chronic* or persist* or recur* or ongoing*) adj5 pain*).mp. 7004
14 musculoskeletal pain/ 318
15 (musculoskeletal* or bone* or cartilage* or joint* or muscular*).mp. 42921
16 musculoskeletal disease/ or bone disease/ or cartilage disease/ or joint disease/ or muscular disease/ 5909
17 ((chronic* or persist* or recur* or ongoing*) adj2 (neck* or cervical* or shoulder* or elbow* or wrist* or hand* or thoracic* or lumbar* or low* back* or back* or spin* or hip* or knee* or ankle* or foot* or musculoskeletal* or non-cancer*) adj3 pain).mp. 3071
18 ((bone* or muscle* or muscular* or ligament* or tendon* or joint* or cartilage* or musculoskeletal*) adj2 (disorder* or injur* or pain* or condition* or syndrome* or discomfort* or strain* or sprain* or tenderness* or stiffness* or ache* or numbness* or fatigue* or soreness*)).mp. 11997
19 arthralgia/ 260
20 arthralgia*.mp. 353
21 Fibromyalgia/ 1804
22 fibromyalgia*.mp. 2014
23 exp backache/ 7202
24 back pain*.mp. 7687
25 Neck pain/ 1390
26 neck pain*.mp. 1828
27 Arthritis/ 1369
28 arthrit*.mp. 5148
29 exp osteoarthritis/ 3573
30 osteoarthrit*.mp. 4790
31 Arthritis rheumatoid/ 1923
32 rheumatoid arthrit*.mp. 2070
33 spondylitis/ or spondylitis ankylosing/ or spondylolisthesis/ 486
34 spondyl*.mp. 910
35 12 or 13 or 14 or 15 or 16 or 17 or 18 or 19 or 20 or 21 or 22 or 23 or 24 or 25 or 26 or 27 or 28 or 29 or 30 or 31 or 32 or 33 or 34 64846
36 11 and 35 267

US National Institutes of Health Ongoing Trials Register (ClinicalTrials.gov)

Basic Search Interface

Condition/disease: “chronic pain” OR “recurring pain” OR “recurrent pain” OR “ongoing pain” OR “persistent pain” OR “persisting pain” OR “musculoskeletal pain” OR “neck pain” OR “shoulder pain” OR “back pain” OR “hip pain” OR “knee pain” OR backache OR fibromyalgia OR arthritis

Intervention/treatment: mindful OR mindfulness OR meditation OR “third wave” OR “mind body” OR “stress reduction” OR vipassana OR satipatthana OR anapanasati

Study status: Completed

433 results

Date: 09/01/2026

World Health Organization (WHO) International Clinical Trials Registry Platform (ICTRP)

Advanced Search Interface

Condition: chronic pain OR recurring pain OR recurrent pain OR ongoing pain OR persistent pain OR persisting pain OR musculoskeletal pain OR neck pain OR shoulder pain OR back pain OR hip pain OR knee pain OR backache OR fibromyalgia OR arthritis OR osteoarthritis

AND

Intervention: mindful OR mindfulness OR meditation OR third wave OR mind body OR stress reduction OR vipassana OR satipatthana OR anapanasati

Without synonyms NOT selected.

Recruitment status is: ALL

324 results

Date: 09/01/2026

Australian New Zealand Clinical Trials Registry (ANZCTR)

Basic Search Interface

(“chronic pain” OR “persistent pain” OR “musculoskeletal pain”) AND (mindful OR mindfulness)

2,271 results

Date: 09/01/2026
