# Supplementary material for: The Effects of Mindfulness‐Based Programs on Pain‐Related Cognitions in Individuals With Chronic Musculoskeletal Pain: A Systematic Review and Meta‐Analysis
Source: Pain Res Manag. 2026 Nov 9;2026:6495015. doi: 10.1155/prm/6495015 (PMC13746321; doi:10.1155/prm/6495015)
Supplement: Supplementary file 1 [file PRM-2026-6495015-s001.zip › Supplemental Digital Content 2_MIND SR_v2_11022026.pdf]

Supplemental Digital Content 2: List of Reports Excluded During Full-Text Screening with Reasons for Exclusion

Abstract Only (n = 32)

1. Ashrafioun L, Stephens A, Gallegos A, Crean H, Possemato K, Pigeon W, Neureuter S. 452- Mindfulness-Based Cognitive Therapy for Pain and Suicide: A Feasibility and acceptability Study. The Journal of Pain 2025;29:105250.
2. Beaulieu M, Gaboury I, Dobkin P, Carrier N, Gervais F, Gendron F, Roberge P, Dagenais P, Roux S, Boire G. Mindfulness-Based Stress Reduction (MBSR) to Improve Patient-Related Outcomes (PROs) in Patients with Rheumatoid Arthritis in Clinical Remission but Elevated Negative PROs: A Pragmatic Pilot Study [abstract]. Arthritis & Rheumatology 2021;73:S9.
3. Benson J, Cuscina E, Morocco S, Mahoney J. Mindfulness Meditation as an Adjunctive Treatment for Chronic Pain [abstract]. Journal of the American Psychiatric Nurses Association 2017;23(4):289-318.
4. Bobrovsky H, Clarno K, Jain D, Huber S, Choo KJ. A Decentralized Trial Evaluating the Efficacy of a Multimodal Pain Treatment Mobile Application for Osteoarthritis [abstract]. Osteoarthritis and Cartilage 2022;30:S371.
5. Burgess D, Calvert C, Bangerter A, Branson M, Cross L, Evans R, Ferguson J, Friedman J, Hagel Campbell E, Haley A, Hennessy S, Kraft C, Mahaffey M, Matthias M, Meis L, Serpa J, Taylor S, Taylor B. Do Mindfulness Interventions Cause Harm? Findings From the Learning to Apply Mindfulness to Pain Study [abstract]. The Journal of Pain 2023;24(S4):38-39.
6. Burgess D, Evans R, Calvert C, Hagel Campbell E, Branson M, Allen K, Bangerter A, Bronfort G, Cross L, Ferguson J, Friedman J, Haley A, Mahaffey M, Matthias A, Meis L, Polusny M, Serpa J, Taylor S, Taylor B Results from the Learning to Apply mindfulness to Pain Study: A Pragmatic Clinical Trial [abstract]. The Journal of Pain 2024;25(S4):37.
7. Calvert C, Hagel Campbell E, Cross L, Bangeter A, Allen K, Bronfort G, Evans R, Meis L, Haley A, Matthias M, Polusny M, Taylor B, Taylor S, Ferguson J, Burgess D. 246 – Moderators of the effects of telehealth mindfulness-based interventions for chronic pain: a randomized controlled trial. The Journal of Pain 2025;29:105044.
8. Chadi N, McMahon A, Luu TM, Haley N. A Randomized Pilot Study of an Adapted Mindfulness-Based Intervention for Adolescents With Chronic Pain [abstract]. Journal of Adolescent Health 2016;58(2):S66-S67.
9. Cour P, Pedersen M, Hojsted J. Mindfulness and chronic pain: A randomized, controlled study of effect and feasibility [abstract]. European Journal of Pain Supplements 2011;5(1):259.
10. Davis M, Zautra A. On-line Training of Mindfulness-Based Emotion Regulation for Fibromyalgia: Results of a Small Randomized Trial [abstract]. Annals of Behavioral Medicine 2013;45:S95.
11. Davis M, Zautra A, Yeung E, Wolf L, Stark S, Thummala K, Nicassio P. Mindfulness, Cognitive-Behavioural, and Education Interventions for Chronic Fibromyalgia Pain: Results of a Randomized Clinical Trial [abstract]. International Journal of Behavioural Medicine 2014;21:S119.
12. Diaz K, Wolf B. Alternative therapies: yoga and mindfulness-based stress reduction as adjunctive treatment for chronic axial pain [abstract]. The Journal of Pain 2013;14(4):S90.
13. Dowd H, Hogan M, McGuire B, Sarma K. Pilot testing of an online mindfulness-based cognitive therapy intervention for a chronic pain population [abstract]. Psychology and Health 2011;26:118-119.
14. Frumer L, Harel H, Horesh D. Group therapy for fibromyalgia: a randomized controlled trial of mindfulness-based therapy versus cognitive-behavioral therapy [abstract]. Clinical and Experimental Rheumatology 2023;41(6):1370.

15. Hanley A, Lingard A. A Single-Session, Two-Hour Mindfulness Intervention Improved Chronic Pain-Related Outcomes Three Months Later [abstract]. The Journal of Pain 2023;24(4):47.
16. La Cour, P. Mindfulness and chronic pain. What is the next step? [abstract]. Journal of Psychosomatic Research 2015;78(6):608.
17. Mohammadi, F. The Effectiveness of mindfulness-based therapy on self-efficacy of chronic pain patients [abstract]. Iranian Journal of Psychiatry and Behavioral Sciences 2018;12:171.
18. Negrei C, Miclea LC, Ginghina O, Nicolescu F, Craciun B. Clinical interventions used in diminishing pain among patients with osteoarthritis: A mindfulness intervention [abstract]. Toxicology Letters 2014;229:S94.
19. Ramasawmy P, Khalid S, Petzke F, Antal A. Mindfully stimulating the brain: A randomized sham-controlled and double-blinded pilot clinical trial pairing transcranial direct current stimulation and mindfulness meditation in fibromyalgia [abstract]. Pain Practice 2022;22(1):134.
20. Rhodes P, Salmon P, Weissbecker I, Caley M, Lush E, Sephton S. The effects of mindfulness-based stress reduction on self-efficacy in fibromyalgia patients [abstract]. Annals of Behavioral Medicine 2008;35:S47.
21. Schmidt S, Grossman P, Jena S, Schwarzer B, Naumann J, Walach H. Mindfulness-based stress reduction program as an intervention in patients suffering from fibromyalgia: Results from a three-armed randomized clinical trial [abstract]. International Journal of psychology 2008;43(3):29.
22. Sephton S, Lynch E, Weissbecker I, Ho I, Salmon P. Effects of a meditation program on symptoms of illness and neuroendocrine responses in women with fibromyalgia [abstract]. Psychosomatic Medicine 2001;63(1):91-92.
23. Serrat M, Ferres S, Nieto R, Borras X, Luciano JV, Feliu-Soler A. Immune-inflammatory pathways of the FIBROWALK multicomponent program in fibromyalgia [abstract]. Clinical and Experimental Rheumatology 2024;42(6):21.
24. Shennan J, Thomas P, Tuck W, Conaglen H, Bell J. Mindfulness-based stress reduction programme for chronic pain: A pilot study. New Zealand Medical Journal 2009;122(1292):87-88.
25. Surabhi G, Uma K, Manoj K, Rima D. Impact of yoga and meditation based lifestyle intervention on quality of life and disease activity in rheumatoid arthritis [abstract]. Indian Journal of Rheumatology 2018;13(6):S93.
26. Sylvester J, Knobloch A, Hess M. Use of a self-guided mindfulness mobile application to improve pain outcomes in individuals with knee osteoarthritis [abstract]. Clinical Journal of Sport Medicine 2022;32(2):224.
27. Thompson J, Parikh N, Gavigan K, Venkatachalam S, Nowell WB. A mindfulness program dosing study to evaluate improvement in emotional distress among people with rheumatic disease [abstract]. Annals of the rheumatic diseases 2021;80(S1):169-170.
28. Trunfio F, Bruni R, Biticchi B, Corberi E, Saracino F, Marino A, Currado D, Kun L, Lamberti L, Vomero M, Navarini L, Giacomelli R. Fibromind: The use of mindfulness-based stress reduction intervention as a potential add-on therapy to the usual treatments for the management of fibromyalgia and its impact on hope levels [abstract]. Annals of the rheumatic diseases 2024;83:1300.
29. Tsiarleston G, Castellote-Caballero MY, Segura-Jimenez V. Effectiveness of exercise and mindfulness on pain pressure threshold in patients with non-specific chronic low back pain: The BACKFIT project [abstract]. Annals of the Rheumatic Diseases 2023;83:1300.
30. Turner J, Sherman K, Anderson M, Balderson B, Cook A, Cherkin D. Catastrophizing, pain self-efficacy, mindfulness, and acceptance: relationships and changes among individuals receiving CBT, MBSR, or usual care for chronic back pain [abstract]. The Journal of Pain 2015;16(4):S96.

31. Vespa A, Ottaviani M, Giuliette MV. Effects of holistic psychotherapy and mindfulness on quality of life, pain, and temperament and character in women affected by fibromyalgia [abstract]. Psychotherapy and Psychosomatics 2019;88:133.
32. Wong, S. Effect of mindfulness-based stress reduction programme on pain and quality of life in chronic pain patients: a randomised controlled clinical trial [abstract]. Hong Kong Medical Journal 2009;15(S5):13-14.

Protocol Only (n = 25)

1. Artesh University of Medical Sciences. Effect of Stress Reduction on Chronic Pain IRCT20171031037145N2: Iranian Registry of Clinical Trials, 2020.
2. Cathcart S. Mindfulness-based meditation for tension-type headache ACTRN126100001039077: Australian New Zealand Clinical Trials Registry, 2010.
3. Cepeda N, Gordon A, Paneduro D. The Effects of Mindfulness-Based Stress Reduction on Attention and Pain-related Symptoms in Chronic Pain Patients NCT01487473: ClinicalTrials.gov, 2014.
4. Cherkin D. Comparison of CAM and Conventional Mind-Body Therapies for Chronic Back Pain NCT01467843: Clinical Trials.gov, 2017.
5. Cooperman N. Pilot of Mindfulness Oriented Recovery Enhancement in Methdone Treatment NCT03894501: ClinicalTrials.gov, 2022.
6. Day M, Bindicsova I. Internet delivered mindfulness-based cognitive therapy (iMBCT) for chronic pain: A pilot randomised controlled trial ACTRN12623000833662: Australian New Zealand Clinical Trials Registry, 2024.
7. de Jong M. Effect of mindfulness on patients with rheumatoid arthritis: a controlled effect study NL-OMON28153: Overview of Medical Research in the Netherlands (OMON), 2024.
8. Feliu-Soler A, Borràs X, Peñarrubia-María MT, Rozadilla-Sacanell A, D'Amico F, Moss-Morris R, Howard MA, Fayed N, Soriano-Mas C, Puebla-Guedea M, Serrano-Blanco A, Pérez-Aranda A, Tuccillo R, Luciano JV. Cost-utility and biological underpinnings of Mindfulness-Based Stress Reduction (MBSR) versus a psychoeducational programme (FibroQoL) for fibromyalgia: a 12-month randomised controlled trial (EUDAIMON study). BMC Complementary and Alternative Medicine 2016;16(1):81.
9. Fogarty F. The effect of Mindfulness-Based Stress Reduction on pain and disease activity with people who have Rheumatoid Arthritis: Mechanisms of action ACTRN12610000485033: Australian New Zealand Clinical Trials Registry, 2010.
10. Grossman P. Mindfulness-Based Stress Reduction Program as an Intervention in Patients With Fibromyalgia NCT00106275: ClinicalTrials.gov, 2006.
11. Horesh D. Mindfulness-Based Intervention for Fibromyalgia NCT04304664: ClinicalTrials.gov, 2020.
12. Kostek M. Meditation and Exercise to Treat Chronic Back Pain (MedExT) NCT03324659: ClinicalTrials.gov, 2021.
13. Kumar D. Telehealth Exercise and Mindfulness for Pain in Osteoarthritis (TEMPO) NCT05524116: ClinicalTrials.gov, 2025.
14. Landmark T. Acceptance and Commitment Therapy With and Without Enhanced Mindfulness Training for Chronic Pain (MUST) NCT04057144: ClinicalTrials.gov, 2025.
15. Mani R, Adhia DB, Awatere S, Gray AR, Mathew J, Wilson LC, Still A, Jackson D, Hudson B, Zeidan F, Fillingim R, De Ridder D. Self-regulation training for people with knee osteoarthritis: a protocol for a feasibility randomised control trial (MiNT trial). Frontiers in Pain Research 2024;4:1-15.
16. Michalsen A. Effectiveness of mindfulness meditation for neck pain: a randomized controlled trial DRKS00000460: German Clinical Trials Register, 2012.

17. Michalsen A. Randomized controlled trial on the effectiveness of meditation in chronic low back pain DRKS00000373: German Clinical Trial Registry, 2012.
18. Moix J. Effectiveness of Cognitive Behavioral Therapy and Mindfulness Based Stress Reduction (MBSR) for Chronic Low Back Pain NCT00360802: ClinicalTrials.gov, 2009.
19. Pickworth C. MORE in the Management of Lumbosacral Radiculopathy Symptoms: A Randomized Controlled Trial NCT04818606: ClinicalTrials.gov, 2021.
20. Priore LBD, Perez VO, Briani RV, Farinelli LALB, da Silva JdCP, Martins OMG, Lopes FA, Amorim AB, Pappas E, de Azevedo FM. Effects of an online program including mindfulness, exercise therapy and patient education compared to online exercise therapy and patient education for people with Patellofemoral Pain: protocol for a randomized clinical trial. BMC Musculoskeletal Disorders 2023;24(1):372.
21. Ribeiro A. Beneficial effects of meditation on chronic low back pain U1111-1280-6860: Registro Brasileiro de Ensaios Clínicos, 2022.
22. Sharpe L. A randomized controlled trial of Cognitive Behaviour Therapy Versus Mindfulness on Pain Interference for People with Rheumatoid Arthritis ACTRN12621000997853: Australian New Zealand Clinical Trials Registry, 2021.
23. Simpson T. An Evaluation of MBSR and CBT for Veterans With Chronic Pain (IMPROVE) NCT04486066: ClinicalTrials.gov, 2025.
24. Yousefi S. Effect of mindfulness-based stress reduction and cognitive behaviour therapy in rheumatoid arthritis patients IRCT20181128041778N1: Iranian Registry of Clinical Trials, 2019.
25. Zeidan F, Ang D. The Effects of Mindfulness Meditation on Fibromyalgia-Related Pain NCT02581332: ClinicalTrials.gov, 2008.

Not a Peer-Reviewed Scientific Publication (n = 5)

1. Allred J. The Effects of a Brief Mindfulness-Based Meditation Intervention on Chronic Pain [Doctoral dissertation]. Boiling Springs, NC: Gardner-Webb University. (2016).
2. Berkel D. A Randomized Controlled Trial of a Brief Online Mindfulness Intervention for PTSD and Chronic Pain [Doctoral dissertation]. Boulder, CO: University of Colorado. (2022).
3. Curtis M. Evaluating a mindfulness meditation smartphone app among chronic pain patients [Doctoral dissertation]. San Antonio, TX: The University of Texas at San Antonio. (2020).
4. McCallion E. Mindfulness-based stress reduction and transcranial direct current stimulation as an intervention for chronic pain [Doctoral dissertation]. Albuquerque, NM: University of New Mexico. (2017).
5. Wijesinghe V. An investigation of power and pain perception in adults with chronic pain in long term care who perform mindfulness meditation [Doctoral dissertation]. New York City, NY: New York University. (2008).

Wrong Patient Population (n = 80)

1. Alemi S, Malihialzackerini S, Abolmaali Alhoseini K, Khabiri M. Comparison of the Effectiveness of Mindfulness Training and Massage-Aromatherapy on Rising Psychological Health of Elderly Women with Chronic Pain. Iranian Journal of Health Education and Health Promotion 2020;7(4):359-370.
2. Bindicsova I, Hides LM, Brain K, Hodson F, Jackson P, Jensen M, Ehde D, Day M. Internet-delivered mindfulness-based cognitive therapy (iMBCT) for chronic pain symptom management in adults: A pilot randomized controlled trial. The Journal of Pain 2025;32:105429

3. Bogosian A, Chadwick P, Windgassen S, Norton S, McCrone P, Mosweu I, Silber E, Moss-Morris R. Distress improves after mindfulness training for progressive MS: A pilot randomised trial. Multiple Sclerosis Journal 2015;21(9):1184-1194.
4. Burgess DJ, Calvert C, Bangerter A, Branson M, Cross LJS, Evans R, Ferguson JE, Friedman JK, Hagel Campbell EM, Haley AC, Hennessy S, Kraft C, Mahaffey M, Matthias MS, Meis LA, Serpa JG, Taylor SL, Taylor BC. Do mindfulness interventions cause harm? Findings from the Learning to Apply Mindfulness to Pain (LAMP) Pragmatic Clinical Trial. Pain Medicine 2024;25(S1):S68-S76.
5. Burgess DJ, Calvert C, Hagel Campbell EM, Allen KD, Bangerter A, Behrens K, Branson M, Bronfort G, Cross LJS, Evans R, Ferguson JE, Friedman JK, Haley AC, Leininger B, Mahaffey M, Matthias MS, Meis LA, Polusny MA, Serpa JG, Taylor SL, Taylor BC. Telehealth Mindfulness-Based Interventions for Chronic Pain: The LAMP Randomized Clinical Trial. JAMA Internal Medicine 2024;184(10):1163-1173.
6. Cathcart S, Galatis N, Immink M, Proeve M, Petkov J. Brief Mindfulness-Based Therapy for Chronic Tension-Type Headache: A Randomized Controlled Pilot Study. Behavioural and Cognitive Psychotherapy 2014;42(1):1-15.
7. Cathcart S, Barone V, Immink M, Proeve M. Mindfulness training does not reduce generalized hyperalgesia in chronic tension-type headache. Journal of Pain Management 2013;6(3):217-221.
8. Chadi N, McMahon A, Vadnais M, Malboeuf-Hurtubise C, Djemli A, Dobkin P, Lacroix J, Luu T, Haley N. Mindfulness-Based Intervention for Female Adolescents with Chronic Pain: A Pilot Randomized Trial. Journal of the Canadian Academy of Child and Adolescent Psychiatry 2016;25(3):159-168.
9. Chadi N, Weisbaum E, Malboeuf-Hurtubise C, Kohut SA, Viner C, Palaniyar N, Kaufman M, Locke J, Vo DX. In-person vs. eHealth Mindfulness-based Intervention for Adolescents with Chronic Illnesses: A Pilot Randomized Trial. Adolescent Psychiatry 2019;9(1):11-23.
10. Chen S, Gao X, Shi T, Zuo X, Hong C, Zhang Y, You B, Li F, Jackson T, He Y. Promising Subjective and Objective Benefits of Modified Mindfulness-Based Stress Reduction Training for Chinese Adults with Chronic Pain: A Pilot Randomized Control Study. Pain and Therapy 2023;12(6):1397-1414.
11. Cooperman NA, Hanley AW, Kline A, Garland EL. A pilot randomized clinical trial of mindfulness-oriented recovery enhancement as an adjunct to methadone treatment for people with opioid use disorder and chronic pain: Impact on illicit drug use, health, and well-being. Journal of Substance Abuse Treatment 2021;127.
12. Cooperman NA, Lu S-E, Hanley AW, Puvananayagam T, Dooley-Budsock P, Kline A, Garland EL. Telehealth Mindfulness-Oriented Recovery Enhancement vs Usual Care in Individuals With Opioid Use Disorder and Pain: A Randomized Clinical Trial. JAMA Psychiatry 2024;81(4):338-346.
13. Damico V, Milani M, Murano L. Effect of Mindfulness-Based Stress Reduction on Patients With Chronic Pain: A Randomized Controlled Trial. Pain Management Nursing 2025;26(5):510-522.
14. Davies JN, Colagiuri B, Sharpe L, Day MA. Placebo effects contribute to brief online mindfulness interventions for chronic pain: results from an online randomized sham-controlled trial. Pain 2023;164(10):2273-2284.
15. Davoudi M, Allame Z, Niya RT, Taheri AA, Ahmadi SM. The synergistic effect of vitamin D supplement and mindfulness training on pain severity, pain-related disability and neuropathy-specific quality of life dimensions in painful diabetic neuropathy: a randomized clinical trial with placebo-controlled. Journal of Diabetes & Metabolic Disorders 2021;20(1):49-58.
16. Day MA, Matthews N, Davies JN, Walker C, Bray N, Kim J, Jensen MP. Outcome Expectancies, Effects, and Mechanisms of Brief Training in Mindfulness Meditation vs. Loving-Kindness

Meditation vs a Control Condition for Pain Management: A Randomized Pilot Study. Journal of Pain & Palliative Care Pharmacotherapy 2023;38(3):206–216.
17. Day MA, Ciol MA, Ehde DM, Mendoza ME, Borckardt J, Newman A, Chan J, Friedly J, Reed D, Drever S, Burns J, Thorn B, Jensen M. The Effects of Psychological Treatments for Chronic Pain on Reducing Opioid Use in People at Risk for Opioid Misuse: A Randomized Feasibility Trial. Pain Practice 2026;26(1):e70107.
18. de Jong M, Peeters F, Gard T, Ashih H, Doorley J, Walker R, Rhoades L, Kulich RJ, Kueppenbender KD, Alpert JE, Hoge EA, Britton WB, Lazar SW, Fava M, Mischoulon D. A Randomized Controlled Pilot Study on Mindfulness-Based Cognitive Therapy for Unipolar Depression in Patients with Chronic Pain. Journal of Clinical Psychiatry 2018;79(1):26-34.
19. de Jong M, Lazar S, Hug K, Mehling W, Hölzel B, Sack A, Peeters F, Ashih H, Mischoulon D, Gard T. Effects of Mindfulness-Based Cognitive Therapy on Body Awareness in Patients with Chronic Pain and Comorbid Depression. Frontiers in Psychology 2016;7:967.
20. Deegan O, Fullen BM, Casey M-B, Segurado R, Hearty C, Doody C. Mindfulness Combined With Exercise Online (MOVE) Compared With a Self-management Guide for Adults With Chronic Pain: A Feasibility Randomized Controlled Trial. The Clinical Journal of Pain 2023;39(8):394-407.
21. Deegan O, Fullen BM, Doody CM. The effect of mindfulness combined with exercise compared with a self-management guide on measures of nervous system sensitivity in individuals with chronic pain: a pilot randomised control trial. Irish Journal of Medical Science 2025;194(3):1167-1176.
22. Deegan O, Yoshitani M, Fullen B, Gordon M, McDonagh O, Sheerin O, Murphy J, Oyebode O, Segurdo R, Hearty C, Casey MB, Doody C. Investigating the long-term impact of a programme of mindfulness combined with exercise delivered online (MOVE) on individuals living with chronic pain-an exploratory one-year follow-up of a feasibility randomised control trial. PLOS One 2025;20(9):e0323508.
23. Diller ML, Mascaro J, Haack C, Master V. Adherence Patterns and Acceptability of a Perioperative, App-Based Mindfulness Meditation Among Surgical Patients With Chronic Pain. The American Surgeon 2024;90(5):936-938.
24. Dowd H, Hogan MJ, McGuire BE, Davis MC, Sarma KM, Fish RA, Zautra AJ. Comparison of an Online Mindfulness-Based Cognitive Therapy Intervention with Online Pain Management Psychoeducation: A Randomized Controlled Study. The Clinical Journal of Pain 2015;31(6):517-527.
25. Esmer G, Blum J, Rulf J, Pier J. Mindfulness-based stress reduction for failed back surgery syndrome: a randomized controlled trial. The Journal of the American Osteopathic Association 2010;110(11):646–652.
26. Gardner-Nix J, Backman S, Barbati J, Grummitt J. Evaluating distance education of a mindfulness-based meditation programme for chronic pain management. Journal of Telemedicine and Telecare 2008;14(2):88-92.
27. Garland EL, Baker AK, Howard MO. Mindfulness-Oriented Recovery Enhancement Reduces Opioid Attentional Bias Among Prescription Opioid-Treated Chronic Pain Patients. Journal of the Society for Social Work and Research 2017;8(4):493-509.
28. Garland EL, Froeliger B, Howard MO. Neurophysiological evidence for remediation of reward processing deficits in chronic pain and opioid misuse following treatment with Mindfulness-Oriented Recovery Enhancement: exploratory ERP findings from a pilot RCT. Journal of Behavioral Medicine 2015;38(2):327-336.
29. Garland EL, Gullapalli BT, Prince KC, Hanley AW, Sanyer M, Tuomenoksa M, Rahman T. Zoom-Based Mindfulness-Oriented Recovery Enhancement Plus Just-in-Time Mindfulness Practice Triggered by Wearable Sensors for Opioid Craving and Chronic Pain. Mindfulness 2023;14(6):1329-1345.

30. Garland EL, Hanley AW, Kline A, Cooperman NA. Mindfulness-Oriented Recovery Enhancement reduces opioid craving among individuals with opioid use disorder and chronic pain in medication assisted treatment: Ecological momentary assessments from a stage 1 randomized controlled trial. Drug and Alcohol Dependence 2019;203:61-65.
31. Garland EL, Hanley AW, Nakamura Y, Barrett JW, Baker AK, Reese SE, Riquino MR, Froeliger B, Donaldson GW. Mindfulness-Oriented Recovery Enhancement vs Supportive Group Therapy for Co-occurring Opioid Misuse and Chronic Pain in Primary Care: A Randomized Clinical Trial. JAMA Internal Medicine 2022;182(4):407-417.
32. Garland EL, Hanley AW, Riquino MR, Reese SE, Baker AK, Salas K, Yack BP, Bedford CE, Bryan MA, Atchley R, Nakamura Y, Froeliger B, Howard MO. Mindfulness-oriented recovery enhancement reduces opioid misuse risk via analgesic and positive psychological mechanisms: A randomized controlled trial. Journal of consulting and clinical psychology 2019;87(10):927–940.
33. Garland EL, Hudak J, Hanley AW, Nakamura Y. Mindfulness-oriented recovery enhancement reduces opioid dose in primary care by strengthening autonomic regulation during meditation. The American psychologist 2020;75(6):840–852.
34. Garland EL, Manusov EG, Froeliger B, Kelly A, Williams JM, Howard MO. Mindfulness-oriented recovery enhancement for chronic pain and prescription opioid misuse: results from an early-stage randomized controlled trial. Journal of consulting and clinical psychology 2014;82(3):448–459.
35. Garland EL, Nakamura Y, Bryan CJ, Hanley AW, Parisi A, Froeliger B, Marchand WR, Donaldson GW. Mindfulness-Oriented Recovery Enhancement for Veterans and Military Personnel on Long-Term Opioid Therapy for Chronic Pain: A Randomized Clinical Trial. American Journal of Psychiatry 2024;181(2):125–134.
36. Garland EL, Thomas E, Howard MO. Mindfulness-oriented recovery enhancement ameliorates the impact of pain on self-reported psychological and physical function among opioid-using chronic pain patients. Journal of Pain and Symptom Management 2014;48(6):1091-1099.
37. Garland EL, Bryan MA, Priddy SE, Riquino MR, Froeliger B, Howard MO. Effects of Mindfulness-Oriented Recovery Enhancement Versus Social Support on Negative Affective Interference During Inhibitory Control Among Opioid-Treated Chronic Pain Patients: A Pilot Mechanistic Study. Annals of Behavioral Medicine 2019;53(10):865-876.
38. Garland EL, Howard MO. Mindfulness-Oriented Recovery Enhancement Reduces Pain Attentional Bias in Chronic Pain Patients. Psychotherapy and Psychosomatics 2013;82(5):311-318.
39. Hanley AW, Lingard A, Garland EL. A Single-Session, 2-Hour Version of Mindfulness-Oriented Recovery Enhancement (One MORE) Improves Chronic Pain Patients' Pain-Related Outcomes Through 3-Month Follow-Up in a Randomized Controlled Trial. Journal of Integrative and Complementary Medicine 2024;30(9):869-877.
40. Henriksson J, Wasara E, Rönnlund M. Effects of Eight-Week-Web-Based Mindfulness Training on Pain Intensity, Pain Acceptance, and Life Satisfaction in Individuals With Chronic Pain. Psychological Reports 2016;119(3):586-607.
41. Herbert MS, Fishbein JN, Tynan M, Martinez E, Lang A, Casmar P, Higdon A, Backhaus A, Golshan S, Afari N. ACTing with Mindfulness for Pain (AMP): A pilot randomized controlled trial of an integrated acceptance and commitment therapy and mindfulness meditation program. Behaviour Research and Therapy 2025;193:104854.
42. Ho EJ, Turner AP, Jensen MP, Day MA, Ehde DM, Anastas TM, Williams RM. Positive impacts of psychological pain treatments: Supplementary analyses of a randomized clinical trial. Rehabilitation psychology 2025;70(1):36–45.

43. Howarth A, Riaz M, Perkins-Porras L, Smith JG, Subramaniam J, Copland C, Hurley M, Beith I, Ussher M. Pilot randomised controlled trial of a brief mindfulness-based intervention for those with persistent pain. Journal of Behavioral Medicine 2019;42(6):999-1014.
44. Hussain N, Said ASA. Mindfulness-Based Meditation Versus Progressive Relaxation Meditation: Impact on Chronic Pain in Older Female Patients With Diabetic Neuropathy. Journal of Evidence-Based Integrative Medicine 2019;24.
45. la Cour P, Petersen M. Effects of Mindfulness Meditation on Chronic Pain: A Randomized Controlled Trial. Pain Medicine 2015;16(4):641-652.
46. Parisi A, Taylor-Swanson L, Stewart JL, Khalsa SS, Garland EL. Enhancing interoceptive awareness in chronic pain and opioid misuse via mindfulness-oriented recovery enhancement. Drug and Alcohol Dependence 2025;274:112741.
47. Sarrami Foroushani N, Mohammadkhani P, Rasti J. The Effectiveness of Mindfulness-Based Cognitive Therapy on the Symptoms of Pain Perception in Older Women. Salmand: Iranian Journal of Ageing 2024;19(2):242-257.
48. Law H, Avins A, Stahl R, Goodreau M, Jacobson A, Sudat S, Pressman A. Recruitment, retention, and adherence in a randomized feasibility trial of mindfulness-based stress reduction for patients with migraine. Complementary Therapies in Medicine 2020;55:102610.
49. Liebmann C, Konrad AC, Singer T, Kanske P. Differential reduction of psychological distress by three different types of meditation-based mental training programs: A randomized clinical trial. International Journal of Clinical and Health Psychology 2023;23(4):100388.
50. Majore-Dusele I, Karkou V, Millere I. The Development of Mindful-Based Dance Movement Therapy Intervention for Chronic Pain: A Pilot Study With Chronic Headache Patients. Frontiers in Psychology 2021;12.
51. Mansourishad H, Togha M, Borjali A, Karimi R. Effectiveness of Mindfulness-Based Cognitive-Behavioral Therapy on Relieving Migraine Headaches. Archives of Neuroscience 2017;4(4):e58028.
52. Mascaro JS, Singh V, Wehrmeyer K, Scott B, Juan J, McKenzie-Brown AM, Lane OP, Haack C. Randomized, wait-list–controlled pilot study of app-delivered mindfulness for patients reporting chronic pain. PAIN Reports 2021;6(1):e924.
53. Mawani A-N, Rashiq S, Verrier M, Dick B. The effect of mindfulness meditation on pain and spirituality in patients with chronic non-cancer pain. Journal of Pain Management 2014;7(1):75-82.
54. McCallion E, Robinson CSH, Clark VP, Witkiewitz K. Efficacy of Transcranial Direct Current Stimulation-Enhanced Mindfulness-Based Program for Chronic Pain: a Single-Blind Randomized Sham Controlled Pilot Study. Mindfulness 2020;11(4):895-904.
55. Morledge TJ, Allexandre D, Fox E, Fu AZ, Higashi MK, Kruzikas DT, Pham SV, Reese PR. Feasibility of an Online Mindfulness Program for Stress Management—A Randomized, Controlled Trial. Annals of Behavioral Medicine 2013;46(2):137-148.
56. Namjoo S, Borjali A, Seirafi M, Assarzadegan F. Use of Mindfulness-based cognitive therapy to change pain-related cognitive processing in patients with primary headache: A randomized trial with attention placebo control group. Anesthesiology and Pain Medicine 2019;9(5):e91927.
57. Omidi A, Zargar F. Effects of mindfulness-based stress reduction on perceived stress and psychological health in patients with tension headache. Journal of Research in Medical Sciences 2015;20(11):1058-1063.
58. Omidi A, Zargar F. Effect of mindfulness-based stress reduction on pain severity and mindful awareness in patients with tension headache: a randomized controlled clinical trial. Nursing and Midwifery Studies 2014;3(3):e21136.
59. Pardos-Gascón EM, Narambuena L, Leal-Costa C, Ramos-Morcillo AJ, Ruzafa-Martínez M, van-der Hofstadt Román CJ. Effects of Mindfulness-Based Cognitive Therapy for Chronic

Pain: A Multicenter Study. International Journal of Environmental Research and Public Health 2021;18(13):6951.
60. Parisi A, Hudak J, Froeliger B, Garland EL. Mindfulness-Oriented Recovery Enhancement reduces post-traumatic stress via reappraisal among patients with chronic pain and co-occurring opioid misuse. Nature Mental Health 2023;1(7):489-500.
61. Parisi A, Hanley AW, Garland EL. Mindfulness-Oriented Recovery Enhancement reduces opioid craving, pain, and negative affect among chronic pain patients on long-term opioid therapy: An analysis of within- and between-person state effects. Behaviour Research and Therapy 2022;152:104066.
62. Pérez-Fernández JI, Salaberria K, Ruiz de Ocenda Á. Mindfulness-Based Pain Management (MBPM) for Chronic Pain: a Randomized Clinical Trial. Mindfulness 2022;13(12):3153-3165.
63. Price C, Pike K, Treadway A, Palmer J, Merrill J. Immediate effects of mindful awareness in body-oriented therapy as an adjunct to medication for opioid use disorder [Preprint]. 2024.
64. Reiner K, Granot M, Soffer E, Lipsitz JD. A Brief Mindfulness Meditation Training Increases Pain Threshold and Accelerates Modulation of Response to Tonic Pain in an Experimental Study. Pain Medicine 2015;17(4):628-635.
65. Rice VJB, Schroeder P, Allison SC. Effects of mindfulness meditation training offered in-person and via a virtual world on self-compassion: A study with U.S. military active duty and veterans. Work 2024;78(4):1225-1245.
66. Roberts RL, Ledermann K, Garland EL. Mindfulness-oriented recovery enhancement improves negative emotion regulation among opioid-treated chronic pain patients by increasing interoceptive awareness. Journal of Psychosomatic Research 2022;152:110677.
67. Rod, K. Observing the effects of mindfulness-based meditation on anxiety and depression in chronic pain patients. Psychiatria Danubina 2015;27(S1):209-211.
68. Roll SC, Hardison ME, Vigen C, Black DS. Mindful Body Scans and Sonographic Biofeedback as Preparatory Activities to Address Patient Psychological States in Hand Therapy: A Pilot Study. Hand Therapy 2020;25(3):98-106.
69. Rozworska KA, Poulin PA, Carson A, Tasca GA, Nathan HJ. Mediators and moderators of change in mindfulness-based stress reduction for painful diabetic peripheral neuropathy. Journal of Behavioral Medicine 2020;43(2):297-307.
70. Ruskin DA, Gagnon MM, Kohut SA, Stinson JN, Walker KS. A Mindfulness Program Adapted for Adolescents With Chronic Pain: Feasibility, Acceptability, and Initial Outcomes. The Clinical Journal of Pain 2017;33(11):1019-1029.
71. Sampalli T, Berlasso E, Fox R, Petter M. A controlled study of the effect of a mindfulness-based stress reduction technique in women with multiple chemical sensitivity, chronic fatigue syndrome, and fibromyalgia. Journal of Multidisciplinary Healthcare 2009;2:53-59.
72. Sheybani F, Dabaghi P, Najafi S, Rajaeinejad M. Effectiveness of mindfulness-based stress reduction (MBSR) on patients with chronic pain: A randomized clinical trial. Iranian Journal of Psychiatry and Clinical Psychology 2022;28(2):182-195.
73. Torrijos-Zarcero M, Mediavilla R, Rodríguez-Vega B, Del Río-Diéguez M, López-Álvarez I, Rocamora-González C, Palao-Tarrero A. Mindful Self-Compassion program for chronic pain patients: A randomized controlled trial. European Journal of Pain 2021;25:930-944.
74. Turner AP, Edwards KA, Jensen MP, Ehde DM, Day MA, Williams RM. Effects of hypnosis, mindfulness meditation, and education for chronic pain on substance use in veterans: A supplementary analysis of a randomized clinical trial. Rehabilitation Psychology 2023;68(3):261-270.
75. Ussher M, Spatz A, Copland C, Nicolaou A, Cargill A, Amini-Tabrizi N, McCracken LM. Immediate effects of a brief mindfulness-based body scan on patients with chronic pain. Journal of Behavioral Medicine 2014;37(1):127-134.

76. Verdú García ML, Quiles Marcos Y. Application of the Mindfulness-Based Stress Reduction program to patients with borderline personality disorder and chronic pain: A pilot study. Behavioral Psychology 2022;30(2):373-390.
77. Vo DX, Tabi K, Bhullar M, Johnson A, Locke J, Wang S, Stewart SE, Marshall SK. Mindful awareness and resilience skills for adolescents (MARS-A): a mixed-methods study of a mindfulness-based intervention for a heterogeneous adolescent clinical population. International Journal of Adolescent Medicine and Health 2024;36(1):25-35.
78. Westenberg RF, Zale EL, Heinhuis TJ, Özkan S, Nazzal A, Lee S-G, Chen NC, Vranceanu A-M. Does a Brief Mindfulness Exercise Improve Outcomes in Upper Extremity Patients? A Randomized Controlled Trial. Clinical Orthopaedics and Related Research 2018;476(4):790-798.
79. Williams RM, Day MA, Ehde DM, Turner AP, Ciol MA, Gertz KJ, Patterson D, Hakimian S, Suri P, Jensen MP. Effects of hypnosis vs mindfulness meditation vs education on chronic pain intensity and secondary outcomes in veterans: a randomized clinical trial. Pain 2022;163(10):1905-1918.
80. Zhou J, Ping P, and Xie X. Prevalence of Pain and Effects of a Brief Mindfulness-Based Intervention on Chinese Community-Dwelling Older Adults with Chronic Pain. Journal of Community Health Nursing 2018;35(1):19-27.

Wrong Intervention (n = 43)

1. Ahn H, Zhong C, Miao H, Chaoul A, Park L, Yen IH, Vila MA, Sorkpor S, Abdi S. Efficacy of combining home-based transcranial direct current stimulation with mindfulness-based meditation for pain in older adults with knee osteoarthritis: A randomized controlled pilot study. Journal of Clinical Neuroscience 2019;70:140-145.
2. Alemi S, Abolmaali Alhosseini K, Malihialzackerini S, Khabiri M. Effect of Mindfulness Therapy and Aromatherapy Massage on Pain Perception, Quality of Life and Sleep Quality in Older Women With Chronic Pain. Salmand: Iranian Journal of Ageing 2021;16(2):218-233.
3. Amiot L, Daneau C, Couëpel B, Descarreaux M, Bernier M, Fournier J, Ruffault A, Courbalay A. Effects of an acute Mindfulness-based intervention on exercise tolerance, maximal strength, pain and effort-related experiences in individuals with primary chronic low back pain: a pilot study. Movement and Sports Sciences - Science et Motricite 2023;120:41-54.
4. Arey DL, Gerbi A, Sagi A. A randomized controlled trial on single-session mindfulness self-compassion interventions for Fibromyalgia Syndrome: evaluating interoceptive awareness, anxiety, and pain. Current Psychology 2024;43(47):36234-36245.
5. Buranruk O. A randomized clinical trial of self-stretching with and without mindful breathing - immediate effect on pressure pain and range of motion in myofascial pain syndrome. Journal of Bodywork and Movement Therapies 2022;32:29-35.
6. Choi H, Hahm SC, Jeon YH, Han JW, Kim SY, Woo JM. The Effects of Mindfulness-Based Mandala Coloring, Made in Nature, on Chronic Widespread Musculoskeletal Pain: Randomized Trial. Healthcare 2021;9(6):642.
7. El Mabrouk Y, Ben Ammou A, Akremi S, Ben Abid A, Ben Salah M. AB1587 How does Ho'oponopono meditation affect quality of sleep in chronic musculoskeletal pain patients? [abstract]. Annals of the Rheumatic Diseases 2024;83:2168.
8. Ebnezar J, Nagarathna R, Bali Y, Nagendra HR. Effect of an integrated approach of yoga therapy on quality of life in osteoarthritis of the knee joint: A randomized control study. International journal of yoga 2011;4(2):55-63.
9. Finan PH, Hunt C, Keaser ML, Smith K, Lerman S, Bingham CO, Barrett F, Garland EL, Zeidan F, Seminowicz DA. Effects of Savoring Meditation on Positive Emotions and Pain-Related Brain Function: A Mechanistic Randomized Controlled Trial in People With Rheumatoid Arthritis. The Journal of Pain 2024;25(7).

10. Hanley AW, Nakamura Y, Edwards RR, Lingard A, Childs K, Faulkner J, Schnitt R, Vaca-St Claire S, Bridge E, Garldan E. Complementary therapies for chronic knee pain: A placebo-controlled RCT of Reiki and mindfulness. Complementary Therapies in Medicine 2025;95:103278.
11. Haugmark T, Hagen KB, Provan SA, Smedslund G, Zangi HA. Effects of a mindfulness-based and acceptance-based group programme followed by physical activity for patients with fibromyalgia: a randomised controlled trial. BMJ Open 2021;11(6):e046943.
12. Hodren N, Williamson T, Shires a. Effectiveness of the Brief Self-Implemented Mindfulness-Based Interoceptive Exposure Task Taught Online in a Mixed Chronic Pain Group. Mindfulness 2025;16:3518-3534.
13. Hopkins SW, Greenberg J, Isaacs J, Vranceanu A-M. "Practice Makes Perfect"? Associations Between Home Practice and Physical and Emotional Function Outcomes Among Patients with Chronic Pain Enrolled in a Mind–Body Program. Journal of Integrative and Complementary Medicine 2022;28(4):320-327.
14. Hsu MC, Schubiner H, Lumley MA, Stracks JS, Clauw DJ, Williams DA. Sustained Pain Reduction Through Affective Self-awareness in Fibromyalgia: A Randomized Controlled Trial. Journal of General Internal Medicine 2010;25(10):1064-1070.
15. Igna R, Stefan S, Onac I, Ungar R, Szentagotai Tatar A. Mindfulness-based cognitive behavior therapy (MCBT) versus virtual reality (VR) enhanced CBT, versus treatment as usual for chronic back pain: a clinical trial. Journal of Evidence-Based Psychotherapies 2014;14(2):229-247.
16. Innes KE, Selfe TK, Kandati S, Wen S, Huysmans Z. Effects of Mantra Meditation versus Music Listening on Knee Pain, Function, and Related Outcomes in Older Adults with Knee Osteoarthritis: An Exploratory Randomized Clinical Trial (RCT). Evidence-Based Complementary and Alternative Medicine 2018;(1):7683897.
17. Jeitler M, Brunnhuber S, Meier L, Lüdtke R, Büssing A, Kessler C, Michalsen A. Effectiveness of Jyoti Meditation for Patients With Chronic Neck Pain and Psychological Distress: A Randomized Controlled Clinical Trial. The Journal of Pain 2015;16(1):77-86.
18. Khatib L, Dean JG, Oliva V, Riegner G, Gonzalez NE, Birenbaum J, Cruanes GF, Miller J, Patterson M, Kim H-C, Chakravarthy K, Zeidan F. The role of endogenous opioids in mindfulness and sham mindfulness-meditation for the direct alleviation of evoked chronic low back pain: a randomized clinical trial. Neuropsychopharmacology 2024;49(7):1069-1077.
19. Kulkarni P, Welling A, Gurudut P, Kage V. Impact of Virtual Reality and Walking Meditation on Proprioception and Balance in Individuals With Knee Osteoarthritis: A Randomized Clinical Trial. Alternative Therapies in Health and Medicine [online preprint] 2025.
20. Kuntz AB, Chopp-Hurley JN, Brenneman EC, Karampatos S, Wiebenga EG, Adachi JD, Noseworthy MD, Maly MR. Efficacy of a biomechanically-based yoga exercise program in knee osteoarthritis: A randomized controlled trial. PLOS One 2018;13(4):e0195653.
21. Liu Y, Shang J, Zhang Y, Liu L, Zhang W. A clinical study of ultrasound-guided acupotomy combined with mindfulness meditation to improve lumbar myofascial pain syndrome. Frontiers in Neurology 2025;2(16):1627376.
22. Lyu F, Ramoo V, Chui P. Effects of Tailored Mindful Exercise on Back Pain, Balance, Kinesiophobia, Anxiety and Depression in Older Patients with Primary Osteoporosis: A Randomized Controlled Trial. Mindfulness 2025;16:2808-2824.
23. Maddali Bongi S, Di Felice C, Del Rosso A, Galluccio F, Landi G, Tai G, Giambalvo Dal Ben G, Matucci Cerinic M. The efficacy of the Rességuier method in the treatment of fibromyalgia syndrome: a randomized controlled trial. Clinical and Experimental Rheumatology 2010;28(6):S46-50.
24. McGeary DD, Jaramillo C, Eapen B, Blount TH, Nabity PS, Moreno J, Pugh MJ, Houle TT, Potter JS, Young-McCaughan S, Peterson AL, Villarreal R, Brackins N, Sikorski Z, Johnson TR, Tapia R, Reed D, Caya CA, Bomer D, Simmonds M, McGeary CA. Mindfulness-Based Interdisciplinary Pain Management Program for Complex Polymorbid Pain in Veterans: A

Randomized Controlled Trial. Archives of Physical Medicine and Rehabilitation 2022;103(10):1899-1907.
25. Michalsen A, Kunz N, Jeitler M, Brunnhuber S, Meier L, Lüdtke R, Büssing A, Kessler C. Effectiveness of focused meditation for patients with chronic low back pain: A randomized controlled clinical trial. Complementary Therapies in Medicine 2016;26:79-84.
26. Montero-Marín J, Navarro-Gil M, Puebla-Guedea M, Luciano JV, Van Gordon W, Shonin E, García-Campayo J. Efficacy of "Attachment-Based Compassion Therapy" in the Treatment of Fibromyalgia: A Randomized Controlled Trial. Frontiers in Psychiatry 2018;8:307.
27. Morcillo-Muñoz Y, Sánchez-Guarnido AJ, Calzón-Fernández S, Baena-Parejo I. Multimodal Chronic Pain Therapy for Adults via Smartphone: Randomized Controlled Clinical Trial. Journal of medical internet research 2022;24(5):e36114.
28. Provan S, Calogiuri G, Røset L, Mariussen M, Rosøy I, Johnsen TJ, Johansen T, Einar Flaten O, Litleskare S. VR-guided exercise and mindfulness program for people with chronic pain: a randomised controlled cross-over pilot trial. BMC Sports Science, Medicine and Rehabilitation 2025;17(55).
29. Olsen AL, Magnussen LH, Skjaerven LH, Assmus J, Sundal M-A, Furnes O, Hallan G, Strand LI. Basic Body Awareness Therapy versus standard care in hip osteoarthritis. A randomized controlled trial. Physiotherapy Research International 2022;27(1):e1930.
30. Paccione CE, Stubhaug A, Diep LM, Rosseland LA, Jacobsen HB. Meditative-based diaphragmatic breathing vs. vagus nerve stimulation in the treatment of fibromyalgia—A randomized controlled trial: Body vs. machine. Frontiers in Neurology 2022;13(3).
31. Pach D, Blödt S, Wang J, Keller T, Bergmann B, Rogge AA, Barth J, Icke K, Roll S, Witt CM. App-Based Relaxation Exercises for Patients With Chronic Neck Pain: Pragmatic Randomized Trial. JMIR Mhealth Uhealth 2022;10(1):e31482.
32. Polaski AM, Phelps AL, Smith TJ, Helm ER, Morone NE, Szucs KA, Kostek MC, Kolber BJ. Integrated Meditation and Exercise Therapy: A Randomized Controlled Pilot of a Combined Nonpharmacological Intervention Focused on Reducing Disability and Pain in Patients with Chronic Low Back Pain. Pain Medicine 2021;22(2):444-458.
33. Ramasawmy P, Khalid S, Petzke F, Antal A. Pain reduction in fibromyalgia syndrome through pairing transcranial direct current stimulation and mindfulness meditation: A randomized, double-blinded, sham-controlled pilot clinical trial. Frontiers in Medicine 2022;9.
34. Rotter G, Ortiz M, Binting S, Tomzik J, Reese F, Roll S, Brinkhaus B, Teut M. Mindful Walking in Patients with Chronic Low Back Pain: A Randomized Controlled Trial. Journal of Integrative and Complementary Medicine 2022;28(6):474-483.
35. Sanabria-Mazo JP, Montero-Marin J, Feliu-Soler A, Gasión V, Navarro-Gil M, Morillo-Sarto H, Colomer-Carbonell A, Borràs X, Tops M, Luciano JV, García-Campayo J. Mindfulness-Based Program Plus Amygdala and Insula Retraining (MAIR) for the Treatment of Women with Fibromyalgia: A Pilot Randomized Controlled Trial. Journal of Clinical Medicine 2020;9(10):3246.
36. Serrat M, Albajes K, Navarrete J, Almirall M, Lluch Girbés E, Neblett R, Luciano JV, Moix J, Feliu-Soler A. Effectiveness of two video-based multicomponent treatments for fibromyalgia: The added value of cognitive restructuring and mindfulness in a three-arm randomised controlled trial. Behaviour Research and Therapy 2022;158:104188.
37. Serrat M, Sanabria-Mazo JP, Almirall M, Musté M, Feliu-Soler A, Méndez-Ulrich JL, Sanz A, Luciano JV. Effectiveness of a Multicomponent Treatment Based on Pain Neuroscience Education, Therapeutic Exercise, Cognitive Behavioral Therapy, and Mindfulness in Patients With Fibromyalgia (FIBROWALK Study): A Randomized Controlled Trial. Physical Therapy 2021;101(12).
38. Sharma M, Singh A, Dhillon MS, Kaur S. Comparative Impact of Nonpharmacological Interventions on Pain of Knee Osteoarthritis Patients Reporting at a Tertiary Care Institution: A Randomized Controlled Trial. Indian Journal of Palliative Care 2018;24(4):478-485.

39. Stuve LL, Liu H, Shen J, Gianettoni J, Galipo J. Evaluation of body talk, a novel mind-body medicine for chronic pain treatment. Journal of Pain Management 2015;7(4):279-290.
40. Tahran Ö, Ersoz Huseyinsinoglu B, Yolcu G, Karadağ Saygı E, Yeldan I. Comparing face-to-face and internet-based basic body awareness therapy for fibromyalgia: a randomized controlled trial [Advance online publication]. Disability and Rehabilitation 2025:1-12.
41. Wetherell JL, Afari N, Rutledge T, Sorrell JT, Stoddard JA, Petkus AJ, Solomon BC, Lehman DH, Liu L, Lang AJ, Atkinson HJ. A randomized, controlled trial of acceptance and commitment therapy and cognitive-behavioral therapy for chronic pain. Pain 2011;152(9):2098-2107.
42. Ye J, Zheng Q, Zou L, Yu Q, Veronese N, Grabovac I, Stefanac S, Tzeng H-M, Yu JJ. Mindful Exercise (Baduanjin) as an Adjuvant Treatment for Older Adults (60 Years Old and Over) of Knee Osteoarthritis: A Randomized Controlled Trial. Evidence-Based Complementary and Alternative Medicine 2020;2020(1):9869161.
43. Zheng F, Zheng Y, Liu S, Yang J, Xiao W, Xiao W, Chen L, Yang W, Zhang S, Yu Q, Hao Z, Wang Y, Wang C. The Effect of M-Health-Based Core Stability Exercise Combined with Self-Compassion Training for Patients with Nonspecific Chronic Low Back Pain: A Randomized Controlled Pilot Study. Pain and Therapy 2022;11(2):511-528.

Wrong Outcomes (n = 5)

1. Alkasir E, Masjedi-Arani A, Jafarian Dehkordi F, Sam Kianimoghadam A, Afshari B, Yousefi S. Comparison of the effectiveness of mindfulness-based stress reduction versus cognitive-behavioral therapy on emotion regulation, mindfulness, and cognitive-executive functions in female patients with rheumatoid arthritis. Journal of Education and Health Promotion 2025;14:99.
2. Amutio A, Clemente F, Sánchez-Sánchez LC, Pérez-Fuentes M, Gázquez-Linares J, Van Gordon W, Molero-Jurado M. Effects of Mindfulness Training on Sleep Problems in Patients with Fibromyalgia. Frontiers in Psychology 2018;9:1365.
3. Hesari N, Asgari P, Naderi F, Heidari A. Effect of Positive Mindfulness and Emotion-Focused Cognitive Behavioral Therapy in Fibromyalgia Patients' Response to Stress and Subjective Well-Being. Advances in Nursing and Midwifery 2021;30(4):1-7.
4. Sharpe L, Menzies RE, Boyse J, Bisby MA, Richmond B, Todd J, Sesel A-L, Dear BF. Mediators and Moderators of two online interventions for managing pain, fear of progression and functional ability in rheumatoid arthritis. Behaviour Research and Therapy 2025;185:104676.
5. Yousefi S, Zanjani Z, Omidi A, Zamani B, Sayyah M. Comparison of Mindfulness-Based Stress Reduction Therapy and Cognitive-Behavioral Therapy of Chronic Fatigue, Sleep Quality, Executive Function and Disease Activity in Patients with Rheumatoid Arthritis. Journal of Contemporary Psychotherapy 2023;53(2):173-180.

Wrong Study Design (n = 22)

1. Anastas T, Turner A, Ho E, Day M, Ehde D, Jensen M, Williams R. Evaluating the benefits of a second pain treatment following a clinical trial. Rehabilitation psychology 2024;69(1):74–83.
2. Ardito RB, Pirro PS, Re TS, Bonapace I, Menardo V, Bruno E, Gianotti L. Mindfulness-Based Stress Reduction Program on Chronic Low-Back Pain: A Study Investigating the Impact on Endocrine, Physical, and Psychologic Functioning. The Journal of Alternative and Complementary Medicine 2017;23(8):615-623.
3. Aygün O, Mohr E, Duff C, Matthew S, Schoenberg P. Oxytocin Modulation in Mindfulness-Based Pain Management for Chronic Pain. Life 2024;14(2):253.
4. Azarnik M. The Effectiveness of Mindfulness-Based Cognitive Therapy on Quality of Life in Relation to Health and Self-efficacy in Patients with Rheumatoid Arthritis. International Journal of Body, Mind and Culture 2019;6(1):21–26.

5. Braden BB, Pipe TB, Smith R, Glaspy TK, Deatherage BR, Baxter LC. Brain and behavior changes associated with an abbreviated 4-week mindfulness-based stress reduction course in back pain patients. Brain and Behavior 2016;6(3):e00443.
6. Brandel MG, Lin C, Hennel D, Khazen O, Pilitsis JG, Ben-Haim S. Mindfulness Meditation in the Treatment of Chronic Pain. Neurosurgery Clinics of North America 2022;33(3):275-279.
7. Brintz C, Faurot K, Rai S, Gaylord S. Feasibility of a 4-week adapted mindfulness program for adults with chronic pain [abstract]. Annals of Behavioral Medicine 2019;53:S98.
8. Brintz CE, Roth I, Faurot K, Rao S, Gaylord SA. Feasibility and Acceptability of an Abbreviated, Four-Week Mindfulness Program for Chronic Pain Management. Pain Medicine 2020;21(11):2799-2810.
9. Bruckstein D. Effects of acceptance-based and cognitive behavioral interventions on chronic pain management [Doctoral dissertation]. Hempstead, NY: Hofstra University. (1999).
10. Chen S-P, Liu H-T, Appelt JC, Klassen BL, Liu L, Smith JL, Miguel-Cruz A. Feasibility of Mindfulness-Based Intervention for Veterans Managing Chronic Pain. Canadian Journal of Occupational Therapy 2023;90(3):303-314.
11. Day MA, Thorn BE, Ehde DM, Burns JW, Barnier A, Mattingley JB, Matthews N, Jensen MP. Moderators of Mindfulness Meditation, Cognitive Therapy, and Mindfulness-Based Cognitive Therapy for Chronic Low Back Pain: A Test of the Limit, Activate, and Enhance Model. The Journal of Pain 2020;21(1):161-169.
12. Gaboury I, Dobkin PL, Gendron F, Roberge P, Beaulieu M-C, Carrier N, Dagenais P, Roux S, Boire G. Mindfulness-based stress reduction to improve depression, pain and high patient global assessment in controlled rheumatoid arthritis. Rheumatology Advances in Practice 2022;6(3).
13. Gardner-Nix J, Barbati J, Grummitt J, Pukal S, Raponi Newton R. Exploring the Effectiveness of a Mindfulness-Based Chronic Pain Management Course Delivered Simultaneously to On-Site and Off-Site Patients Using Telemedicine. Mindfulness 2014;5(3):223-231.
14. Garland EL, Bryan CJ, Finan PH, Thomas EA, Priddy SE, Riquino MR, Howard MO. Pain, hedonic regulation, and opioid misuse: Modulation of momentary experience by Mindfulness-Oriented Recovery Enhancement in opioid-treated chronic pain patients. Drug and Alcohol Dependence 2017;173:S65-S72.
15. Kaplan KH, Goldenberg DL, Galvin-Nadeau M. The impact of a meditation-based stress reduction program on fibromyalgia. General Hospital Psychiatry 1993;15(5):284-289.
16. Lattimore P, and Harrison F. Pilot study of an online-delivered mindfulness meditation in Ehlers-Danlos syndrome (hEDS): effect on quality-of-life and participant lived experience. Disability and Rehabilitation 2023;45(23):3833-3840.
17. Lopes SA, Vannucchi BP, Demarzo M, Cunha ÂGJ, Nunes MdPT. Effectiveness of a Mindfulness-Based Intervention in the Management of Musculoskeletal Pain in Nursing Workers. Pain Management Nursing 2019;20(1):32-38.
18. Mohr E, Matthew S, Narisetti L, Duff C, Schoenberg P. Cognitive mechanisms of mindfulness-based pain management in chronic pain. Social Sciences & Humanities Open 2025;11:101261.
19. Pester BD, Yamin JB, Cabrera MJ, Mehta S, Silverman J, Grossestreuer AV, Howard P, Edwards RR, Donnino MW. Change in Pain-Related Anxiety Mediates the Effects of Psychophysiologic Symptom Relief Therapy (PSRT) on Pain Disability for Chronic Back Pain: Secondary Results from a Randomized Controlled Trial. Journal of Pain Research 2023;16:3871-3880.
20. Quintana M, Rincón Fernández, ME. Eficacia del entrenamiento en mindfulness para pacientes con fibromyalgia [Efficacy of mindfulness training for fibromyalgia patients]. Journal of Empirical Research in Psychology 2011;22(1):51-67.
21. Sagula D, Rice KG. The Effectiveness of Mindfulness Training on the Grieving Process and Emotional Well-Being of Chronic Pain Patients. Journal of Clinical Psychology in Medical Settings 2004;11(4):333-342.

22. Vago DR, Nakamura Y. Selective Attentional Bias Towards Pain-Related Threat in Fibromyalgia: Preliminary Evidence for Effects of Mindfulness Meditation Training. Cognitive Therapy and Research 2011;35(6):581-594.
