# Supplementary material for: The Effects of Mindfulness‐Based Programs on Pain‐Related Cognitions in Individuals With Chronic Musculoskeletal Pain: A Systematic Review and Meta‐Analysis
Source: Pain Res Manag. 2026 Nov 9;2026:6495015. doi: 10.1155/prm/6495015 (PMC13746321; doi:10.1155/prm/6495015)
Supplement: Supplementary file 1 [file PRM-2026-6495015-s001.zip › Supplemental Digital Content 3_MIND SR_v5_13032026.pdf]

Supplemental Digital Content 3: Summary of the Characteristics of the Included Studies

Study	Methods	Participants	Comparator(s)	Outcomes	Sources of Funding or Other Material Support	Competing Interests of Study Authors
Akbarzadeh et al., 2022 [a]	**Study design:** Individually RCT<br><br>**Number of study arms:** 3<br>Note: Only the MBCT and sham tDCS groups met the eligibility criteria for this review.	**Country:** Iran<br>**Number of Participants:** 24<br>**Number Analyzed:** 24<br>**Age (years):** Not reported<br>**Sex:** 70.8% female<br>**Condition:** Fibromyalgia<br>**Source of Recruitment:** Specialist rheumatology clinic in Tehran	Sham tDCS: Control group participants underwent eight sham tDCS sessions over the course of three weeks.	**Anxiety:** PSWQ at 8 weeks<br><br>**Pain Intensity:** SF-MPQ at 8 weeks<br><br>**Intervention Adherence:** See Table 1	None	None
Amutio et al., 2015	**Study design:** Individually RCT<br><br>**Number of study arms:** 2	**Country:** Spain<br>**Number of Participants:** 39<br>**Number Analyzed:** 39<br>**Age (years):** Mean 51.82 (SD 10.18)<br>**Sex:** 100% female<br>**Condition:** Fibromyalgia<br>**Source of Recruitment:** Fibromyalgia Association of Almeria	Waitlist Control: Participants in the control group were informed that they would receive the mindfulness intervention program at a later time.	**Depression:** BDI at 7 weeks<br><br>**Anxiety:** STAI – trait anxiety subscale at 7 weeks<br><br>**Intervention Adherence:** See Table 1	Not reported	The authors declare that the research was conducted in the absence of any commercial or financial relationships that could be construed as a potential conflict of interest.
Amutio et al., 2022	**Study design:** Individually RCT<br><br>**Number of study arms:** 2	**Country:** Spain<br>**Number of Participants:** 44<br>**Number Analyzed:** 39<br>**Age (years):** Mean 57.93 (SD 9.23)<br>**Sex:** 100% female<br>**Condition:** Fibromyalgia<br>**Source of Recruitment:** Fibromyalgia Association of Almeria	Waitlist Control: The mindfulness training course was offered to the control group participants after completing all assessment phases.	**Pain Catastrophizing:** 13-item PCS at 7 weeks<br><br>**Intervention Adherence:** See Table 1	Not reported	The authors declare no competing interests.
Andrés-Rodríguez et al., 2019	**Study design:** Individually RCT	**Country:** Spain<br>**Number of Participants:** 70<br>**Number Analyzed:** 57	Usual Care: Participants in both groups received usual care. The treatment provided is mainly pharmacological and adjusted to	**Pain Catastrophizing:** 13-item PCS at 8 weeks	The study was funded in part by the Instituto de Salud Carlos III	The authors report no conflict of interest.

	**Number of study arms:** 2	**Age (years):** Mean 53.36 (SD 8.05)<br>**Sex:** 100% female<br>**Condition:** Fibromyalgia<br>**Source of Recruitment:** Rheumatology service at Parc Sanitari de Sant Joan de Déu	the participants' symptomatic profiles. Counselling about aerobic exercise adjusted to participants' physical limitations is usually provided. Medication intake was monitored during the study and changes in dosages or medications were registered in both the intervention and comparator groups. Changes in medications with known effects on inflammatory status (e.g., corticoids and NSAIDs) were not allowed and were a reason for exclusion from the study.	**Depression:** HADS – depression subscale at 8 weeks<br><br>**Anxiety:** HADS – anxiety subscale at 8 weeks<br><br>**Intervention Adherence:** See Table 1	(ISCIII) of the Ministry of Economy and Competitiveness through the Network for Prevention and Health Promotion in Primary Care (RD16/0007/0005 and RD16/0007/0012), and by a grant for research projects on health from ISCIII (PI15/00383) co-financed by the European Regional Developmental Fund. LAR has a predoctoral contract awarded by the Agency for Management of University and Research Grants (AGAUR; 2018; FI_B00783). AFS has a Sara Borrell research contract from the ISCIII (CD16/00147). APA has a predoctoral contract awarded by the Agency for Management of University and Research Grants (AGAUR; 2017;	

					FI_B00754). JVL has a Miguel Servet research contract from the ISCIII (CP14/00087).	
Astin et al., 2003	**Study design:** Individually RCT<br><br>**Number of study arms:** 2	**Country:** United States of America<br>**Number of Participants:** 128<br>**Number Analyzed:** 64<br>**Age (years):** Mean 47.70 (SD 10.60)<br>**Sex:** 99.2% female<br>**Condition:** Fibromyalgia<br>**Source of Recruitment:** Radio and newspaper advertising, networking with local physicians	Health Education: Eight weekly 2.5-hour sessions. The facilitator presented short lectures on a different topic each week. Topics were drawn principally from the book "Your personal guide to living well with fibromyalgia: A handbook for self-care and treatment". Topics included stress, exercise, pain cycle, emotions, sleep, work, intimacy, and review of current research. Following these presentations, there was time for questions as well as unstructured and open-ended sharing and discussion among group participants.	**Depression:** BDI at 8 weeks<br><br>**Pain:** SF-36 pain subscale at 8 weeks<br><br>**Intervention Adherence:** See Table 1	Grant from the National Center for Complementary and Alternative Medicine, National Institutes of Health (5P50AT00084-03).	Not reported
Bagheri et al., 2021	**Study design:** Individually RCT<br><br>**Number of study arms:** 2	**Country:** Iran<br>**Number of Participants:** 30<br>**Number Analyzed:** 29<br>**Age (years):** Mean 28.30 (SD 7.08)<br>**Sex:** 100% female<br>**Condition:** Patellofemoral Pain<br>**Source of Recruitment:** Flyers and pamphlets posted in physiotherapy clinics and public places, emails to faculty of the University College of Omran and Tosseeh and University of Nahavand	Exercise Intervention: Participants in both groups received an exercise protocol that consisted of 13 exercises (six stretching exercises, seven strength and balance exercises) to be completed in three 60 to 90-minute sessions per week for 18 weeks. The exercise protocol was preceded with a 10-minute warm-up and ended with a 10-minute cool-down, including jogging and general dynamic exercises. Rest intervals between sets and exercises were 30 and 90 seconds respectively. If participants did all the exercises without (1) aggravating their knee	**Pain Catastrophizing:** 13-item PCS at 9 weeks<br><br>**Kinesiophobia:** TSK at 9 weeks<br><br>**Pain Intensity:** VAS (usual pain intensity) at 9 weeks<br><br>**Disability:** KOS at 9 weeks<br><br>**Intervention Adherence:** See Table 1	Not reported	Not reported

			pain, (2) excessive fatigue, and (3) local muscle pain 48 hours after the training session, the training load was increased. Participants were also given instructions on how to manage their training load and modify their running according to their symptoms. At the end of each week, each person had a 10-minute treadmill session and received feedback from a physiotherapist on running technique.	**Mindfulness-Related Adverse Events:** See Table 2		
Banth & Ardebil, 2015	**Study design:** Individually RCT<br><br>**Number of study arms:** 2	**Country:** Iran<br>**Number of Participants:** 88<br>**Number Analyzed:** Not reported<br>**Age (years):** Mean 40.30 (SD 8.20)<br>**Sex:** 100% female<br>**Condition:** Nonspecific chronic low back pain<br>**Source of Recruitment:** Physiotherapy clinics in Ardebil	Usual Care: Participants in both groups could continue with their usual medical care, including physiotherapy sessions and medicine.	**Pain Intensity:** McGill Pain Questionnaire at 8 weeks	None	None declared
Brown & Jones, 2013	**Study design:** Individually RCT<br><br>**Number of study arms:** 2	**Country:** United Kingdom<br>**Number of Participants:** 40<br>**Number Analyzed:** 28<br>**Age (years):** 46.60 (SD 10.90)<br>**Sex:** 75% female<br>**Condition:** Chronic musculoskeletal pain<br>**Source of recruitment:** Electronic medical records	No intervention	**Pain Control:** Survey of Pain Attitudes, brief version – pain control subscale at 8 weeks	This work was funded by the Mind and Life Institute, Arthritis Research UK, and the University of Manchester. The funders had no role in study design, data collection and analysis, decision to publish, or preparation of the manuscript.	There are no financial or other relationships that might lead to a conflict of interest in publishing this article.

Burns et al., 2022 [b]	**Study design:** Individually RCT<br><br>**Number of study arms:** 4	**Country:** United States of America<br>**Number of Participants:** 521<br>**Number Analyzed:** 521<br>**Age (years):** Mean 52.88 (SD 12.16)<br>**Sex:** 57.9% female<br>**Condition:** Chronic low back pain<br>**Source of Recruitment:** University Pain Center, Midwest Orthopedic Clinic and Neurosurgery Clinic at Rush University, Duke University Medical Center pain clinics (e.g., Pain Clinic, Spine Clinic, Neurosurgery Clinic, and Orthopedic Clinic), community physician waiting rooms, local newspaper advertisements	1 – Behavior Therapy: Taught that when pain persists, learned, maladaptive patterns of daily activity often develop. Behavior therapy aimed to help participants better understand how activity patterns can contribute to pain-related problems and learn ways to increase their involvement in activities that are important and meaningful. Involved keeping a diary of activity patterns to increase awareness, setting and tracking completion of daily activity goals, pleasant activity scheduling, brief relaxation exercises to award accomplishment of activities, training in problem solving to meet behavior change goals, training in communication skills to improve communication with others about progress and problems faced, and developing a written maintenance plan that included a list of short-term and long-term activity goals and a plan for dealing with possible setbacks.<br><br>2 – Cognitive Therapy: Used to help participants recognize the relationships between thoughts, feelings, and behaviors. Techniques helped participants a) identify automatic pain-related thoughts; b) evaluate automatic thoughts for accuracy, identify sources of distorted thoughts, and recognize connection between automatic thoughts and emotional and	**Depression:** CES-D Short Form at 8 weeks<br><br>**Pain Intensity:** NRS at 8 weeks<br><br>**Disability:** SF-36 physical function subscale at 8 weeks<br><br>**Intervention Adherence:** See Table 1	Supported by a research grant from the National Institutes of Health/National Institute of Nursing Research (Grant number R01 NR013910).	The authors have no conflicts of interest to declare.

			physical shifts; c) challenge negative, distorted automatic thoughts using "evidence"; d) develop new realistic alternative cognitive appraisal responses; and e) practice applying new rational appraisals and beliefs. Participants developed a written maintenance plan that included a list of short-term and long-term goals for applying cognitive restructuring and a plan for dealing with possible setbacks in their cognitive change efforts.<br><br>3 – No Intervention: Participants could continue with their ongoing treatment regimens for pain, including psychotropic and analgesic medications.			
Cash et al., 2015	**Study design:** Individually RCT<br><br>**Number of study arms:** 2	**Country:** United States of America<br>**Number of Participants:** 91<br>**Number Analyzed:** 91<br>**Age (years):** Not reported<br>**Sex:** 100% female<br>**Condition:** Fibromyalgia<br>**Source of Recruitment:** Two television news broadcasts and newspaper advertisements	Waitlist Control: Control participants were offered the MBSR program after the conclusion of the study, subsequent to their provision of data at the baseline and follow-up assessment waves.	**Pain Intensity:** VAS (pain level over preceding week) at 8 weeks<br><br>**Disability:** FIQ – physical function subscale at 8 weeks<br><br>**Intervention Adherence:** See Table 1	This was an investigator-initiated study funded by faculty startup monies awarded to SES by the Department of Psychiatry and Behavioral Sciences at the University of Louisville School of Medicine. AF was supported by a grant from the National Cancer Institute. LAZ was supported by a doctoral fellowship from the	The authors declare that they have no conflict of interest.

					University of Louisville School of Interdisciplinary and Graduate Studies. SES was supported by grants from the National Institute on Aging and the National Institute of Mental Health. ED was supported by a Career Development Award from the Clinical Science Research and Development Service of the Veterans Affairs Office of Research and Development. The funders played no role in the design, conduct or analysis of the study, nor in the interpretation and reporting of the study findings.	
Chaharmahali et al., 2023	**Study design:** Individually RCT<br><br>**Number of study arms:** 3	**Country:** Iran<br>**Number of Participants:** 60<br>**Number Analyzed:** 52<br>**Age (years):** Mean 54.37 (SD 5.94)<br>**Sex:** 100% female<br>**Condition:** Knee osteoarthritis<br>**Source of Recruitment:** Specialized rheumatology clinic	1 – Neuromuscular Exercises Alone: Neuromuscular exercises performed under the supervision of a certified trainer four times per week for six weeks. Each session lasted 60 minutes (10-minute warm-up, 40 minutes of neuromuscular exercises, and 10-minute cool-down). Each exercise was performed in 2-3 sets with 10-	**Pain Intensity:** VAS at 6 weeks<br><br>**Disability:** WOMAC at 6 weeks	The Razi University supported this work.	No potential conflict of interest was reported by the authors.

			15 repetitions. The neuromuscular exercises targeted various aspects of sensorimotor performance, such as strength, coordination, balance, and proprioception.<br><br>2 – Neuromuscular Exercises + Motivational Interviewing: The motivational interviewing intervention was a client-centered psychological intervention to solve participants' doubts about changing behavior by strengthening motivation and commitment to change. The intervention consisted of three phases: (1) Enhance motivation to exercise; (2) Strengthen commitment to exercise; and (3) Relapse prevention. Broadly the intervention involved: education about the benefits of exercise and consequences of inactivity, determining short-term and long-term exercise goals, helping the participant to develop a change plan using a change plan worksheet, identifying individual situations and obstacles to pursuing goals, asking for commitment to the goals, and praising and reinforcing progress. Initial sessions were face-to-face with a clinical psychologist and lasted 45 to 60-minutes. Subsequent sessions were remote and lasted 10 to 15-minutes. Participants were encouraged to stay in touch with their mentors by phone, email, or online.			

Chen et al., 2021	**Study design:** Individually RCT<br><br>**Number of study arms:** 2	**Country:** China<br>**Number of Participants:** 94<br>**Number Analyzed:** 94<br>**Age (years):** Mean 64.00 (SD 4.33)<br>**Sex:** 42.6% female<br>**Condition:** Arthritis<br>**Source of Recruitment:** Second Hospital of Hainan Medical University	Intensive Education Program Alone: Both groups received routine care and an eight-week intensive education program. Information about arthritis and diabetes was presented, including the pathogeneses of arthritis and diabetes, their clinical manifestations, drug guidance and complications, so as to enable the participants to have a comprehensive understanding of the diseases and their treatment methods and outcomes.	**Depression:** Symptom Checklist 90 – depression subscale at 8 weeks<br><br>**Anxiety:** Symptom Checklist 90 – anxiety subscale at 8 weeks	Not reported	None
Cherkin et al., 2016 [c,d]	**Study design:** Individually RCT<br><br>**Number of study arms:** 3	**Country:** United States of America<br>**Number of Participants:** 342<br>**Number Analyzed:** 342<br>**Age (years):** Mean 49.30 (SD 12.30)<br>**Sex:** 65.7% female<br>**Condition:** Nonspecific chronic low back pain<br>**Source of Recruitment:** Group Health, a large integrated healthcare system in Washington State	1 – Usual Care Alone: Participants in all groups continued to receive their usual medical care throughout the study. Participants in the usual care alone group received US$50 compensation.<br><br>2 – Cognitive Behavioral Therapy (CBT): Eight weekly 2-hour face-to-face group sessions that provided: (1) education about chronic pain, relationships between thoughts and emotional and physical reactions, sleep hygiene, relapse prevention, and maintenance of gains; and (2) instruction and practice in changing dysfunctional thoughts, setting and working toward behavioral goals, relaxation skills (abdominal breathing, progressive muscle relaxation, and guided imagery), activity pacing, and pain-coping strategies. Participants were encouraged to practice the CBT techniques daily between sessions. There were also	**Pain Catastrophizing:** 13-item PCS at 8 weeks and 12 months<br><br>**Chronic Pain Acceptance:** CPAQ-8 at 8 weeks and 12 months<br><br>**Pain Self-Efficacy:** PSEQ at 8 weeks and 12 months<br><br>**Depression:** PHQ-8 at 8 weeks and 12 months<br><br>**Anxiety:** GAD-2 at 8 weeks† and 12 months<br><br>**Pain Intensity:** VAS (average back pain in previous month) at 8 weeks and 12 months	This research was supported by the National Center for Complementary and Integrative Health of the National Institutes of Health under award number R01AT006226.	All authors completed and submitted the ICMJE Form for Disclosure of Potential Conflicts of Interest and none were reported.

			assigned readings to complete from "The Pain Survival Guide: How to Reclaim Your Life". Participants were provided with workbooks and audio recordings of relaxation and imagery exercises. CBT was delivered by four licensed PhD-level psychologists experienced in group and individual CBT for chronic pain.	**Disability:** RDQ (modified to 23 items rather than the original 24 and to ask about the past week rather than today only) at 8 weeks, 12 months, and 24 months<br><br>**Intervention Adherence:** See Table 1<br><br>**Mindfulness-Related Adverse Events:** See Table 2		
Dalili & Bayazi, 2019	**Study design:** Individually RCT<br><br>**Number of study arms:** 2	**Country:** Iran<br>**Number of Participants:** 28<br>**Number Analyzed:** 28<br>**Age (years):** Not reported<br>**Sex:** Not reported<br>**Condition:** Rheumatoid arthritis<br>**Source of Recruitment:** Jam Rheumatology Center in Mashhad	Usual Care	**Depression:** DASS-21 – depression subscale at 8 weeks<br><br>**Anxiety:** DASS-21 – anxiety subscale at 8 weeks	Not reported	The author declares that he has no competing interests.
Davis et al., 2013	**Study design:** Individually RCT<br><br>**Number of study arms:** 2	**Country:** United States of America<br>**Number of Participants:** 79<br>**Number Analyzed:** 79<br>**Age (years):** Mean 46.16 (range 22 to 81)<br>**Sex:** 98% female<br>**Condition:** Fibromyalgia<br>**Source of Recruitment:** Online postings on Facebook and fibromyalgia support group websites, individuals who expressed an interest in	Health Tips: Served as an attention control to account for exposure to the online delivery technology, positive expectancy of treatment effects, and any effects of reporting on one's daily experiences. Health Tips participants were given access to 12 modules of health behavior information in a format similar to that of the mindfulness intervention. Each module introduced a health behavior	**Pain Self-Efficacy:** 0-5 Likert Scale; Scores from two questions were aggregated: "How satisfied are you with how you coped with your symptoms [today]?" and "If you had this level of pain again, how certain are you that you would be able to cope well with	This research was supported by grants from the Arizona Institute for Mental Health Research to MCD and Pfizer to AJZ.	The authors have no conflict of interest to disclose.

		participation in research during recruitment for unrelated research projects	concern (e.g., diet, exercise, sleep), and participants were advised about what constituted a healthy course of action to follow in daily life. In contrast to the mindfulness group, the Health Tips participants received no explicit instruction or encouragement regarding how to implement the health behaviors in daily life.	its negative aspects?" at 6 weeks<br><br>**Pain Intensity:** VAS (average level of pain today) at 6 weeks<br><br>**Intervention Adherence:** See Table 1		
Day et al., 2019	**Study design:** Individually RCT<br><br>**Number of study arms:** 3	**Country:** Australia<br>**Number of Participants:** 69<br>**Number Analyzed:** 69<br>**Age (years):** Mean 50.74 (SD 14.43)<br>**Sex:** 52% female<br>**Condition:** Chronic low back pain<br>**Source of Recruitment:** Physician referral, variety of media outlets, brochures posted within local medical clinics	Cognitive Therapy: Eight weekly 2-hour group-based in-person sessions. Involved traditional Beckian style column technique restructuring exercises but did not include encouragement to become more active or any teaching or encouragement of relaxation, mindfulness, or other behavioral practices or changes. The cognitive restructuring technique was used to help participants recognize the relationships between thoughts, feelings, behaviors, and pain. At-home skill-building activities included a daily record of automatic thoughts concerning pain, as well as other cognitive therapy exercises (e.g., thought-shifting techniques, coping statements) and weekly reading materials/handouts.	**Depression:** PROMIS – depression domain at 8 weeks<br><br>**Pain Intensity:** NRS at 8 weeks<br><br>**Disability:** PROMIS – physical function domain at 8 weeks<br><br>**Intervention Adherence:** See Table 1<br><br>**Mindfulness-Related Adverse Events:** See Table 2	This research was supported by an Early Career Fellowship Grant from the National Health and Medical Research Council, as well as start-up funds from The University of Queensland, awarded to the first author.	The authors have no conflicts of interest to report.
Day et al., 2024	**Study design:** Individually RCT<br><br>**Number of study arms:** 3	**Country:** United States of America<br>**Number of Participants:** 302<br>**Number Analyzed:** 263<br>**Age (years):** Mean 49.10 (SD 14.00)<br>**Sex:** 78.6% female	1 – Cognitive Therapy: Participants were taught cognitive restructuring techniques, including how to identify automatic thoughts and recognize the connection between thoughts and feelings, behaviors, and pain. Specifically, participants were taught to (1) identify	**Depression:** PROMIS – depression domain at 4 weeks<br><br>**Anxiety:** PROMIS – anxiety domain at 4 weeks	This research was supported by the National Center for Complementary and Integrative Health of the National Institutes	The authors declare that they have no competing interests.

		**Condition:** Chronic low back pain<br>**Source of Recruitment:** University of Washington Medicine patients and research participant pool, pain and rehabilitation clinics, clinician referrals, news releases with the University of Washington Newsroom, ResearchMatch.org, social medial, postings on research and pain-related websites	automatic thoughts related to pain, including negative or unrealistic automatic thoughts; (2) evaluate automatic thoughts for accuracy; (3) identify sources and types of distorted or unhelpful thoughts; (4) recognize the connection between automatic thoughts and emotional and physical shifts; (5) challenge negative, distorted automatic thoughts via “weighing the evidence”; (6) develop new realistic alternative cognitive appraisals; and (7) practice applying new appraisals and beliefs.<br><br>2 – Behavioral Activation: Participants were given information about the negative impacts of inactivity and behavioral avoidance on chronic pain and function. Participants were taught: (1) how to be aware of the activities they avoid because of fear of increasing their pain; (2) how to set effective goals so that, step by step, they can start being more active and resume some activities they enjoyed in the past but are currently avoiding; and (3) behavioral pacing skills, to facilitate an increase in appropriate activity level that is consistent with their goals. They were also encouraged to identify activity goals that were pleasurable and/or meaningful and to develop plans for achieving these activity goals.	**Pain Intensity:** NRS which asked participants to rate their average pain over the past week, on an 11-point scale ranging from 0 (“No pain”) to 10 (“Pain as bad as you can imagine") at 4 weeks<br><br>**Disability:** PROMIS – physical function domain at 4 weeks<br><br>**Mindfulness-Related Adverse Events:** See Table 2	of Health under Award Number 1 R01 AT008559-01A1 and the National Center for Advancing Translational Sciences of the National Institutes of Health under Award Number UL1 TR002319. The content is solely the responsibility of the authors and does not necessarily represent the official views of the National Institutes of Health. The REDCap instance used was supported by the Institute of Translational Health Sciences, which is funded by the National Center for Advancing Translational Sciences of the National Institutes of Health under award number UL1TR002319.	

			In both groups, sessions lasted 1.5-hours and were held twice weekly for four weeks. Sessions were delivered via Zoom (videoconferencing software). Participant workbooks specific to the respective treatment allocation were provided for participants to refer to during the group sessions, as well as additional readings and homework assignments to complete between sessions.			
de Oliveira Melchior et al., 2025	**Study design:** Individually RCT<br><br>**Number of study arms:** 2	**Country:** Brazil<br>**Number of Participants:** 82<br>**Number Analyzed:** 53<br>**Age (years):** Mean 34.40 (SD 6.97)<br>**Sex:** 100% female<br>**Condition:** Chronic painful temporomandibular disorder<br>**Source of Recruitment:** Specialist center at the University of São Paulo, social media	No intervention	**Pain Catastrophizing:** PCS – Brazilian Portuguese version at 8 weeks<br><br>**Depression:** DASS-21 – depression subscale at 8 weeks<br><br>**Anxiety:** DASS-21 – anxiety subscale at 8 weeks<br><br>**Intervention Adherence:** See Table 1	This work was supported by the Fundação de Amparo à Pesquisa do Estado de São Paulo (2022/05658-3) CAPES-PROEX (Finance Code: 001).	The authors declare no conflicts of interest.
Diez et al., 2022	**Study design:** Individually RCT<br><br>**Number of study arms:** 2	**Country:** Spain<br>**Number of Participants:** 96<br>**Number Analyzed:** 67<br>**Age (years):** Mean 53 (SD 10)<br>**Sex:** 66% female<br>**Condition:** Chronic back pain<br>**Source of Recruitment:** Email databases of the Eduardo Anitua Foundation, Complutense de Madrid University, and Nirakara Lab	No intervention	**Pain Catastrophizing:** 13-item PCS at 8 weeks<br><br>**Chronic Pain Acceptance:** CPAQ-R at 8 weeks<br><br>**Depression:** DASS-21 – depression subscale at 8 weeks	The author(s) received no specific funding for this work.	The authors have declared that no competing interests exist.

				**Anxiety:** DASS-21 – anxiety subscale at 8 weeks<br><br>**Disability:** RDQ at 8 weeks<br><br>**Mindfulness-Related Adverse Events:** See Table 2		
Donnino et al., 2021	**Study design:** Individually RCT<br><br>**Number of study arms:** 3	**Country:** United States of America<br>**Number of Participants:** 38<br>**Number Analyzed:** 35<br>**Age (years):** Mean 40.32 (SD 13.20)<br>**Sex:** 60% female<br>**Condition:** Chronic back pain<br>**Source of Recruitment:** Physician referrals, flyers posted in the Bostan metropolitan area, social media	1 – Usual Care: Participants randomized to the usual care arm continued their prescribed treatment regimens under guidance of their physicians and without influence from the study team.<br><br>2 – Psychophysiologic Symptom Relief Therapy: A 12-week (38 hour) course. During the first four weeks, participants participated in a program based on Sarno's psychophysiologic model of chronic back pain which included use of the books "Healing Back Pain", "Unlearn Your Pain", and "Worry Less, Live More: The Mindful Way Through Anxiety Workbook". These sessions were held twice a week, 2-hours per session. The intervention was delivered by a physician and mind-body expert with 20 years of experience in patient support or counselling, supported by consultation or training from experts in the field of chronic pain. The overall program was	**Anxiety:** Pain Anxiety Symptom Scale at 8 weeks<br><br>**Disability:** RDQ at 8 weeks<br><br>**Intervention Adherence:** See Table 1	The study was funded by a philanthropic donation by Adam D'Angelo.	The authors have no conflicts of interest to declare.

			constructed around four components: psychophysiologic pain education, returning to physical activity/desensitization, emotional expression, and stress reduction. The psychophysiologic pain education allowed participants to explore their pain history and learn about the clinical presentation of psychophysiological syndromes with the goal of achieving acceptance of the psychological origins of their pain. After completing the educational component, desensitization techniques were used with the goal of reversing the conditioned response to a physical stimulus that was not the primary cause of the pain. Desensitization techniques included imagining doing a physical activity with visual motor imagery (e.g., bending to pick up heavy books) that would typically elicit pain. After pain could no longer be elicited by visualization, participants were encouraged to gradually resume physical activity similar to levels undertaken before pain onset. After the first four weeks, the course introduced the concept of mindfulness with a cognitive component incorporated into the classic MBSR program, with 2-hour sessions once a week for the remaining eight weeks including one full day session of 6-hours.			

Farzadkia et al., 2023 [e]	**Study design:** Individually RCT<br><br>**Number of study arms:** 3	**Country:** Iran<br>**Number of Participants:** 36<br>**Number Analyzed:** 36<br>**Age (years):** Mean 39.10 (SD 5.83)<br>**Sex:** 100% female<br>**Condition:** Fibromyalgia<br>**Source of Recruitment:** Rheumatology Clinic of Rasool Akram Hospital in Tehran	1 – Waitlist Control: The waitlist control group did not receive any treatment. At the end of the study, they received either the MBSR or Intensive Short-Term Dynamic Psychotherapy program as a training package.<br><br>2 – Intensive Short-Term Dynamic Psychotherapy (ISTDP): Eight weekly 2-hour sessions. Based on Dovanlo's ISTDP intervention, which was implemented according to the psychological structure and the ego strength and problems of each participant.	**Depression:** BDI at 8 weeks	This study was self-funded by the authors.	The authors declared no conflict of interest.
Fogarty et al., 2019 [f]	**Study design:** Individually RCT<br><br>**Number of study arms:** 2	**Country:** New Zealand<br>**Number of Participants:** 51<br>**Number Analyzed:** 51<br>**Age (years):** Mean 53.5 (SD 12.5)<br>**Sex:** 88.2% female<br>**Condition:** Rheumatoid arthritis<br>**Source of Recruitment:** Rheumatology clinics at the Auckland and Counties-Manukau District Health Boards, Arthritis New Zealand	Waitlist Control: Participants in the waitlist control group were informed that they would be invited to participate in the mindfulness-based program after all data collection was completed. All participants received routine rheumatology care throughout the study.	**Depression:** HADS – depression subscale at 8 weeks<br><br>**Anxiety:** HADS – anxiety subscale at 8 weeks<br><br>**Pain Intensity:** VAS at 8 weeks<br><br>**Intervention Adherence:** See Table 1<br><br>**Mindfulness-Related Adverse Events:** See Table 2	This study was funded by a grant from The Oakley Mental Health Foundation.	The authors declare that they have no competing interests.
Liu et al., 2025	**Study design:** Individually RCT<br><br>**Number of study arms:** 2	**Country:** China<br>**Number of Participants:** 244<br>**Number Analyzed:** 244<br>**Age (years):** Mean 52.40 (SD 5.05)<br>**Sex:** 57.4% female	Usual Care Alone: All participants received usual care; either conservative management or surgical management. Conservative management involved activity restrictions, oral	**Pain Intensity:** Neer Shoulder Score – pain subscale at 8 weeks	This work was supported by the National Natural Science Foundation of China (No.	The authors have no conflicts of interest to disclose.

		**Condition:** Frozen shoulder<br>**Source of Recruitment:** Hospital sports medicine department	non-steroidal anti-inflammatory drugs, and physiotherapy exercises emphasizing shoulder flexion, external rotation, and extension functions. Surgical approaches involved arthroscopic procedures, including capsular release, middle glenohumeral ligament release, subscapularis release, and synovial membrane clearance.		81601901), Natural Science Foundation of Liaoning, China (No. 2019-MS-079), Peak Climbing Program, Dalian (No. 2022DF012), and Dalian Science and Technology Innovation Fund (No. 2023JJ13SN051).	
Marais et al., 2022	**Study design:** Individually RCT<br><br>**Number of study arms:** 2	**Country:** France<br>**Number of Participants:** 40<br>**Number Analyzed:** 23<br>**Age (years):** Mean 63.00 (SD 7.40)<br>**Sex:** 22.5% female<br>**Condition:** Knee or hip osteoarthritis<br>**Source of Recruitment:** Rheumatology Department at Montpellier University Hospital	Usual Care Alone: Usual care management was similar in both groups and included advice on the importance of weight loss, and physical activity and self-management education delivered via a booklet given to the participants. Management of pain and depressive symptoms was carried out according to standard practice.	**Depression:** HADS – depression subscale at 3 months<br><br>**Anxiety:** HADS – anxiety subscale at 3 months<br><br>**Pain Intensity:** VAS at 3 months<br><br>**Disability:** WOMAC at 3 months<br><br>**Intervention Adherence:** See Table 1	This research did not receive any specific grant from funding agencies in the public, commercial, or not-for-profit sectors.	The authors declare that they have no competing interests.
Mohammadi et al., 2011	**Study design:** Individually RCT<br><br>**Number of study arms:** 2	**Country:** Iran<br>**Number of Participants:** 30<br>**Number Analyzed:** Not reported<br>**Age (years):** Mean 46.31 (SD 14.50)<br>**Sex:** 100% female<br>**Condition:** Chronic musculoskeletal pain	No intervention	**Pain Intensity:** Graded Classification of Chronic Pain – perceived pain subscale at 8 weeks<br><br>**Disability:** Pain Disability	None	None

		**Source of Recruitment:** Physiotherapy service at the Akhwan Hospital in Tehran		Questionnaire at 8 weeks		
Morone et al., 2008	**Study design:** Individually RCT<br><br>**Number of study arms:** 2	**Country:** United States of America<br>**Number of Participants:** 37<br>**Number Analyzed:** 37<br>**Age (years):** Mean 74.83 (SD 5.57)<br>**Sex:** 56.8%<br>**Condition:** Chronic low back pain<br>**Source of Recruitment:** Adult pain clinic, flyers posted around the University of Pittsburgh Medical Center, newspaper advertisements	Waitlist Control: Waitlist control participants did not receive any intervention during the eight-week period when the intervention group were completing the mindfulness meditation program. Once the intervention was completed and participants and waitlist controls had completed all post-intervention measures, the mindfulness meditation program was offered to the waitlist controls.	**Chronic Pain Acceptance:** CPAQ-R at 8 weeks<br><br>**Pain Intensity:** SF-MPQ at 8 weeks<br><br>**Disability:** RDQ at 8 weeks<br><br>**Intervention Adherence:** See Table 1<br><br>**Mindfulness-Related Adverse Events:** See Table 2	Supported by AG23641 K07 of Dr. Stephanie Studenski from the National Institutes of Health. During the time of this work Dr. Morone was supported by a primary care faculty development training grant (HRSA D55 HP05156) and by the National Institutes of Health Roadmap Multidisciplinary Clinical Research Career Development Award Grant (1KL2RR024154-01).	Not reported
Morone et al., 2009	**Study design:** Individually RCT<br><br>**Number of study arms:** 2	**Country:** United States of America<br>**Number of Participants:** 40<br>**Number Analyzed:** 35<br>**Age (years):** Mean 75.30 (SD 7.00)<br>**Sex:** 62.9% female<br>**Condition:** Chronic low back pain<br>**Source of Recruitment:** Local newspaper advertisements, posted flyers, brochures in the	Health Education: Eight weekly 90 minute in-person group sessions. The health education curriculum involved lectures and group discussion. The overall theme was brain health. Specific topics included: 1) pain medications; 2) complementary treatments for back pain (focusing on chiropractic); 3) types of back pain; 4) role of physical therapy in treating back pain; 5) eating and	**Pain Self-Efficacy:** CPSES 8 weeks<br><br>**Pain Intensity:** McGill Pain Questionnaire at 8 weeks<br><br>**Disability:** RDQ at 8 weeks	During the time of this work Dr. Morone was funded by the National Institutes of Health (NIH) Roadmap Multidisciplinary Clinical Research Career Development	There are no financial or other conflicts of interests to declare.

		University of Pittsburgh Medical Centre	health; and 6) Alzheimer's. For each class, 45 to 60 minutes were devoted to a lecture given by a health professional on the topic for the session and 30 to 45 minutes were spent doing a class "brain" exercise and discussion. Participants were given the use of a Nintendo DS "Brain Age" game and encouraged to do this as daily homework, as well as homework assignments from the book "Keep Your Brain Alive" related to the topics discussed in the weekly sessions.	**Intervention Adherence:** See Table 1<br><br>**Mindfulness-Related Adverse Events:** See Table 2	Award Grant (1KL2RR024154-04). This publication was also made possible by Grant Number UL1RR024153 from the National Center for Research Resources (NCRR), a component of the NIH and NIH Roadmap for Medical Research. Its contents are solely the responsibility of the authors and do not necessarily represent the official view of NCRR or NIH.	
Morone et al., 2016	**Study design:** Individually RCT<br><br>**Number of study arms:** 2	**Country:** United States of America<br>**Number of Participants:** 282<br>**Number Analyzed:** 282<br>**Age (years):** Mean 75.00 (SD 7.20)<br>**Sex:** 67% female<br>**Condition:** Chronic low back pain<br>**Source of Recruitment:** Community dwelling individuals	Health Education: An 8-week health education program based on a successful aging curriculum known as the 10 Keys to Healthy Aging. The 10 Keys to Healthy Aging curriculum includes information on key health topics relevant to healthy aging such as hypertension management. The classes were presented by local experts in the field. The same chair stretches taught in the mindfulness intervention were also taught in the health education program. To ensure that the control group received an equal amount of attention and social support, they	**Pain Catastrophizing:** Catastrophizing Scale of the Coping Strategies Questionnaire at 8 weeks<br><br>**Pain Self-Efficacy:** CPSES – self-efficacy for pain management subscale at 8 weeks<br><br>**Pain Intensity:** NRS (average pain) at 8 weeks	This study was supported by grant R01 AG034078 from the National Institutes of Health.	None

			also received monthly booster classes, which met monthly for one hour.	**Disability:** RDQ at 8 weeks<br><br>**Intervention Adherence:** See Table 1<br><br>**Mindfulness-Related Adverse Events:** See Table 2		
Pal et al., 2023	**Study design:** Individually RCT<br><br>**Number of study arms:** 2	**Country:** India<br>**Number of Participants:** 170<br>**Number Analyzed:** 165<br>**Age (years):** Mean 44.70 (SD 13.30)<br>**Sex:** 72% female<br>**Condition:** Chronic musculoskeletal pain<br>**Source of Recruitment:** Hospital outpatient departments	Pain Education: In these sessions, different pain education, self-care, and relaxation techniques (excluding mindfulness concepts) were discussed, and participants were encouraged to practice daily in a similar schedule to the mindfulness group. There was a provision for pain education participants to join the mindfulness program after the stipulated study period, if they desired.	**Pain Catastrophizing:** 13-item PCS at 5 weeks<br><br>**Chronic Pain Acceptance:** CPAQ-8 at 5 weeks<br><br>**Pain Intensity:** NRS at 5 weeks<br><br>**Mindfulness-Related Adverse Events:** See Table 2	None	There are no conflicts of interest.
Pal et al., 2024	**Study design:** Individually RCT<br><br>**Number of study arms:** 2	**Country:** India<br>**Number of Participants:** 195<br>**Number Analyzed:** 181<br>**Age (years):** Mean 49.00 (SD 5.92)<br>**Sex:** 28% female<br>**Condition:** Chronic low back pain<br>**Source of Recruitment:** Not reported	Pain Education: Topics including self-care, pain education and relaxation exercises were discussed and practiced. Mindfulness concepts were deliberately excluded. These sessions remained non-specific to qualify as an active intervention.	**Pain Catastrophizing:** 13-item PCS at 5 weeks<br><br>**Chronic Pain Acceptance:** CPAQ-8 at 5 weeks<br><br>**Depression:** DASS-21 – depression subscale at 5 weeks<br><br>**Anxiety:** DASS-21 – anxiety subscale at 5 weeks	None	There are no conflicts of interest.

				**Pain Intensity:** NRS at 5 weeks<br><br>**Disability:** ODI at 5 weeks<br><br>**Intervention Adherence:** See Table 1		
Parra-Delgado & Latorre-Postigo, 2023	**Study design:** Individually RCT<br><br>**Number of study arms:** 2	**Country:** Spain<br>**Number of Participants:** 33<br>**Number Analyzed:** 31<br>**Age (years):** Mean 52.67 (SD 10.08)<br>**Sex:** 100% female<br>**Condition:** Fibromyalgia<br>**Source of Recruitment:** Fibromyalgia Association of Almansa	Usual Care Alone: All study participants continued with their usual medication treatment, medical visits, rehabilitation sessions and activities proposed by the Fibromyalgia Association.	**Depression:** BDI at 8 weeks<br><br>**Pain Intensity:** VAS (lumbar pain) at 8 weeks<br><br>**Intervention Adherence:** See Table 1	Not reported	Not reported
Pérez-Aranda et al., 2019 [g]	**Study design:** Individually RCT<br><br>**Number of study arms:** 3	**Country:** Spain<br>**Number of Participants:** 225<br>**Number Analyzed:** 173<br>**Age (years):** Mean 53.30 (SD 7.80)<br>**Sex:** 98.2% female<br>**Condition:** Fibromyalgia<br>**Source of Recruitment:** Rheumatology Service of Sant Joan de Deu Hospital	1 – Usual Care Alone: All study participants continued with their regular pattern of medication if any. Usual Care Alone group participants were offered participation in the MBSR program at the conclusion of the study.<br><br>2 – FibroQoL + Usual Care: An active treatment comparator. FibroQoL and MBSR were practically equivalent in terms of structure (2-hour weekly sessions for 8 weeks delivered in a group format). FibroQoL matches MBSR on nonspecific factors, but does not contain mindfulness methods, and does not address mindfulness processes. The FibroQoL protocol is divided in two parts: four sessions of psychoeducation in	**Pain Catastrophizing:** 13-item PCS at 8 weeks and 12 months<br><br>**Psychological Distress:** HADS at 8 weeks and 12 months<br><br>**Intervention Adherence:** See Table 1<br><br>**Mindfulness-Related Adverse Events:** See Table 2	The study was funded in part by the Instituto de Salud Carlos III (ISCIII) of the Ministry of Economy and Competitiveness through the Network for Prevention and Health Promotion in Primary Care (RD16/0007/ 0005; RD16/0007/0012), by a grant for research projects on health from ISCIII (PI15/00383) cofinanced by the	The authors have no conflicts of interest to declare.

			which patients receive information about the pathophysiology, diagnosis, and management of fibromyalgia symptoms, and another four sessions of training in self-hypnosis to generate a state of deep relaxation with the objective of achieving control over the body and pain (a focus that is inconsistent with mindfulness) and to imagine one's life in the future without pain. Audiotapes were provided to facilitate practice at home. Sessions were conducted by one or two members of the FibroQoL team (two psychologists, three family physicians, and a rheumatologist).		European Regional Developmental Fund. APA has a predoctoral contract awarded by the Agency for Management of University and Research Grants (AGAUR; FI_B00754). AFS has a Sara Borrell research contract from the ISCIII (CD16/00147). LAR has a predoctoral contract awarded by the Agency for Management of University and Research Grants (AGAUR; FI_B00783). JVL has a Miguel Servet research contract from the ISCIII (CP14/00087).	
Plews-Ogan et al., 2005	**Study design:** Individually RCT<br><br>**Number of study arms:** 3	**Country:** United States of America<br>**Number of Participants:** 30<br>**Number Analyzed:** 23<br>**Age (years):** Mean 46.5 (SD not reported)<br>**Sex:** 77% female<br>**Condition:** Chronic musculoskeletal pain<br>**Source of Recruitment:** General internal medicine	1 – Usual Care: Usual care involved being seen by a primary care physician at least every 3 months with medication adjustments made as indicated.<br><br>2 – Massage: One-hour massage sessions were given once per week for eight weeks by three licensed massage therapists. Massage techniques were at the discretion of the therapists and included	**Pain Intensity:** NRS (pain sensation) at 8 weeks<br><br>**Intervention Adherence:** See Table 1	This study was supported in part by Grant 1D12HP00040-03: Academic Administrative Units in Primary Care, Department of Health and Human Services and in part by the	The authors have no conflicts of interest to declare for this article or this research.

		practices at the University of Virginia	Swedish, deep-tissue, neuromuscular, and pressure-point techniques. We specifically excluded music, scented oils, and energy techniques such as Reiki or therapeutic touch.		John W. Kluge Foundation.	
Pradhan et al., 2007	**Study design:** Individually RCT<br><br>**Number of study arms:** 2	**Country:** United States of America<br>**Number of Participants:** 63<br>**Number Analyzed:** 60<br>**Age (years):** Mean 54.00 (SD 10.00)<br>**Sex:** 87% female<br>**Condition:** Rheumatoid Arthritis<br>**Source of Recruitment:** Advertisements in Baltimore newspapers, presentations to rheumatologists, presentations at community health fairs, informational flyers widely distributed through the Maryland Chapter of the Arthritis Foundation	Waitlist Control: Participants in the waitlist control group were offered the MBSR program free of charge at the end of the study.	**Depression:** SCL-90-R – depression subscale at 8 weeks<br><br>**Psychological Distress:** SCL-90-R – general severity index subscale at 8 weeks<br><br>**Intervention Adherence:** See Table 1<br><br>**Mindfulness-Related Adverse Events:** See Table 2	Supported by the National Institutes of Health National Center for Complementary and Alternative Medicine as part of the Specialized Research Center grant (Center for Alternative Medicine Research on Arthritis, grant 1-P50-AT000840) awarded to the Center for Integrative Medicine at the University of Maryland School of Medicine.	Ms Magyari teaches MBSR.
Reiner et al., 2019	**Study design:** Individually RCT<br><br>**Number of study arms:** 2	**Country:** Israel<br>**Number of Participants:** 67<br>**Number Analyzed:** 36<br>**Age (years):** Mean 58.03 (SD 14.90)<br>**Sex:** 72% female<br>**Condition:** Chronic back pain<br>**Source of Recruitment:** Large publicly funded pain clinic in southern Israel	Usual Care Alone: All study participants continued to receive usual care from their primary care or specialist physicians and were asked to inform the research team if they made changes in their usual treatment. Participants in the usual care alone group were told that they would be eligible for the mindfulness program two months following completion of the first assessment.	**Pain Intensity:** BPI – pain severity subscale at 8 weeks<br><br>**Intervention Adherence:** See Table 1	Not reported	The authors declare that they have no conflict of interest.

Robles et al., 2025	**Study design:** Individually RCT<br><br>**Number of study arms:** 2	**Country:** Spain<br>**Number of Participants:** 32<br>**Number Analyzed:** 32<br>**Age (years):** Mean 55.09 (SD 10.16)<br>**Sex:** 64% female<br>**Condition:** Chronic back pain (discopathy)<br>**Source of Recruitment:** Website of a training and research center specializing in meditation and neuroscience	Waitlist Control	**Pain Catastrophizing:** 13-item PCS at 8 weeks<br><br>**Chronic Pain Acceptance:** CPAQ-R at 8 weeks<br><br>**Kinesiophobia:** TSK-11-SV at 8 weeks<br><br>**Depression:** DASS-21 – depression subscale at 8 weeks<br><br>**Anxiety:** DASS-21 – anxiety subscale at 8 weeks<br><br>**Pain Intensity:** VAS (pain at time of assessment) at 8 weeks	This work was supported by a Ministry of Science grant (PID2019-108711GB-I00) awarded to Carmelo Vazquez and a Complutense University predoctoral grant (CT82/20-CT83/20) awarded to Elena Robles.	Not reported
Safavi-Farokhi et al., 2020	**Study design:** Individually RCT<br><br>**Number of study arms:** 2	**Country:** Iran<br>**Number of Participants:** 40<br>**Number Analyzed:** 40<br>**Age (years):** Mean 28.30 (SD 3.66)<br>**Sex:** 100% female<br>**Condition:** Chronic nonspecific low back pain<br>**Source of Recruitment:** Not reported	Stabilizing Exercises Alone: Stability exercises aimed at strengthening the deep trunk muscles, with a particular emphasis on the lumbar multifidus. These exercises were conducted under the supervision of a physiotherapist and were divided into three stages.<br>In the first stage, exercises focused on isometric contraction of the pelvic floor muscles and the multifidus muscle, as well as abdominal contractions. Participants were instructed to pull their stomach in slowly and gently, below the navel, while maintaining a neutral spine position. Exercises	**Kinesiophobia:** TSK at 4 weeks<br><br>**Disability:** ODI at 4 weeks	Financial support was received from the Neuromuscular Rehabilitation Research Center at Semnan University of Medical Sciences.	Not reported

			also included arching with bent knees, involving simultaneous contraction of the multifidus, transversus abdominis, and pelvic floor muscles. The second and third stages involved performing the same exercises while sitting and on all fours. Participants maintained the natural arch of their back and incorporated upper and lower body movements in opposite directions. Due to the difficulty of isolating multifidus muscle contraction, ultrasound biofeedback was used to teach patients how to contract the muscle effectively. During the process, the physiotherapist placed their hand slightly outside the target area and asked the patient to inhale and exhale. The patient was then instructed to hold the exhalation and attempt to contract the multifidus muscle against the physiotherapist's hand. Simultaneously, the patient could observe the muscle contraction on the ultrasound machine. This procedure was repeated several times until the patient learned the correct technique for contracting the muscle.			
Sannigrahi et al., 2022	**Study design:** Individually RCT<br><br>**Number of study arms:** 2	**Country:** India<br>**Number of Participants:** 50<br>**Number Analyzed:** 40<br>**Age (years):** Mean 62.30 (SD 3.90)<br>**Sex:** 100% female<br>**Condition:** Knee osteoarthritis	Balance Exercises Alone: The balance intervention included an introductory presentation and discussion, brief personal interview, personal instruction meeting, and three follow-up small group seminars over the course of one week. The balance exercises	**Pain Intensity:** VAS at 3 months<br><br>**Disability:** WOMAC at 3 months	None	There are no conflicts of interest.

		**Source of Recruitment:** Orthopedic department at Burdwan Medical College	included: one leg balance, blind advanced one-leg balance, cross body swings, tandem walking, and rocker board exercise.	**Intervention Adherence:** See Table 1		
Schmidt et al., 2011 [h]	**Study design:** Individually RCT<br><br>**Number of study arms:** 3	**Country:** Germany<br>**Number of Participants:** 177<br>**Number Analyzed:** 168<br>**Age (years):** Mean 52.51 (SD 9.65)<br>**Sex:** 100% female<br>**Condition:** Fibromyalgia<br>**Source of Recruitment:** Patient self-help groups, news media, referrals from general practitioners, rheumatologists, and the University of Freiburg Medical Center Interdisciplinary Pain Unit	1 – Waitlist Control: Patients randomized to this group received no active treatment but were offered their choice of either intervention at the conclusion of the follow-up period.<br><br>2 – Active Control (Relaxation): This intervention involved an 8-week group program with a similar format and group size to the MBSR program, led by a single instructor. It included equivalent amounts of social support and weekly topical educational discussions. Jacobson Progressive Muscle Relaxation training and fibromyalgia-specific gentle stretching exercises were used as alternatives to the mindfulness and yoga components of the MBSR curriculum. Homework assignments matched the duration and intensity of those in the MBSR group. Participants received compact discs with instructions for daily exercises.	**Depression:** CES-D at 8 weeks<br><br>**Anxiety:** STAI – trait anxiety subscale at 8 weeks<br><br>**Intervention Adherence:** See Table 1	This study was supported by the Samueli Institute and by the Manfred Köhnlechner Stiftung.	No financial or other conflicts of interest exist for any of the authors.
Sephton et al., 2007 [i]	**Study design:** Individually RCT<br><br>**Number of study arms:** 2	**Country:** United States of America<br>**Number of Participants:** 91<br>**Number Analyzed:** 91<br>**Age (years):** Mean 48.00 (SD 10.10)<br>**Sex:** 100% female<br>**Condition:** Fibromyalgia	Waitlist Control: Waitlist control participants were offered the MBSR program only after the conclusion of the study.	**Depression:** BDI at 8 weeks<br><br>**Intervention Adherence:** See Table 1	Supported by intramural research grants from the University of Louisville School of Medicine and Office of the Vice President for Research.	Not reported

		**Source of Recruitment:** Brief television broadcasts, newspaper advertisements				
Shah et al., 2024	**Study design:** Individually RCT<br><br>**Number of study arms:** 2	**Country:** United States of America<br>**Number of Participants:** 40<br>**Number Analyzed:** 36<br>**Age (years):** Mean 65.51 (SD 8.30)<br>**Sex:** 88% female<br>**Condition:** Knee osteoarthritis<br>**Source of Recruitment:** Social media, Craigslist website, local senior and community centers, an existing database of participants from prior studies	Exercise and Education Alone: The exercise program was delivered by a physical therapist. Each 1-hour session included a warm-up, 5-6 lower extremity strengthening exercises, 1-2 neuromuscular and balance exercises, and a cool-down. Before the start of each set, the physical therapist demonstrated the exercise. Each exercise was performed bilaterally in three sets of 8-12 repetitions at an intensity level corresponding to a Rating of Perceived Exertion (RPE) of 4-6 on the modified Borg CR10 scale. Participants were suggested to progress to a more challenging exercise if their RPE was <4 while performing the exercise with proper technique. For home exercises, participants were instructed to perform strengthening exercises at least two other days per week and aerobic exercise of their choice at least five days per week. Participants in both groups were given exercise equipment (resistance bands, step-up platform with adjustable height) and weekly osteoarthritis education materials via email. The participants were also provided with a program manual with information about the exercise intervention and links to online videos of exercises. The	**Pain Catastrophizing:** 3-item PCS at 8 weeks<br><br>**Pain Self-Efficacy:** CPSES – self-efficacy for pain management subscale at 8 weeks<br><br>**Depression:** PHQ-8 at 8 weeks<br><br>**Pain Intensity:** KOOS – pain subscale at 8 weeks<br><br>**Intervention Adherence:** See Table 1<br><br>**Mindfulness-Related Adverse Events:** See Table 2	Investigators were supported by a Boston University Sargent College Student Research Grant, Rheumatology Research Foundation Medical and Graduate Student Preceptorship Award, and National Institutes of Health grant K01AR069720.	None

			educational materials were developed by the study team and included topics such as knee osteoarthritis overview, pain education, comorbidities in knee osteoarthritis, physical activity and exercise, weight management, reducing joint loading, complementary and alternative therapies, and pharmacological and surgical therapies.			
Sharpe et al., 2025	**Study design:** Individually RCT<br><br>**Number of study arms:** 3	**Country:** Australia<br>**Number of Participants:** 306<br>**Number Analyzed:** 247<br>**Age (years):** Mean 58.88 (SD 10.82)<br>**Sex:** 94% female<br>**Condition:** Rheumatoid arthritis<br>**Source of Recruitment:** Social media, the eCentreClinic, through patient organizations (e.g., Arthritis Australia)	1 – Waitlist Control: Those who were allocated to the waiting list were asked to indicate their preference for either cognitive behavioral therapy or MBSR and were given access to their preferred program after the 6-month follow-up.<br><br>2 – Online Cognitive Behavioral Therapy (CBT) Program (The Pain Course): Five lessons delivered over eight weeks. Each lesson took 20-30 minutes. The Pain Course involves lessons based on the principles of CBT, including (1) pain education, (2) cognitive therapy specifically thought monitoring and challenging, (3) pleasant activity scheduling and controlled breathing, (4) activity pacing and graded exposure, and (5) relapse prevention. The Pain Course provides additional optional information that targets common concerns, such as sleep hygiene, problem solving, and managing worry. For the purposes of this study, we adapted the Pain Course	**Depression:** PHQ-9 at 8 weeks<br><br>**Anxiety:** GAD-7 at 8 weeks<br><br>**Pain Intensity:** BPI Short form – pain severity subscale at 8 weeks<br><br>**Disability:** HAQ-D1 at 8 weeks<br><br>**Intervention Adherence:** See Table 1<br><br>**Mindfulness-Related Adverse Events:** See Table 2	This study was funded by a bequest from the Dorothy Tinsley Reavley Bequest for rheumatology research.	The researchers involved in this study developed the two interventions: cognitive behavioral therapy (BFD, adapted to rheumatoid arthritis for current study) and mindfulness (ALS and LS, adapted to rheumatoid arthritis for the current study) but received no financial benefit from these programs.

			by co-developing stories of people with rheumatoid arthritis with people with lived experience.			
Shokouhi Nejad et al., 2022 [j]	**Study design:** Individually RCT<br><br>**Number of study arms:** 2	**Country:** Iran<br>**Number of Participants:** 30<br>**Number Analyzed:** 30<br>**Age (years):** Not reported<br>**Sex:** 100% female<br>**Condition:** Fibromyalgia<br>**Source of Recruitment:** Mehrgan Pain Clinic, Seyed Khandan Pain Clinic, and Talegani Hospital Pain Clinic	1 – No intervention.<br><br>2 – Compassion Therapy: 12 weekly sessions of 75 minutes each. Sessions focused on topics such as the logic of treatment based on compassion, understanding compassion and distinguishing self-compassion from self-pity, forms of self-care, cultivating feelings of warmth and kindness towards oneself and others, teaching forgiveness and acceptance without judgement, tolerance training, and teaching methods to deal with factors that disrupt self-compassion.	**Psychological Distress:** DASS-21 at 8 weeks<br><br>**Pain Intensity:** Chronic Pain Grade Questionnaire at 8 weeks	Not reported	The authors declared no conflict of interest.
Sobhani et al., 2019	**Study design:** Individually RCT<br><br>**Number of study arms:** 2	**Country:** Iran<br>**Number of Participants:** 40<br>**Number Analyzed:** 40<br>**Age (years):** Not reported<br>**Sex:** 100% female<br>**Condition:** Chronic postpartum low back pain<br>**Source of Recruitment:** Saadat Abad Health and Treatment Center in Tehran	Waitlist Control: Participants in the waitlist control were offered the intervention after the end of the study period.	**Depression:** DASS-21 – depression subscale at 8 weeks<br><br>**Anxiety:** DASS-21 – anxiety subscale at 8 weeks<br><br>**Disability:** ODI at 8 weeks	None	Not reported
Taub et al., 2024	**Study design:** Individually RCT<br><br>**Number of study arms:** 2	**Country:** Israel<br>**Number of Participants:** 95<br>**Number Analyzed:** 90<br>**Age (years):** Mean 49.40 (SD 13.20)<br>**Sex:** 77% female<br>**Condition:** Fibromyalgia<br>**Source of Recruitment:** The Center for Autoimmune Diseases at the Sheba Medical	Waitlist Control: Participants assigned to the waitlist control group received no active treatment during the waiting period.	**Pain Catastrophizing:** 13-item PCS at 10 weeks<br><br>**Depression:** PHQ-9 at 10 weeks<br><br>**Disability:** Revised FIQ - physical function subscale at 10 weeks	Not reported	None

		Center in Israel, advertisements posted on social media and sent to organizations supporting fibromyalgia				
Van Gordon et al., 2017	**Study design:** Individually RCT<br><br>**Number of study arms:** 2	**Country:** United Kingdom<br>**Number of Participants:** 148<br>**Number Analyzed:** 106<br>**Age (years):** Mean 46.88 (SD 9.43)<br>**Sex:** 83% female<br>**Condition:** Fibromyalgia<br>**Source of Recruitment:** Fibromyalgia self-help and support groups, general practice clinics	Cognitive Behavioral Therapy for Groups: Involved educating participants in cognitive behavioral theory and principles. It was identical to the mindfulness intervention condition on all non-specific factors such as overall course length, individual session duration, group and one-to-one discussion component, group size, and inclusion of an at-home practice element. Weekly sessions comprised: (1) a taught presentation component (45 minutes), (2) a facilitated group discussion component (30 minutes), (3) guided discovery educational exercises (30 minutes), and (4) the same number and duration of breaks as the mindfulness intervention. The weekly sessions were explicitly education-focused and did not include any practice or discussion of meditation.	**Psychological Distress:** DASS-21 at 10 weeks<br><br>**Pain Intensity:** SF-MPQ at 10 weeks<br><br>**Intervention Adherence:** See Table 1	No financial support was received for this study.	All authors declare no conflict of interest.
Zangi et al., 2012	**Study design:** Individually RCT<br><br>**Number of study arms:** 2	**Country:** Norway<br>**Number of Participants:** 73<br>**Number Analyzed:** 68<br>**Age (years):** Mean 53.90 (SD 9.10)<br>**Sex:** 79% female<br>**Condition:** Inflammatory rheumatic joint diseases<br>**Source of Recruitment:** Rheumatology departments,	Usual Care Alone: All study participants received routine care throughout the study including the provision of a CD with mindfulness-based home exercises for individual voluntary use. Participants in the usual care alone group were informed that they would be invited to participate in the mindfulness-based program	**Pain Self-Efficacy:** Arthritis Self-Efficacy Scale – pain subscale at 15 weeks and 12 months<br><br>**Psychological Distress:** General Health Questionnaire,	The study was supported by Diakonhjemmet Hospital.	None

		two registers at Diakonhjemmet Hospital in Oslo	after all data collection was completed.	20-item version at 15 weeks and 12 months<br><br>**Pain Intensity:** NRS at 15 weeks and 12 months<br><br>**Intervention Adherence:** See Table 1<br><br>**Mindfulness-Related Adverse Events:** See Table 2		
Zautra et al., 2008 [k]	**Study design:** Individually RCT<br><br>**Number of study arms:** 3	**Country:** United States of America<br>**Number of Participants:** 144<br>**Number Analyzed:** 137<br>**Age (years):** Mean 54.20 (SD 13.80)<br>**Sex:** 67% female<br>**Condition:** Rheumatoid arthritis<br>**Source of Recruitment:** Health fairs, Arthritis Foundation, local physicians' offices, rheumatologist referrals at the Carl T. Hayden Veterans Affairs Medical Center in Phoenix	1 – Cognitive Behavioral Therapy (CBT) for Pain: Eight weekly 2-hour sessions. The initial session presented a rationale for and overview of CBT. Sessions within each module addressed specific objectives that reflected the skills participants were helped to achieve. Within each session, therapists introduced didactic information, implemented skill related exercises, reviewed with individual participants their understanding and learning of the specific skills, and assigned weekly homework related to session activities. Therapists reviewed homework at the beginning of each treatment session to reinforce adherence and to problem solve application difficulties. The following modules focused on increasing pain management skills, following a standard CBT format: (a) introduction and review of pain	**Pain Catastrophizing:** Participants were asked to rate their level of agreement with the following two statements: "I worried about whether my pain would ever end" and "I felt my pain was so bad I couldn't stand it anymore," taken from the Coping Strategies Questionnaire. Ratings were made on a scale of 1 (strongly disagree) to 5 (strongly agree), at 8 weeks.<br><br>**Pain Self-Efficacy:** Participants were asked to rate how satisfied they were with how they coped with their pain	Not reported	Not reported

			concepts; (b) relaxation training; (c) autogenic training and other methods of relaxation; (d) activity pacing and managing daily activities; (e) cognitive coping; (f) alternative pain management approaches, memory and concentration; (g) managing intense pain episodes, problem-solving; and (h) relapse prevention, generalization, and maintenance.<br><br>2 – Education: Eight weekly 2-hour sessions. Participants in the education group were recruited to join a group that provided information on ways to manage their arthritis.	at its worst that day on a scale of 1 (very dissatisfied) to 5 (very satisfied). The second item asked for their rating of their degree of certainty that they could “adjust well” to the same level of pain again in the future, at 8 weeks.<br><br>**Pain Control:** Participants rated the degree of their perceived control over pain on a scale from 1 (no control at all) to 10 (complete control) at 8 weeks<br><br>**Depression:** Averaging scores for six items: lack of interest in the day’s activities, increase or decrease in appetite, feeling “restless” or “slowed down”, fatigue or loss of energy, feeling down on oneself, and difficulty concentrating or making decisions. Participants responded with an answer of “yes” or “no” to each Item, at 8 weeks		

				**Pain Intensity:** NRS at 8 weeks<br><br>**Intervention Adherence:** See Table 1		
Zgierska et al., 2016 [l]	**Study design:** Individually RCT<br><br>**Number of study arms:** 2	**Country:** United States of America<br>**Number of Participants:** 35<br>**Number Analyzed:** 35<br>**Age (years):** Mean 51.80 (SD 9.70)<br>**Sex:** 80% female<br>**Condition:** Chronic low back pain<br>**Source of Recruitment:** Electronic health record, brochures mailed to clinicians	Waitlist Control: Those randomly assigned to the waitlist control group were eligible to receive the mindfulness intervention after they completed the study.<br><br>Usual care for chronic low back pain and opioid therapy management was provided to all participants by their regular clinicians. Usual care includes pharmacotherapy safety and treatment progress monitoring, treatment agreements, and referral to specialty care, including physical therapy, and complementary therapies for pain and/or mental health.	**Chronic Pain Acceptance:** CPAQ-R at 8 weeks<br><br>**Pain Intensity:** BPI Short Form – pain severity subscale at 8 weeks<br><br>**Disability:** ODI at 8 weeks<br><br>**Intervention Adherence:** See Table 1<br><br>**Mindfulness-Related Adverse Events:** See Table 2	Dr. Zgierska's work was supported by the grant K23AA017508 from the National Institutes of Health National Institute on Alcohol Abuse and Alcoholism, and funds from the University of Wisconsin-Madison. The project was also supported by the Clinical and Translational Science Award program through the National Institutes of Health National Center for Advancing Translational Sciences, grant UL1TR000427.	The authors have no conflicts of interest to disclose.
Zgierska et al., 2025	**Study design:** Individually RCT<br><br>**Number of study arms:** 2	**Country:** United States of America<br>**Number of Participants:** 770<br>**Number Analyzed:** 770<br>**Age (years):** 57.80 (SD 11.30)	Cognitive Behavioral Therapy (CBT) + Usual Care: Eight weekly 2-hour group sessions delivered by trained, licensed therapists experienced in CBT and trained in	**Pain Intensity:** BPI – pain severity subscale at 3 months and 12 months	This study was funded through award OPD-1601-33860 from the Patient-Centered	Dr Zgierska reported serving on the board of directors and executive committee for the American

		**Sex:** 56.4% female<br>**Condition:** Chronic low back pain<br>**Source of Recruitment:** Community and outpatient settings	the intervention protocol. Sessions were initially delivered in-person, then remotely due to COVID-19 restrictions. Involved a variety of cognitive-behavioral strategies (e.g., cognitive restructuring of maladaptive pain-related beliefs, coping skills, relaxation training, and behavioral activation, including pacing) meant to facilitate active self-management. These strategies lay a foundation for learning to modify, diminish, or reframe negative thoughts and beliefs about pain. The CBT intervention did not include training in mindfulness-based concepts, such as acceptance or nonjudgmental approaches to the pain experience. Participants were encouraged to perform home practice of at least 30 minutes per day on 6 days per week.	**Disability:** ODI at 3 months and 12 months<br><br>**Mindfulness-Related Adverse Events:** See Table 2	Outcomes Research Institute (PCORI). This project also received funding and institutional support through the University of Wisconsin-Madison School of Medicine and Public Health, and its Department of Family Medicine and Community Health; Brigham and Women's Hospital, Harvard Medical School; the University of Utah College of Social Work; and the Penn State College of Medicine Department of Family and Community Medicine. The funders had no role in the design and conduct of the study; collection, management, analysis, and interpretation of the data; preparation, review, or approval of the manuscript;	Society of Addiction Medicine and receiving reimbursement of some of the travel cost 1 to 2 times per year to in-person meetings of this society outside the submitted work. Dr Garland reported serving as the Director of the University of California San Diego ONEMIND (Optimized Neuroscience-Enhanced Mindfulness Intervention Design), which provides mindfulness-oriented recovery enhancement (MORE), mindfulness-based therapy, and cognitive behavioral therapy in the context of research trials for no cost to research participants; receiving honoraria and payment for delivering seminars, lectures, and teaching engagements

					and decision to submit the manuscript for publication.	(related to training clinicians in mindfulness), including those sponsored by institutions of higher education, government agencies, academic teaching hospitals, and medical centers; receiving royalties from the sale of books related to MORE; and being a licensor to BehaVR LLC. No other disclosures were reported.
Zou et al., 2024	**Study design:** Individually RCT<br><br>**Number of study arms:** 2	**Country:** China<br>**Number of Participants:** 118<br>**Number Analyzed:** Not reported<br>**Age (years):** Mean 35.6 (SD 4.10)<br>**Sex:** 39% female<br>**Condition:** Ankylosing spondylitis<br>**Source of Recruitment:** Department of Rheumatology and Immunology of Huizhou Central People's Hospital	Usual Care Alone: Usual care involved: (1) Health education – Disease-related health education was provided to participants, including information about treatment-related matters and self-care; (2) Diet and medication care – Participants were advised to control their salt and fat intake, avoid eating spicy and irritating foods, quit smoking and drinking, given medication guidance, informed of the importance of following their doctor's instructions related to medication, and told to monitor their reactions to medication; (3) Postural Care – Participants were advised to keep their back straight in daily life and to avoid spinal curvature. When resting, participants were advised	**Disability:** Bath Ankylosing Spondylitis – Functional Index at 4 weeks	Not reported	Not reported

			to use low pillows, sleep on hard beds, and lie in supine; (4) Psychological Care – Psychological counselling was provided to participants with negative emotions to guide them to face their disease and help them to regulate their emotions.			
Abbreviations: BDI = Beck Depression Inventory; BPI = Brief Pain Inventory; CES-D = Center for Epidemiological Studies Depression Inventory; CPAQ-8 = Chronic Pain Acceptance Questionnaire 8; CPAQ-R = Chronic Pain Acceptance Questionnaire – Revised; CPSES = Chronic Pain Self-Efficacy Scale; DASS-21 = Depression Anxiety and Stress Scale 21; FIQ = Fibromyalgia Impact Questionnaire; GAD-2 = Generalized Anxiety Disorder-2; GAD-7 = Generalized Anxiety Disorder-7; HADS = Hospital Anxiety and Depression Scale; HAQ-DI = Health Assessment Questionnaire – Disability Index; KOOS = Knee Injury and Osteoarthritis Outcome Score; KOS = Knee Outcome Scale; MBCT = Mindfulness-Based Cognitive Therapy; MBSR = Mindfulness-Based Stress Reduction; NRS = Numerical Rating Scale; NSAIDs = Non-steroidal anti-inflammatory drugs; ODI = Oswestry Disability Index; PCS = Pain Catastrophizing Scale; PHQ-8 = Patient Health Questionnaire-8; PHQ-9 = Patient Health Questionnaire-9; PROMIS = Patient-Reported Outcomes Measurement Information System; PSEQ = Pain Self-Efficacy Questionnaire; PSWQ = Penn State Worry Questionnaire; RCT = Randomized controlled trial; RDQ = Roland-Morris Disability Questionnaire; SCL-90-R = Symptom Checklist 90 Revised; SF-36 = 36-Item Short Form Survey; SF-MPQ = Short-Form McGill Pain Questionnaire; STAI = State Trait Anxiety Inventory; tDCS = Transcranial direct-current stimulation; TSK = Tampa Scale of Kinesiophobia; TSK-11-SV = 11-item shortened version of the Tampa Scale of Kinesiophobia; VAS = Visual Analogue Scale; WOMAC = Western Ontario and McMaster Universities Osteoarthritis Index.						

Additional Reports:

[a] Akbarzadeh M, Sabhani P, Rafienia P, et al. Effectiveness of mindfulness-based cognitive therapy separately and combined with TDCS on pain severity and quality of life in fibromyalgia patients. Rooyesh-e-Ravanshenasi Journal. 2022;11(9):33-44.

[b] Burns J, Jensen M, Thorn B, et al. Cognitive Therapy, Mindfulness-Based Stress Reduction, and Behavior Therapy for the Treatment of Chronic Pain: Predictors and Moderators of Treatment Response. J Pain. 2024;25(6):104460.

[c] Cherkin D, Anderson L, Sherman J, et al. Two-Year Follow-up of a Randomized Clinical Trial of Mindfulness-Based Stress Reduction vs Cognitive Behavioral Therapy or Usual Care for Chronic Low Back Pain. JAMA. 2017;317(6):642–644.

[d] Turner JA, Anderson ML, Balderson BH, et al. Mindfulness-based stress reduction and cognitive behavioral therapy for chronic low back pain: similar effects on mindfulness, catastrophizing, self-efficacy, and acceptance in a randomized controlled trial. Pain. 2016;157(11):2434–2444.

[e] Farzadkia M, Farhangi A, Abolghasemi S. Comparing the Effectiveness of Mindfulness-Based Stress Reduction and Intensive Short-term Dynamic Psychotherapy in Reducing Intolerance of Uncertainty and Depression in Women with Fibromyalgia. Women's health bull. 2023;10(1):44-51.

[f] Fogarty FA, Booth RJ, Gamble GD, et al. The effect of mindfulness-based stress reduction on disease activity in people with rheumatoid arthritis: a randomised controlled trial. Ann Rheum Dis. 2015;74(2):472.

[g] Medina S, O'Daly O, Howard A, et al. Does practice make perfect? Functional connectivity of the salience network and somatosensory network predicts response to mind-body treatments for fibromyalgia. Front Pain Res. 2024;5:e1245235.

[h] Grossman P, Deuring G, Walach H, et al. Mindfulness-Based Intervention Does Not Influence Cardiac Autonomic Control or the Pattern of Physical Activity in Fibromyalgia During Daily Life: An Ambulatory, Multimeasure Randomized Controlled Trial. Clin J Pain. 2017;33(5):385-394.

[i] Weissbecker I, Salmon P, Studts J, et al. Mindfulness-Based Stress Reduction and Sense of Coherence Among Women with Fibromyalgia. J Clin Psychol Med Settings. 2002;9:297-307.

[j] Shokouhi Nejad N, Bayat M, Zanganeh Motlagh F. Comparing the Effectiveness of Cognitive Therapy Based on Mindfulness and Compassion Therapy on Automatic Negative Thoughts, Psychological Symptoms, Pain Intensity, and Quality of Life in Patients With Fibromyalgia. J Arak Univ Med Sci. 2022;25(1):156-173.

[k] Davis M, Zautra A, Wolf L, et al. Mindfulness and cognitive-behavioral interventions for chronic pain: differential effects on daily pain reactivity and stress reactivity. J Consult Clin Psychol. 2015;83(1):24-35.

[l] Zgierska A, Burzinski C, Cox J, et al. Mindfulness Meditation and Cognitive Behavioural Therapy Intervention Reduced Pain Severity and Sensitivity in Opioid-Treated Chronic Low Back Pain: Pilot Findings from a Randomized controlled Trial. Pain Med. 2016;17(10):1865-1881.
