# Supplementary material for: The Effects of Mindfulness‐Based Programs on Pain‐Related Cognitions in Individuals With Chronic Musculoskeletal Pain: A Systematic Review and Meta‐Analysis
Source: Pain Res Manag. 2026 Nov 9;2026:6495015. doi: 10.1155/prm/6495015 (PMC13746321; doi:10.1155/prm/6495015)
Supplement: Supplementary file 1 [file PRM-2026-6495015-s001.zip › Supplemental Digital Content 4_Intervention Characteristics_v4_01072026_CLEAN.pdf]

Supplemental Digital Content 4: Summary of the Characteristics of Interventions in the Included Studies

**Study**	**Brief Name**	**What?** *Mindfulness-Based Program*	**What?** *Prescribed Home Practice*	**What?** *Non-Mindfulness Components*	**Who Provided?**	**How and Where?**	**When and How Much?**	**Tailoring and Modifications**
Akbarzadeh et al., 2022	MBCT	Description of the eight sessions: (1) Included mindful eating, where participants ate a raisin in a mindful way, practicing body inspection, and practicing doing daily activities in a mindful way; (2) Involved body scan practice, ten minutes of mindful breathing, paying attention to one of the daily activities in a different and mindful way, and recording pleasant and desirable events; (3) Involved sitting meditation with a focus on mindful breathing and stretching, walking with mindfulness, practicing a three-minute breathing space, and recording unpleasant and adverse events; (4) Involved five minutes of visual and auditory meditation, sitting meditation, an explanation of the laws governing emotions, and a three-minute breathing space; (5) Covered sitting meditation, focusing on how to react to thoughts, feelings, and bodily sensations, along with a three-minute breathing space; (6) Involved sitting meditation, awareness of breathing and body, engaging with thoughts	A home practice program was prescribed. Further details were not reported.	Nil	Not reported	In-person group sessions. Setting not reported.	**Duration of intervention:** 8 weeks **Number of sessions:** 8 **Session frequency:** 1 session per week **Session length:** 1.5 hours	None reported

		and feelings, and accepting unpleasant thoughts and emotions; (7) Included sitting meditation with awareness of breathing and body position, awareness of the connection between activity and mood, and a three-minute breathing space; (8) Focused on reflecting on the body scan practice, reviewing the entire course, and providing a home exercise program that could be continued for up to a month.						
Amutio et al., 2015	Mindfulness Intervention Program	The mindfulness intervention was partially based on some of the elements and exercises of Kabat-Zinn, mindfulness-related strategies used in acceptance and commitment therapy, along with explanations and discussions of the metaphors and exercises used in this therapy, and stories and tales taken from Zen philosophy and from Vipassana meditation. The main goal of the program is for participants to learn to let their thoughts flow without trying to change them. The aim was the re-education of conditioned and automatic ways of reacting when faced with normally occurring internal and external events. Therefore, instead of trying to fight thoughts, emotions, and sensations, participants are encouraged to let them be and observe how	Participants were requested to practice the body-scan exercise daily for 10 minutes and the mindfulness breathing exercises for 30 minutes.	Nil	An experienced mindfulness teacher.	In-person group sessions. Setting not reported.	**Duration of intervention:** 7 weeks **Number of sessions:** 7 **Session frequency:** 1 session per week **Session length:** 2 hours	None reported

		they come and go. In this way, they learn how to break the habit of letting their thoughts, emotions, and sensations control them. Participants become aware of their presence, but they do not dwell on them, either on their content or veracity, considering them as events that are constantly flowing. Sessions used the following structure: (1) Participants' reflections about their mindfulness meditation exercise practice during the week; (2) Practice of body scan for 10 minutes; (3) Presentation of several metaphors through different animations and stories, and also some exercises for each of the sessions (e.g., Unwelcome Party Guest metaphor, It's a Beautiful Day metaphor, observing physical sensations of different body parts, breathing exercises, observing thoughts, accepting uncomfortable private events); (4) Practice of mindfulness, attending to the breath for 30 minutes.						
Amutio et al., 2022	Meditación-Fluir	The intervention is based on the following traditions and techniques: (1) Exercises from Kabat Zinn’s MBSR program; (2) Mindfulness techniques used in acceptance and commitment therapy (e.g.,	Participants were requested to undertake mindful body scan and full awareness of breathing	Nil	An instructor with extensive experience in both the practice and teaching of mindfulness	In-person group sessions. Setting not reported.	**Duration of intervention:** 7 weeks **Number of sessions:** 7 **Session frequency:** 1	None reported

		observing physical sensations of different body parts, observing thoughts); and (3) Exposure to and debate on metaphors through different stories and exercises used in Zen and Vipassana Buddhist meditation, which promote values such as acceptance, forgiveness, and non-identification with mental events. The program includes existential, ethical, and spiritual aspects and values, and thus conforms to the criteria for second generation mindfulness-based interventions. Each weekly session includes the following components: (1) Discussion and feedback on the mindfulness meditation exercises practiced during the previous week; (2) a 10-minute guided mindful body scan; (3) Presentation of the various metaphors and practicing exercises from Zen and Vipassana meditation; and (4) practicing full awareness of breathing for 30 minutes.	exercises daily at home for 10 minutes and 30 minutes, respectively.		meditation techniques.		session per week **Session length:** 2 hours	
Andrés-Rodríguez et al., 2019	MBSR + Usual Care	This study used the MBSR protocol developed at the University of Massachusetts Medical School. MBSR is an intensive, structured training program in mindfulness meditation, aiming to help participants increase their awareness of the present	Participants were encouraged to engage in 45 minutes of home mindfulness practice per day.	All study participants received usual care (see Appendix 3 for further details).	Five accredited MBSR instructors.	In-person group sessions. Setting not reported.	**Duration of intervention:** 8 weeks **Number of sessions:** 8x standard sessions + 1x optional silent retreat session	None reported

		experience and to relate to their physical and psychological conditions in a more accepting and nonjudgmental way. An MBSR book plus an audio CD with guided MBSR practices were provided to participants to facilitate home practice.					**Session frequency:** 1 standard session per week<br>**Session length:** Standard sessions – 2 hours; Optional silent retreat session – 6 hours	
Astin et al., 2003	Mind-Body Intervention	The intervention for this study consisted of two principal components: Mindfulness meditation training and Qigong. The first 90-minute period of each session was based closely on the MBSR program developed by Kabat-Zinn and colleagues at the University of Massachusetts Medical Center. As with the original program, these group sessions were centered on the principles and practice of mindfulness meditation, which involves the cultivation of moment-to-moment, nonjudgmental awareness of one's present-moment experience. The goal of this practice is to cultivate a stable and nonreactive awareness of one's internal (e.g., cognitive-affective-sensory) and external (social-environmental) experiences as contrasted with the tendency humans have to	Details not provided	In the last hour of each session, participants were introduced to the practice of Qigong. Qigong is an ancient Chinese health care system that integrates physical postures, breathing techniques, and focused intention. It is a system practiced for health maintenance, healing, and increasing	Not reported	In-person group sessions. Setting not reported.	**Duration of intervention:** 8 weeks<br>**Number of sessions:** 8<br>**Session frequency:** 1 session per week<br>**Session length:** 2.5 hours	None reported

		react quite reflexively (habitually or automatically) to the myriad situations and experiences (whether stressful or challenging, or not) encountered in daily life. Weekly didactic and experiential sessions involved the learning of two formal meditation practices (a body scan and sitting meditation) along with discussions about the practical applications of this type of mindful awareness for coping with pain, psychological distress, with the challenges of fibromyalgia, and with stressful life events in general.		vitality. The Qigong was taught by a Chinese master of this discipline using a specific form ("Dance of the Phoenix") that she developed specifically for patients with fibromyalgia.				
Bagheri et al., 2021	MBSR + Exercise Intervention	Participants received instruction on mindfulness meditation practices, such as breathing meditation, body scan meditation, gentle yoga, sitting meditation, and walking meditation. This instruction was expected to increase the awareness of thoughts, bodily sensations, and emotions, with an attitude of curiosity, openness, and acceptance. Participants were provided with training manuals and CDs with instructions for practice.	Participants were asked to practice the skills they learnt during group sessions at home for up to 45 minutes daily.	All study participants received an exercise protocol that consisted of 13 stretching, strength and balance exercises (see Appendix 3 for further details).	A trained sport psychologist.	In-person group sessions. Setting not reported.	**Duration of intervention:** 8 weeks **Number of sessions:** 8 **Session frequency:** 1 session per week **Session length:** Not reported	When a participant had difficulty learning any skill, the psychologist spent more time with that person at the end of the session as needed.
Banth & Ardebil, 2015	Modified MBSR + Usual Care	The program was based on Kabat-Zinn's MBSR program, with minor adaptations. Sessions involved explaining techniques, meditation practice, and feedback and	Participants were encouraged to practice one of the mindfulness meditation	All study participants could continue with their usual medical care, including	A psychologist.	In-person group sessions in a private psychiatry clinic.	**Duration of intervention:** 8 weeks **Number of sessions:** 8	None reported

		discussion of participants' experiences. The content of the eight sessions is described: (1) Welcome and introduction of the psychologist, ice breaker exercise, explanation of the conceptual framework and rationale behind MBSR for low back pain, explanation of the fundamentals of a mindfulness approach; (2) Explanation of the rationale behind mindfulness of breathing for reducing pain, education about two ways of practicing mindfulness of breathing (while formally sitting vs anywhere and at any time of day), practicing mindfulness of breathing; (3) Explanation of the rationale behind sitting meditation for reducing pain, practice of sitting meditation; (4) Explanation of the body scan technique and benefits in relation to low back pain, practice of body scan meditation; (5) Practice of advanced body scan; (6) Education about walking meditation and the rationale behind using walking meditation for low back pain, practice of walking meditation; (7) Education about how mindful living can reduce pain, mindful living exercises; (8) Acknowledgement, summary of sessions, opportunity for feedback. Participants were	techniques learnt during the group sessions, daily for 10-30 minutes.	physiotherapy sessions and medicine.			**Session frequency:** 1 session per week<br>**Session length:** 1.5 hours	

		provided with a brochure about mindfulness and the content of the eight sessions, and four CDs with guided meditation recordings.						
Brown & Jones, 2013	Mindfulness-Based Pain Management	The program taught participants not to try to do anything about the underlying unpleasant sensation of pain, but to train in mindfulness to lessen the reactive cycle that leads to physical and emotional stress. This was done by teaching breath awareness, body awareness, gentle movement, training in how to manage pain, illness and fatigue in daily life, and cultivating kindness and compassion toward oneself and others. The main components of the program were breath awareness, the body scan, mindful movement, mindfulness of daily life (pacing), three-minute breathing space, and kindly awareness.	Participants were encouraged to engage in 30 to 45 minutes of home mindfulness practice per day.	Nil	Not reported	In-person group sessions. Participants were allocated to 1 of 5 groups that were also open to self-referring individuals not involved in the study. There were up to 15 participants per group. Setting not reported.	**Duration of intervention:** 8 weeks **Number of sessions:** 8 **Session frequency:** 1 session per week **Session length:** 2.5 hours	None reported
Burns et al., 2022	MBSR	Included training in mindfulness through (1) body scan meditation, a gradual moving of attention through the body, accompanied by awareness of bodily sensations including sensations of breathing while in a lying position; (2) sitting meditation, focusing on awareness of breathing, bodily sensations, thoughts, and emotions, while	Details not provided	Nil	Postdoctoral level clinical psychologists with prior experience delivering psychosocial interventions for pain.	In-person individual sessions. Setting not reported.	**Duration of intervention:** 8 weeks **Number of sessions:** 8 **Session frequency:** 1 session per week **Session length:** 1.5 hours	None reported

		sitting on a chair or cushion; and (3) gentle movement exercises intended to develop awareness (mindfulness) during movement. Each session included practice of one or more of these techniques. In-class didactic material and discussion of participants' experiences of developing and applying mindfulness in everyday life were also part of each session. In-class activities included suggestions for application of mindfulness as a method for responding positively to stress, dealing with the challenges of pain, and exercises focusing on the challenges and achievements participants experienced in integrating mindfulness into their lives and the stressful situations they encountered. Additional discussion focused on stress reactivity. Format of sessions included: (1) review of previous week's session, (2) homework review, (3) session treatment objectives, and (4) assign homework. Participants were provided with handouts.						
Cash et al., 2015	MBSR	The intervention followed the traditional MBSR program. Both formal and informal mindfulness practices were introduced. Formal meditations included an attention-focusing technique	Participants were encouraged to practice the three formal meditation techniques for	Nil	An experienced, trained MBSR instructor who had completed the professional internship training program	In-person group sessions. Setting not reported.	**Duration of intervention:** 8 weeks **Number of sessions:** 8x standard sessions + 1x	None reported

		(body scan, directing attention throughout the body in a relaxed, supine state), sitting meditation (systematically directing attention to breath and immediate sensory and cognitive experiences), and a series of simple yoga positions taught as a means of encouraging relaxed and focused movement. Participants were provided with a workbook and audiotapes which guided the home practice assignments.	45 minutes per day, six days a week.		at the University of Massachusetts Medical Center Stress Reduction Clinic.		silent retreat session **Session frequency:** 1 standard session per week **Session length:** Standard sessions – 2.5 hours; Silent retreat session – Half day	
Chaharmahali et al., 2023	Mindfulness + Neuromuscular Exercises	Mindfulness was implemented using breathing and attention exercises. The content of the six sessions is described: (1) Introduction to breathing intervention; (2) Constantly focusing on the breath, understanding the inhale and exhale, fully performing breathing, paying attention to the muscles from contraction to relaxation, being aware of the body position without judgement, giving thanks for the positive events of the day; (3) Continuing the previous session's exercise with fewer verbal instructions, focusing on the flow of breathing while paying attention to the muscles involved, nonjudgemental awareness of the body's condition, gratitude for the positive events of the day; (4) Repetition of key principles,	Details not provided	All study participants completed neuromuscular exercises performed under the supervision of a certified trainer (see Appendix 3 for further details).	A clinical psychologist.	Online live individual sessions.	**Duration of intervention:** 6 weeks **Number of sessions:** 6 **Session frequency:** 1 session per week **Session length:** Initial session – 45-60 minutes; Subsequent sessions – 15-20 minutes	None reported

		practice of meditation listening to distant and then near sounds; (5) Limited instructions, the participant was encouraged to perform the meditation exercise alone while listening to distant and nearby sounds, then paying attention to the sound of their breathing and the sounds of their mind; (6) Participants encouraged to practice the meditation alone while listening to nearby and distant sounds, then paying attention to the sound of their breathing and the sounds of their mind, giving thanks for the positive events of the day. Participants were provided with a video and audio file containing mindfulness exercises (conscious breathing meditations) and a notebook for recording gratitudes and bringing attention to positive events in daily life.						
Chen et al., 2021	MBSR + Intensive Education Program	Sessions 1 and 2 focused on mindful breathing. Participants were informed of the concept, background, and benefits of mindfulness meditation practice, informed of the intervention process, and given electronic manuals and videos which demonstrated the methods and procedures of mindful breathing. "Mindful breathing workshops" were established through a social	Home practices were prescribed. Further details were not reported.	All study participants received routine care and an eight-week intensive education program (see Appendix 3 for further details).	Not reported	In-person group sessions. Setting not reported.	**Duration of intervention:** 8 weeks **Number of sessions:** 8 **Session frequency:** 1 session per week **Session length:** Not reported	None reported

		media platform. Participants were actively encouraged to practice the skills and techniques of mindful breathing, and their concerns were addressed. Sessions 3 and 4 focused on body scanning. The body scan meditation involved paying attention to the physical sensations in the body. The participants were assigned practice tasks and asked to record any problems they encountered during the meditation exercises. Sessions 5 and 6 focused on mindful yoga. Sessions 7 and 8 focused on mindfulness meditation more broadly. Through the WeChat platform, questions about the assigned tasks and practices were answered.						
Cherkin et al., 2016	MBSR + Usual Care	The MBSR intervention was modeled closely after the original MBSR program. It did not focus specifically on a particular condition such as pain. All classes included didactic content and mindfulness practice (body scan, yoga, meditation [attention to thoughts, emotions, and sensations in the present moment without trying to change them, sitting meditation with awareness of breathing, and walking meditation]). Methods to challenge dysfunctional	Participants were instructed to practice the techniques discussed in each session daily for up to 45 minutes, throughout the intervention period and after classes ended. They were also assigned readings to	All study participants continued to receive their usual medical care throughout the study.	Eight instructors with 5 to 29 years of MBSR experience. Six of the instructors received training from the Center for Mindfulness at the University of Massachusetts Medical School.	In-person group sessions in large, quiet classrooms at Group Health facilities that are centrally located and that provide easy access for persons with physical limitations.	**Duration of intervention:** 8 weeks **Number of sessions:** 8x standard sessions + 1x silent retreat session **Session frequency:** 1 standard session per week **Session length:** Standard	None reported

		thoughts were proscribed in the MBSR intervention. A description of the eight sessions is provided: (1) Setting expectations, definition of mindfulness, mindfulness exercise, movement, abdominal breathing instruction, introduction to home practice; (2) Perception and responding creatively to perceptions, yoga, body scan, discussion of developing a practice; (3) The pleasure and power of being present, yoga, walking meditation, how to bring more pleasant events into our lives; (4) Getting unstuck from old patterns, yoga/movement, sitting meditation, stress (define, identify, how and why we stay stuck), dealing with pain; (5) Reacting and responding differently to stress, yoga, guided meditation, establishing coping strategies (living with difficulties); (6) Learning about communication patterns, yoga/meditation, styles of communicating with others (effective and ineffective); (7) Integrating the learning from the techniques, yoga/meditation, practical ways to bring mindfulness into daily life; (8) This is the rest of your life, review of mindfulness techniques/applications, closure. An optional six-hour	complete for each session.				sessions – 2 hours; Silent retreat session – 6 hours	

		"Day of Practice" retreat was offered. The retreat was held in silence, with only the instructor speaking. This retreat provided participants an opportunity to deepen what they learned in class. Participants were provided with a workbook and audio CD.						
Dalili & Bayazi, 2019	Modified MBCT	The intervention was based on that of Segal et al, with minor modifications made. The structure of each session included: Reviewing homework assignments, reviewing the previous session, presenting the first subject and practicing, resting and welcoming, presenting the second subject and exercising, summarizing and presenting the homework assignments of the next week. Session-by-session content is described: (1) Awareness of mindfulness and expression of logic; (2) Teaching and practicing mindfulness techniques with breathing awareness to increase attention and concentration capacity, including 45-minute body scan and 10 minute breathing exercise; (3) Performing a technique of slowing muscular tone, exercising vigilance and widespread keeping of the mind through the pursuit of meditative exercises and focusing on conscious	Home practices were prescribed. Further details were not reported.	Nil	Not reported	In-person group sessions. Setting not reported.	**Duration of intervention:** 8 weeks<br>**Number of sessions:** 8<br>**Session frequency:** 1 session per week<br>**Session length:** 2 hours	None reported

		breathing, practice of a mind-boggling technique and 3-minute breathing exercise; (4) Creating acceptance and mindfulness by abandoning attempts to control and cognitive failure; (5) A 45-minute session of a meditating technique, expressing logic, a 3-minute breathing exercise, and providing simulated exercises for use in times of emotion, understanding Beck's model of anxiety and depression based on the ABC model; (6) 45-minute sitting meditation, exploring emotions and excitement, teaching techniques to respond to negative thoughts, 3-minute conscious breathing exercise; (7) Performing meditation exercises, listing pleasurable and skillful signs and exercises for the body; (8) Summarizing and reviewing the program, applying lessons for dealing with the future.						
Davis et al., 2013	Mindful Socioemotional Regulation Intervention	A mindfulness-based intervention for emotion regulation for people with chronic pain. Designed to address the positive emotional and social engagement deficits evident in fibromyalgia. Specifically, the training focused on (1) the regulation of emotions via enhancing awareness and acceptance of the full range of emotion	Each week participants were prescribed a mindfulness meditation exercise to practice daily.	Nil	Not applicable	Online asynchronous individual sessions.	**Duration of intervention:** 6 weeks<br>**Number of sessions:** 12<br>**Session frequency:** 2 sessions per week<br>**Session length:** 15 minutes	None reported

		experiences via mindfulness meditation; and (2) the use of mindful awareness skills to make choices that build stronger social bonds, enhancing a sense of belonging and increasing enjoyment of social relations. The program was comprised of 12 modules, each lasting approximately 15 minutes and centering on a particular topic: (1) Welcome and introduction to mindfulness; (2) Positive and negative emotions: two dimensions; (3) Mindfulness of emotions; (4) Awareness of emotions and pain; (5) Acceptance of emotions; (6) Mindful living with pain; (7) Pacing yourself mindfully; (8) Emotions and thoughts; (9) Thoughts and beliefs; (10) Savoring the positive; (11) Building mindful relationships; and (12) Bringing it all together. The material in the modules was delivered via Adobe Presenter, which allowed visual presentation of bulleted text and animated pictures that accompanied an audio recording of module content. Embedded within each module were brief activities designed to help participants reflect on how a concept applied to their current situation. Participants could pause the						

		session, rewind to review material, or skip ahead if they wished. At the end of each module, participants were: (1) encouraged to use the skills discussed in the module over the next several days; and (2) provided with a link to an audio recording of a mindfulness meditation relevant to the material covered in the module, and encouraged to access the meditation daily.						
Day et al., 2019	1 – Mindfulness Meditation (MM)<br><br>2 – MBCT	1 – MM: Adapted from MBCT, with all cognitive-behavioral and mindful movement components removed. Participants were trained in a series of formal mindfulness meditation (Vipassana) practices (e.g., body scan, breath-focused meditation). A standard script was used to guide participants in the meditation practices. Time was devoted each session to a guided inquiry of participants' experiences. Participants were also taught a brief, three-minute breathing space meditation. Participants were provided with 20-minute and 45-minute recordings of the meditation techniques taught in the sessions, to support home practice. Brief weekly reading materials and/or handouts were also provided.	All participants were instructed to practice the pain management skills taught in between the group sessions for 45 minutes, six days per week.<br><br>1 – MM: Participants were encouraged to practice first using the recordings and then later on their own without recordings. Participants were also encouraged to	Nil	A licensed clinical psychologist with extensive experience in delivering the treatments under investigation and who has maintained a personal, long-term mindfulness meditation practice, plus an observing co-therapist, who was a Master's-level graduate student enrolled in the University of Queensland clinical/applied psychology graduate program who assisted with	In-person group sessions held at the University of Queensland Psychology Clinic.	**Duration of intervention:** 8 weeks<br>**Number of sessions:** 8<br>**Session frequency:** 1 session per week<br>**Session length:** 2 hours	None reported

		2 – MBCT: Integrated cognitive and behavioral techniques with mindfulness-based strategies. The cognitive-behavioral exercises were designed to enhance awareness of the stress-pain connection and the role of unhelpful automatic thoughts, and of scheduling in nourishing, pleasant activities for pain management. Simultaneously, the mindfulness exercises trained the mind to step out of automatic patterns of thinking, feeling and reacting to pain, to nonjudgmentally accept experience, and to purposefully, with kindness and a nonjudgmental attitude, place attention on anchoring bodily sensations (e.g., the breath) and other perceptive experiences (e.g., sounds). Each session included experiential practice with various Vipassana techniques, followed by a guided inquiry, as well as cognitive exercises. Guided meditation audio recordings and weekly reading materials were provided.	use the three-minute breathing space exercise at least three times per day between sessions.<br><br>2 – MBCT: Included guided meditation practice with audio recordings (including a guided mindful movement and walking practice, starting mid-treatment), the three-minute breathing space practice (three times per day), brief cognitive-behavioral exercises, and weekly reading materials.		session logistics (e.g., escorting participants from the car park, collecting paperwork).			
Day et al., 2024	Mindfulness Meditation	The intervention was based on a standardized protocol and materials that were developed by the author team and refined in prior trials. The therapists were expected to follow the treatment manual closely to ensure all content was	Participants were assigned additional readings and homework assignments to complete between	Nil	Five therapists who were post-doctoral psychology fellows or licensed psychologists	Online live group sessions.	**Duration of intervention:** 4 weeks<br>**Number of sessions:** 8<br>**Session frequency:** 2	None reported

		delivered and to ensure the consistency and replicability of treatment. Mindfulness meditation participants were trained in a combination of Shamatha meditation, which involves training the mind for stability in maintaining focus on a specific object, and Vipassana, an open monitoring practice which involves acknowledging any sensory, emotional, or cognitive event that arises in the mind without evaluation, interpretation, or preference. Participants were taught the following mindfulness meditation practices: (1) 3-minute breathing space; (2) body scan meditation; (3) mindfulness of the breath and body practice; (4) mindfulness of the breath, body, sounds, and thoughts; and (5) mindfulness of the breath, body, sounds, and thoughts while also intentionally working with a difficulty through this practice. A guided inquiry of the participant's experiences followed each in-session exercise and was also implemented in relation to discussing participants' at home practice. Participants were provided with workbooks.	sessions. Participants assigned to the mindfulness intervention also received audio-recorded guided meditations to facilitate home practice.		with at least two years of clinical experience, including experience providing psychological treatments for chronic pain. Therapist training consisted of assigned readings and a 6-hour workshop as well as a 3-hour motivational interviewing training for enhancing motivation to engage in treatment (i.e., via enhancing reflective listening practices), and a 3-hour training in group leadership techniques and conducting telehealth sessions, including strategies for enhancing group cohesion. Group supervision was provided weekly		sessions per week **Session length:** 1.5 hours	

					during treatment delivery to support fidelity.			
de Oliveira Melchior et al., 2025	Mindfulness Program	Comprised practices aligned with the principles of MBSR and the recommendations of the United Kingdom Network for Mindfulness-Based Teachers Good Practice Guidelines for Teaching Mindfulness-Based Courses (2015). A summary of the content covered in the eight sessions is provided: 1) 'Change is easy' - Overview of the program, participant taught that they have the tools necessary to be successful in the practice of mindfulness and that practicing mindfulness is natural and innate; 2) 'Breathing is natural' - Expansion of perception, the breath represents the central focus of the experience, spaces are opened for the emphasis on feeling and the reduction of verbalization; 3) 'Keeping the body conscious' - Re-establishing the connection between bodily sensations and mental/cognitive processes; 4) 'Just be' - Recognition and acceptance of the present moment; 5) 'Moment by moment' - Focus of attention expanded to perceive the constant impermanence of mental and emotional states, gradual recognition of	Participants were instructed to practice the exercises taught during the weekly in-person sessions daily on the remaining days of the week.	Nil	Certified instructors from the Center for Mindfulness and Integrative Therapies (CEMITI) at Ribeirão Preto Nursing School, University of São Paulo (EERP/USP), Brazil.	In-person group sessions with up to 10 participants per group. Sessions were conducted in a calm, quiet, spacious and well-ventilated environment that included chairs and cushions, and a floor covered with a 3cm thick rubber mat, allowing participants to choose where to sit comfortably.	**Duration of intervention:** 8 weeks **Number of sessions:** 8 **Session frequency:** 1 session per week **Session length:** 2 hours	None reported

		transience fostering the idea of detachment; 6) 'Welcoming emotions' - Skills to enhance inner coping resources and support resilience, accepting difficult emotions, recognizing the changing nature of emotions and associated physical sensations; 7) 'Living mindfulness' - Recognizing the challenges to practicing mindfulness in daily life and exploring ways to integrate mindfulness; 8) 'Cultivating peace' - Reflection on experiences and lessons learned, planning of strategies to encourage sustained practice. Participants were provided with printed materials with the content covered in each session, written in informal and easy-to-understand language, audio recordings to assist in meditation practices available via email, WhatsApp and CD, according to their preference, and a diary to record practices to encourage the incorporation of practices into participants' daily lives.						
Diez et al., 2022	MBSR for Chronic Back Pain	Based on the MBSR program proposed by the Mindfulness Center of the University of Massachusetts, but with the following modifications to better accommodate individuals with chronic back pain: 1) Two sessions related to	Participants were instructed to follow a series of daily 45 minute exercises which were specifically	Nil	An instructor with 10 years of experience teaching MBSR who was trained at the Center for Mindfulness at the University of	In-person group sessions. Setting not reported.	**Duration of intervention:** 8 weeks **Number of sessions:** 8x standard sessions + 1x	None reported

		body postures were replaced by muscle relaxation and simpler movements supervised by two physiotherapists - the purpose of this modification was to avoid unnecessary risks for participants by performing postures that could be a biomechanical challenge, to encourage mobility by starting with postures that would provide greater control and safety, and to facilitate the development of mindfulness in the interoception of posture; and 2) The inclusion of psychoeducational contents on pain, the role of psychological distress in the development of pain, and the use of effective coping strategies, with a focus on tolerance and acceptance. Participants were given access to an online platform with guided meditation audio recordings to support home practice, and a course workbook.	tailored for back pain conditions.		Massachusetts Medical School.		silent retreat session **Session frequency:** 1 standard session per week **Session length:** Standard sessions – 2.5 hours; Silent retreat session – 7.5 hours	
Donnino et al., 2021	MBSR	This program was modeled after the MBSR Authorized Curriculum Guide 2017 published by the University of Massachusetts Medical School, which established MBSR in 1979.	Details not provided	Nil	Instructors with formal training in mindfulness-based interventions with experience ranging from 3-24 years.	In-person group sessions. Setting not reported.	**Duration of intervention:** 8 weeks **Number of sessions:** 8x standard sessions + 1x silent retreat session **Session frequency:** 1 standard	None reported

							session per week<br>**Session length:** Standard sessions – 2 hours; Silent retreat session – 6 hours	
Farzadkia et al., 2023	MBSR	Based on the MBSR program of Kabat Zinn. A summary of the content covered in the eight sessions is provided: (1) Introducing the automatic guidance system, using present-moment awareness of bodily sensations, thoughts, and emotions to reduce stress, the Raisin Exercise, giving feedback and discussing exercise, the three-minute breathing space; (2) Repeating the practice of body scan, giving feedback and discussing this practice, mindfulness meditation breathing exercise, yoga stretching exercise; (3) Sitting mindfully with awareness of breathing (sitting meditation), yoga exercises, the three-minute breathing exercise; (4) Repeating the body scan exercise, mindfulness yoga exercises, conducting five-minute "seeing or hearing" exercises, repeating mindful sitting with breath and body awareness; (5) Doing breathing exercises, repeating mindful sitting	Details not provided	Nil	A study investigator who had attended specialized courses and workshops.	In-person individual sessions. Setting not reported.	**Duration of intervention:** 8 weeks<br>**Number of sessions:** 8<br>**Session frequency:** 1 session per week<br>**Session length:** 2 hours	None reported

		(awareness of breath, body, sounds, thoughts), explanations regarding stress and determining participants' reactions to stress, examining awareness of the effect of pleasant and unpleasant events on feelings, thoughts, and bodily sensations, mindful yoga exercises, the three-minute breathing space exercise; (6) Doing mindful yoga, sitting meditation (awareness of sounds and thoughts); (7) Conducting mountain meditation, repeating exercises from previous sessions, preparing a list of enjoyable activities; (8) Doing the body scan exercise, reviewing the entire program, examining and discussing the program, doing stone, bead, and marble meditation.						
Fogarty et al., 2019	MBSR + Usual Care	In each session, participants practiced mindfulness-based exercises and participated in discussions on a specific topic related to living with chronic illness (e.g., coping with pain and stress). Through meditative practices including awareness of breathing, mindful eating, body scan meditation, walking meditation, gentle yoga (tailored to accommodate patients with rheumatoid arthritis), loving-kindness	Participants were instructed to practice at home for 30 minutes per day, six days per week, with specific practices assigned for each week.	All study participants received usual care (see Appendix 3 for further details).	A facilitator who trained through the Center for Mindfulness at the University of Massachusetts Medical School.	In-person group sessions. Setting not reported.	**Duration of intervention:** 8 weeks **Number of sessions:** 8x standard sessions + 1x silent retreat session **Session frequency:** 1 standard session per week	None reported

		meditation, and mindful speaking and listening, participants were encouraged to become aware of, and intentionally respond to, their bodily sensations, thoughts, and emotions. At the initial session, participants were provided with a workbook and a set of CDs with guided instructions for a specific type of mindfulness exercise.					**Session length:** Standard sessions – 2 hours; Silent retreat session – Full day	
Liu et al., 2025	Mindfulness-Based Therapy + Usual Care	Consisted of 10 components: 1) Engaging in meditation training, including breathing, mindfulness, relaxation, and body scanning meditations; 2) Overcoming distractions; 3) Focusing on coping with unusual feelings; 4) Developing patience and embracing process; 5) Calming tension; 6) Identifying worries, including interpreting pleasant and unpleasant experiences, recalling associated thoughts and feelings, and adopting alternative perspectives; 7) Reducing worries, including shifting away from complex thoughts, listing positive activities, and making conscious choices; 8) Soothing the body, including promoting a sense of wholeness, acknowledging personal limitations, and finding meaning beyond illness; 9) Pain control, including the	Details not provided	All participants received usual care; either conservative management or surgical management. Conservative management involved activity restrictions, oral non-steroidal anti-inflammatory drugs, and physiotherapy exercises emphasizing shoulder flexion, external rotation, and extension functions. Surgical approaches	The mindfulness training was administered by professionally trained practitioners who completed a standardized mindfulness therapy training program (including theoretical modules and practical supervision) and passed pre-study simulation assessments. All interventionists held backgrounds in psychology or medicine and underwent 20 hours of study-specific training covering protocol	Not reported	**Duration of intervention:** 8 weeks **Number of sessions:** 8 **Session frequency:** 1 session per week **Session length:** 50 minutes	None reported

		distinction between the sensation of pain and the emotional response to it, and developing coping strategies; and 10) Integrating mindfulness into daily life, including finding moments of quiet, appreciating the beauty of nature, and discovering the wonders of the moment.		involved arthroscopic procedures, including capsular release, middle glenohumeral ligament release, subscapularis release, and synovial membrane clearance.	procedures, standardized scripting, and contingency management.			
Marais et al., 2022	MBSR + Usual Care	The MBSR program included training in mindfulness through: (1) a body scan, the gradual moving of attention through the body from head to feet while lying on a mat on the floor, bringing awareness particularly to bodily sensations; (2) sitting meditation, in which attention is brought to breathing sensations and the flow of bodily sensations, sounds, thoughts, and emotions; and (3) mindful stretching exercises, to cultivate awareness during simple stretching movement. The key themes of the MBSR program included the empowerment of participants and a focus on awareness and acceptance of experience of the present moment. The MBSR program did not focus specifically on a particular condition such as	The program included 45 minutes of daily homework exercises that consisted of guided or unguided awareness exercises directed at increasing moment-by-moment, nonjudgmental awareness of bodily sensations, thoughts and feelings, together with exercises designed to integrate the application of	All study participants received usual care (see Appendix 3 for further details).	An instructor (physician and psychotherapist) who trained at the Association pour le Développement de la Mindfulness (French representative of the Mindfulness Center at Brown University, USA), attended silent retreats and had more than three years of experience teaching mindfulness-based interventions.	In-person group sessions. Setting not reported.	**Duration of intervention:** 8 weeks<br>**Number of sessions:** 8<br>**Session frequency:** 1 session per week<br>**Session length:** 2.5 hours	None reported

		pain. Participants were provided with an MP3 recording of guided awareness exercises.	awareness skills into daily life.					
Mohammadi et al., 2011	Modified MBCT	Participants in the intervention group underwent MBCT focused on pain control. This program, designed by researchers, was based on the principles of mindfulness to prevent depression relapse and incorporated cognitive-behavioral techniques for chronic pain management.	Home practices were prescribed. Further details were not reported.	Nil	Not reported	In-person group sessions. Setting not reported.	**Duration of intervention:** 8 weeks **Number of sessions:** 8 **Session frequency:** 1 session per week **Session length:** 2 hours	None reported
Morone et al., 2008	Mindfulness Meditation Program	The intervention was modelled on Jon Kabat-Zinn's MBSR program. Three techniques of mindfulness meditation were taught: (1) the body scan, where in a lying position, the participant is guided to place their attention nonjudgmentally on each area of the body from the toes to the top of the head; (2) sitting practice, which is focused attention on breathing while sitting on a chair or on a meditation cushion on the floor; and (3) walking meditation, which is mindful slow walking with focused attention on body sensation and/or breathing. Several attitudes that support mindfulness meditation were emphasized throughout the eight classes. These were patience, nonjudging,	Participants were encouraged to complete 45 minutes meditating and 5 minutes completing a diary entry, on six days per week.	Nil	Two study investigators who had each undergone teacher training in an MBSR program for health professionals directed by staff from the Center for Mindfulness, and who had a combined experience of 30 years in mindfulness meditation.	In-person group sessions. Setting not reported.	**Duration of intervention:** 8 weeks **Number of sessions:** 8 **Session frequency:** 1 session per week **Session length:** 1.5 hours	None reported

		beginner's mind, acceptance, letting go, non-striving and trust. We did not include a yoga component or an all-day silent retreat that are usual components of an MBSR program. Participants were provided with an audiotape, daily diary and reading materials. The audiotape was a guided 45-minute recording of the steps in the body scan meditation on one side and a guided 30-minute recording of sitting meditation on the other side.						
Morone et al., 2009	Mindfulness Meditation Program	The intervention was modelled on Jon Kabat-Zinn's MBSR program. Three techniques of mindfulness meditation were taught. These techniques take regular activities like sitting, walking, and lying down and transform them into a meditation through directed breathing and mindful awareness of thoughts and sensations. The methods used were 1) the body scan, where in a lying position, the participant is guided to place their attention nonjudgmentally on each area of the body from the toes to the top of the head; 2) sitting practice, where the participant is guided to focus their attention on breathing while sitting on a chair; and 3) walking meditation, where the	Participants were asked to practice at home 45 minutes per day, 6 days per week.	Nil	Instructors had at least 50 hours of training in the MBSR program and a personal mindfulness meditation practice of over 25 years.	In-person group sessions. Setting not reported.	**Duration of intervention:** 8 weeks<br>**Number of sessions:** 8<br>**Session frequency:** 1 session per week<br>**Session length:** 1.5 hours	None reported

		participant is guided in mindful slow walking with focused attention on body sensation and/or breathing. Participants also engaged in activities such as a mindful eating exercise and informal mindfulness practices. If physical discomfort arose during any meditation participants were encouraged to change to a more comfortable position. Participants were also introduced to pain theory and the multidimensional response to pain. Participants learned about the cognitive, emotional, and sensory components of nociceptive processing. How to use mindfulness meditation methods to work with pain was discussed. Each session included one hour (total) of meditation and 30 minutes of discussion and reflection on participants' experiences. Participants were provided a CD recording of a 45 minute guided body scan meditation and 30 minute guided sitting meditation, home practice log, and reading materials. They were provided with a CD player if they did not have one.						
Morone et al., 2016	Mind-Body Program	The intervention was modelled on the 8-week MBSR program. Four methods of mindfulness meditation were taught. These techniques take regular activities such as sitting,	Details not provided	Nil	Not reported	In-person group sessions. Setting not reported.	**Duration of intervention:** 8 weeks<br>**Number of sessions:** 8	None reported

		walking, and lying down and transform them into a meditation through directed breathing and mindful awareness of thoughts and sensations. The methods used included the body scan, sitting practice, walking meditation, and mindful stretching.					**Session frequency:** 1 session per week **Session length:** 1.5 hours	
Pal et al., 2023	Modified MBSR	An adapted version of the MBSR program - adaptations were made to enable a brief version of the program to be delivered in a hospital outpatient setting and aimed to retain maximum participants with minimal dropouts. Session focuses included: (1) introduction to mindfulness, (2) mindfulness and chronic pain, (3) application of mindfulness to deal with pain catastrophizing and to increase chronic pain acceptance, (4) interrupting the cycle of sufferings; moving forward with acceptance and self-compassion, and (5) sum up of all learnings. In-session mindfulness practices included mindfulness of breathing, body scan, mindfulness of pain areas, and self-compassion practice.	Participants were encouraged to perform 20 minutes of daily home practice.	Nil	The mindfulness instructor was trained via the original MBSR program and ‘mindfulness for managing pain’ program and had an experience of teaching mindfulness for more than 3 years.	In-person group sessions. Setting not reported.	**Duration of intervention:** 5 weeks **Number of sessions:** 5 **Session frequency:** 1 session per week **Session length:** 1.5 hours	None reported
Pal et al., 2024	Modified MBSR	A five-session version of the MBSR program was chosen. Session details are provided: (1) Introduction to mindfulness practice, mindfulness of breathing; (2) Mindfulness and	Participants were encouraged to perform daily home practice	Nil	The program was conducted by a pain physician trained via the original MBSR	In-person group sessions. Setting not reported.	**Duration of intervention:** 5 weeks **Number of sessions:** 5	None reported

		use in chronic low back pain, body scan practice; (3) Application of mindfulness to deal with pain catastrophizing and to increase chronic pain acceptance, mindfulness of pain areas meditation; (4) Interrupting the cycle of pain to suffering: moving forwards with acceptance and self-compassion, self-compassion practice; (5) Sum up of all learnings, review of all meditation practices.	of meditation for 20 minutes.		program with 4 years of teaching experience.		**Session frequency:** 1 session per week **Session length:** 1.5 hours	
Parra-Delgado & Latorre-Postigo, 2023	Modified MBCT + Usual Care	Different practical mindfulness exercises were conducted at each of the sessions, with special focus on pain-related stimuli. The main aim was for participants to learn mindfulness techniques in order to relate to their experience of pain and the thoughts and feelings it provokes in a different way, responding in a compassionate and nonjudgmental way. The participants were invited to reflect on the transitory nature of the different painful stimuli and were invited to experience their thoughts as passing events of the mind rather than absolute truths. Psycho-educational activities on the causes and development of the symptoms of depression and anxiety were also conducted. In the final sessions the participants were invited to	The participants carried out formal practice at home (i.e., body scan, sitting meditation, walking meditation or mindful breathing) 6 days a week.	All study participants continued with their usual medication treatment, medical visits, rehabilitation sessions and activities proposed by the Fibromyalgia Association.	The sessions were administered by a therapist with practical and theoretical certified training in MBCT.	In-person group sessions. Setting not reported.	**Duration of intervention:** 8 weeks **Number of sessions:** 8 **Session frequency:** 1 session per week **Session length:** 2.5 hours	None reported

		identify simple methods of self-care and to use the skills learnt to respond to future mood states and/or painful body experiences. The modifications to the MBCT program for the women with fibromyalgia were: (1) a closer look at the acceptance of the experience of pain in the different meditation practices of mindfulness; (2) encouraging participants to be aware of the automatic thoughts related to the response to pain and their relationship to the feelings and behaviors it caused; (3) providing information on anxiety and its causes; (4) explaining the importance of not forcing their body into yoga postures and of feeling comfortable by using appropriate clothes and postures during the practice of mindfulness.						
Pérez-Aranda et al., 2019	MBSR + Usual Care	We used the MBSR protocol developed at the University of Massachusetts Medical School with minimal adaptations for participants with fibromyalgia. A variety of meditation exercises were practiced during treatment sessions (e.g., Hatha yoga, sitting and walking meditation, body scan). Home practice experiences were reviewed, discussed, and reinforced during treatment	Homework exercises were prescribed each week.	All study participants continued with their regular pattern of medication if any.	Five properly trained MBSR instructors.	In-person group sessions. Setting not reported.	**Duration of intervention:** 8 weeks **Number of sessions:** 8x standard sessions + 1x optional silent retreat session **Session frequency:** 1 standard	None reported

		sessions throughout the program. Audiotapes were provided to facilitate practice at home.					session per week<br>**Session length:** Standard sessions – 2 hours; Optional silent retreat session – 6 hours	
Plews-Ogan et al., 2005	MBSR	Based on Kabat-Zinn's program. A mind-body intervention involving the practice of meditation and yoga techniques to foster mindfulness (present moment, nonjudgmental awareness). Participants were provided with audiotaped meditation exercises.	Audiotaped meditation exercises were assigned as daily home practice. Participants were encouraged to use these skills in moments of stress or pain.	Nil	Not reported	In-person group sessions. Setting not reported.	**Duration of intervention:** 8 weeks<br>**Number of sessions:** 8<br>**Session frequency:** 1 session per week<br>**Session length:** 2.5 hours	None reported
Pradhan et al., 2007	MBSR	Classes consisted of conceptual training in mindfulness, discussions of its application in daily life, and experiential training in meditation and gentle yoga. Meditation was introduced in four formats: sitting meditation using the breath as an anchor of attention, sitting meditation characterized by a state of open awareness, progressive body relaxation meditation, and contemplative walking. Yoga poses were tailored to accommodate patients with rheumatoid arthritis. Standard MBSR	Participants were asked to practice at home 45 minutes a day, 6 days a week.	Nil	Three certified MBSR teachers taught the classes, all trained through the Center for Mindfulness at the University of Massachusetts Medical School.	In-person group sessions. Setting not reported.	**Duration of intervention:** 8 weeks<br>**Number of sessions:** 8x standard sessions + 1x silent retreat session<br>**Session frequency:** 1 standard session per week<br>**Session length:** Standard sessions – 2.5	None reported

		course materials were used. Participants were provided with an audio CD to support home practice.					hours; Silent retreat session – Full day	
Reiner et al., 2019	Mindfulness Meditation Program + Usual Care	The mindfulness program was inspired by Kabat-Zinn's MBSR program and Segal et al.'s MBCT program. Each session included familiarization with and practice of mindfulness meditation practices including breathing meditation, body scan, open awareness meditation, and walking meditation, as well as group discussions focused on mindfulness principles, participants' difficulties around practice, and the association between practice and pain coping. Audio CDs with meditation instructions were provided to facilitate home practice.	Participants were encouraged to practice meditation daily for at least 20 minutes.	All study participants continued to receive usual care (see Appendix 3 for further details).	A trained mindfulness meditation instructor with over 10 years of mindfulness meditation experience was responsible for administering the program.	In-person group sessions. Setting not reported.	**Duration of intervention:** 8 weeks<br>**Number of sessions:** 8<br>**Session frequency:** 1 session per week<br>**Session length:** 1.5 hours	None reported
Robles et al., 2025	Modified MBSR	The traditional MBSR program was adapted for chronic back pain. Pain coping skills training and sequential body movements were included. The sessions included theoretical and practical contents, in addition to detailed explanation of the exercises that participants were prescribed to complete at home. The two sessions related to body postures, originally proposed by Kabat-Zinn, were replaced by muscle relaxation and simpler	As homework, participants were instructed to follow a series of daily 45-minute mindfulness exercises which were specifically tailored for back pain conditions.	Nil	Qualified professionals who are certified instructors by the University of Massachusetts Medical Center, where the MBSR program was developed.	In-person group sessions. Setting not reported.	**Duration of intervention:** 8 weeks<br>**Number of sessions:** 8x standard sessions + 1x silent retreat session<br>**Session frequency:** 1 standard session per week<br>**Session length:**	None reported

		movements supervised by two specialists in physiotherapy. The purpose of this modification was to avoid unnecessary risks for participants by performing postures that could be a biomechanical challenge, to encourage mobility by starting from postures that would provide greater control and safety and to facilitate the development of mindfulness in the interoception of posture. Another modification to the MBSR program was the inclusion of psychoeducational contents on pain, the role of psychological distress in the development of pain and the use of effective coping strategies, with a focus on tolerance and acceptance. Thus, the adapted program paid more attention to the role of interoception and proprioception in the development of mindfulness. Participants were provided with an online platform where they had access to audio recordings for the daily homework exercises, a workbook and access to the instructor to solve doubts related to the program.					Standard sessions – 2.5 hours; Silent retreat session – 7.5 hours	
Safavi-Farokhi et al., 2020	MBCT + Stabilizing Exercises	Each session started with a review of the previous week's assignment and ended with presentation of homework	Home practices were prescribed. Further details	All participants were prescribed stability	A psychologist	In-person group sessions.	**Duration of intervention:** 4 weeks	None reported

		assignments for the coming week. The first four sessions were primarily focused on making the participants more aware of their environments and learning how to act mindfully. In sessions 5 to 8 participants learn how to be more aware of mood and how to manage this and focus on the importance of returning to the present moment with the help of breathing.	were not reported.	exercises aimed at strengthening the deep trunk muscles, with a particular emphasis on the lumbar multifidus (see Appendix 3 for further details).		Setting not reported.	**Number of sessions:** 8 **Session frequency:** 2 sessions per week **Session length:** 2 hours	
Sannigrahi et al., 2022	Mindfulness-Based Meditation + Balance Exercises	The mindfulness intervention included an introductory presentation and discussion, brief personal interview, personal instruction meeting, and three follow-up small group seminars over the course of one week. Each participant was then asked to practice mindfulness-based meditation for 20 minutes daily for three months. They were followed regularly through mobile and when possible, with contact sessions.	20 minutes of mindfulness meditation daily.	All study participants engaged in a balance program (see Appendix 3 for further details).	Not reported	Mix of in-person group and individual sessions + virtual individual sessions. Setting not reported.	**Duration of intervention:** 3 months **Number of sessions:** 4x in-person sessions; Number of virtual sessions not reported **Session frequency:** Not reported **Session length:** Not reported	None reported
Schmidt et al., 2011	MBSR	Each session covered specific exercises and topics within the context of mindfulness practice and training. These included various types of formal mindfulness practice, mindful awareness of dynamic yoga postures, and mindfulness during stressful situations and social interactions. The retreat included a combination of	Participants were asked to commit themselves to daily homework assignments of 45–60 minutes.	Pre- and post-intervention 1-hour personal interviews were conducted by each instructor to establish rapport and to help participants	Instructors were two women with university level degrees in educational counseling who had undergone MBSR training provided by the University of Massachusetts	In-person group sessions. Setting not reported.	**Duration of intervention:** 8 weeks **Number of sessions:** 8x standard sessions + 1x silent retreat session **Session frequency:** 1	None reported

		previously used and newly introduced mindfulness exercises.		formulate realistic individual goals for the intervention. Post-intervention interviews addressed participants' personal experiences during the course and assessed the degree to which pre-treatment goals had been met.	Medical Center for Mindfulness. Each had at least seven years of previous experience teaching MBSR, as well as experience teaching people with fibromyalgia.		standard session per week **Session length:** Standard sessions – 2.5 hours; Silent retreat session – 7 hours	
Sephton et al., 2007	MBSR	Participants received MBSR in the format of the parent program (University of Massachusetts). The MBSR program is an outpatient intervention intended to provide participants with a systematic introduction to the practice of mindfulness meditation. Three techniques comprise the foundation of the program, all of which involve the systematic allocation of attention in the context of a relaxed, yet alert state of mind. First, in the body scan technique, attention is directed throughout the body (from toes to head) while participants are lying down in a	Daily home practice of 30 – 45 minutes duration was encouraged on 6 days per week.	Nil	A licensed clinical psychologist who was trained in MBSR at the University of Massachusetts Medical School.	In-person group sessions. Setting not reported.	**Duration of intervention:** 8 weeks **Number of sessions:** 8x standard sessions + 1x silent retreat session **Session frequency:** 1 standard session per week **Session length:** Standard sessions – 2.5 hours; Silent	None reported

		relaxed state with instruction to take note of sensations that may be present. Second, a series of simple yoga positions are taught as a means of encouraging relaxed and focused movement. Third, sitting meditation teaches participants to direct attention systematically, beginning with movement of the breath and proceeding to awareness of other immediate sensory and cognitive experiences. These techniques are augmented with other cognitive exercises and home practice assignments in which program participants learn to be more responsive and less reactive in the face of challenging stressful situations. Home practice assignments were guided by a workbook and audiotapes.					retreat session – Full day	
Shah et al., 2024	Mindfulness + Exercise and Education	The mindfulness components of the intervention were adapted from the MBSR program and an investigator's prior work with mindfulness for chronic low back pain. The curriculum included a review of the biopsychosocial model of pain processing, its relationship to mindfulness, and chair and standing meditations focused on breath, body scan, or counting. The innovative components of the program included integration of mindfulness with exercise.	For home practice, participants were advised to practice strengthening exercises mindfully at least two days each week and any aerobic exercise mindfully at least five days each week. They were also	All study participants participated in an exercise program and received educational materials (see Appendix 3 for further details).	The program was delivered jointly by a physical therapist and a mindfulness instructor with 20 years of experience.	Online live group sessions.	**Duration of intervention:** 8 weeks **Number of sessions:** 8 **Session frequency:** 1 session per week **Session length:** 2 hours	None reported

		Each session was two hours in length divided into four 30-minute blocks. In the first and third 30-minute blocks, participants were trained to develop moment-to-moment awareness using breath, body sensations, or counting as an anchor. In the second and the fourth 30-minute blocks, participants performed the same exercises as the exercise group. In the first two sessions, the mindfulness and exercise components were kept separate. Starting with the third session, participants were instructed to practice mindfulness (i.e., use breath, body sensation, or counting as an anchor) while performing exercise movements.	asked to practice meditations for at least 20-minutes each day.					
Sharpe et al., 2025	Online MBSR	We adapted the MBSR program with people with lived experience of rheumatoid arthritis to address relevant issues for this population. The five lessons cover the following topics: (1) an introduction to mindfulness meditation; (2) dealing with stress; (3) dealing with difficult sensations and emotions; (4) dealing with difficult thoughts; and (5) mindful communication, compassion, and relapse prevention. Each session was accompanied by an audiotape of guided meditation, based on scripts	Participants were encouraged to practice mindfulness meditations throughout the program. Further details not provided.	Nil	Not applicable	Online asynchronous (self-paced) individual sessions.	**Duration of intervention:** 8 weeks **Number of sessions:** 5 **Session frequency:** Approximately 1 session per fortnight **Session length:** 20-30 minutes	None reported

		publicly available from Jon Kabat-Zinn.						
Shokouhi Nejad et al., 2022	MBCT	The treatment protocol was based on Segal's MBCT program. Descriptions of each session are provided: (1) Establishing communication, practicing physical examination, and focusing on daily activities; (2) Exercises related to physical examination, presence of mind on the flow of breathing for 10 minutes, explaining emotion and identifying positive and negative emotions, training to record desirable events, turning attention to daily activities with the presence of mind; (3) Seeing and hearing meditation, meditation in a sitting position (presence of mind related to breathing and body), walking with presence of mind, practicing the three-minute breathing space, recording undesirable events; (4) Seeing and hearing meditation, meditation in a sitting position (awareness of breathing, body, sounds, and thoughts), explanation of the principles governing emotion, regular three-minute breathing space, and three-minute confrontational breathing space; (5) Sitting meditation (awareness of breathing, body parts, sounds and thoughts), how to react to thoughts,	Details not provided	Nil	Not reported	In-person group sessions. Setting not reported.	**Duration of intervention:** 8 weeks **Number of sessions:** 8 **Session frequency:** 1 session per week **Session length:** 1.5 hours	None reported

		feelings and bodily sensations, regular three-minute breathing space, three-minute confrontational breathing space, and introducing the concept of acceptance; (6) Meditation in a sitting position (becoming aware of breathing, body parts, sounds and thoughts), communicating with one's thoughts and feelings, and accepting unpleasant thoughts and emotions; (7) Meditation in a sitting position (awareness of breathing, body parts, sounds and thoughts), awareness of the relationship between activity and mood, making a list of enjoyable activities and activities that create a sense of mastery in the person, regular three-minute breathing space, and three-minute confrontational breathing space; (8) Reflection on body examination, course review.						
Sobhani et al., 2019	MBSR	A description of each session is provided: (1) Set up general policy, consider privacy and personal life, invite participants to introduce themselves, practice physical examination, assign homework, discuss and schedule weekly meetings, and distribute tapes and pamphlets; (2) Conduct relaxation training for 14 muscle groups, including forearms, arms, back muscles,	Home practices were prescribed. Further details were not reported.	Nil	Not reported	In-person group sessions. Setting not reported.	**Duration of intervention:** 8 weeks **Number of sessions:** 8 **Session frequency:** 1 session per week **Session length:** 2 hours	None reported

		thighs, abdomen, chest, shoulders, neck, lips, eyes, and forehead (3) Provide meditation training and review past sessions; (4) Teach conscious breathing techniques, including calm inhalation and exhalation, observing breathing, and assign homework for practice before sleep; (5) Teach the technique of paying attention to body movement while breathing, focusing on body parts and their movements, and paying attention to physical sensations; (6) Train participants to pay attention to the mind, recognizing positive and negative thoughts, allowing them to enter and exit the mind without judgment; (7) Conduct 40 minutes of sitting meditation, review homework, and practice observing the connection between activity and mood; (8) Review past sessions, summarize materials, and hold a question-and-answer session.						
Taub et al., 2024	Modified MBSR	The standard MBSR protocol was adapted in several ways: (1) The intervention was extended into a 10-week program with 11 sessions, which included shortened 2-hour weekly sessions, due to potential difficulties of participants with prolonged sitting; (2) The protocol	Details not reported	Nil	Not reported	In-person group sessions. Setting not reported.	**Duration of intervention:** 10 weeks **Number of sessions:** 11 **Session frequency:** Approximately 1 session per week	None reported

		included shorter, more carefully paced exercises, which gradually progressed from "easy" to "hard". This was intended to facilitate participants' encounters with their body and physical sensations. An emphasis was placed on the concept of "pacing", where participants were encouraged to strategize how much energy to exert according to which activities were planned for the day. "Pacing" would prevent participants from being overly active when energy is available and feeling depleted by the end; (3) Therapists were instructed to pay special attention to fibromyalgia-related themes (e.g., pain and fatigue) throughout the sessions and practices, and to translate the general components of standard MBSR into specific components relevant to fibromyalgia. For example, the issue of automatic thinking in the standard protocol would be translated into specific discourse about automatic pain-related thoughts and pain catastrophizing; (4) Therapists were instructed to specifically target maladaptive avoidance behaviors. Such behaviors are common among people with fibromyalgia.					**Session length:** 2 hours	

Van Gordon et al., 2017	Mindfulness Awareness Training	A second-generation mindfulness-based intervention. Compared to first-generation mindfulness-based interventions, second-generation mindfulness-based interventions are more acknowledging of the spiritual aspect of mindfulness. The weekly sessions comprise three distinct phases: (1) a taught/presentation component (approximately 45 minutes); (2) a facilitated group discussion component (approximately 30 minutes); and (3) guided mindfulness meditation and/or mindfulness exercises (approximately 30 minutes). A 5-minute break is scheduled after the presentation components, and a 10-minute break is scheduled prior to commencing the mindfulness practices. In order to directly target the key symptoms and correlates of fibromyalgia syndrome, the intervention was also modified in this study to include an extended focus on: (1) mindfulness techniques specifically concerned with meditatively observing and objectifying somatic pain; (2) compassion meditation in order to help participants become less preoccupied with their illness (i.e., by becoming more aware of the suffering of	Rather than prescribe a fixed amount of daily meditation practice time, participants were encouraged to adopt a dynamic meditation routine and were guided on an individual basis to find the optimum frequency and duration of meditation sessions.	Mindfulness group participants attended two 50-minute one-to-one support sessions with the instructor, in addition to the group mindfulness sessions.	Instructors who had undergone a 3-year supervised Mindfulness Awareness Training training program.	In-person group sessions delivered across multiple sites in the East Midlands in separate training rooms utilized by a meditation center and general practice surgery. Other than an overhead projector, chairs and tables, a singing bowl for use during the guided meditations, and sufficient space to practice walking meditation, no special equipment or arrangements were required.	**Duration of intervention:** 8 weeks<br>**Number of sessions:** 8<br>**Session frequency:** 1 session per week<br>**Session length:** 2 hours	In the third and eighth week of the program, mindfulness group participants attended one-to-one support sessions (each of 50-minutes duration) with the instructor. Participants were guided on an individual basis to find the optimum frequency and duration of home meditation practice sessions.

		others); and (3) 'engaged mindfulness' (a technique intended to raise participants' awareness of the benefits – to both themselves and others – of contributing to the welfare of society in a manner that does not exceed the physical and/or psychological demands of their condition). Participants were provided with a CD of guided meditations to facilitate daily self-practice.						
Zangi et al., 2012	Vitality Training Program + Usual Care	Through mindfulness-based exercises, participants were encouraged to become aware of, and intentionally attend to, their emotions, thoughts and bodily experiences. In addition, various creative exercises, such as guided imagery, music, drawing, poetry and metaphors, were used to encourage experiential learning processes. Reflections on individuals' own experiences were promoted through writing, sharing, and listening to one another within the group.	Between sessions the participants performed awareness and relaxation training exercises and wrote reflective diaries.	All study participants received routine care throughout the study including the provision of a CD with mindfulness-based home exercises for individual voluntary use.	The group facilitators were health professionals (physiotherapists, occupational therapists, nurses and social workers) who had been trained in mindfulness-based exercises and delivery of the Vitality Training Program. Their training comprised six 3-day workshops over one year, together with home assignments and a practical examination at the end of the training.	In-person group sessions. Setting not reported.	**Duration of intervention:** Main intervention – 15 weeks; 1 booster session provided at 6 months **Number of sessions:** Main intervention – 10; 1 booster session **Session frequency:** Main intervention – 1-2 sessions per fortnight **Session length:** 4.5 hours	None reported

Zautra et al., 2008	Mindfulness-Based Emotion Regulation Therapeutic Program	Within each session, therapists introduced didactic information, implemented skill-related exercises, reviewed with individual participants their understanding and learning of the specific skills, and assigned weekly homework related to session activities. Therapists then reviewed homework at the beginning of the next session to reinforce adherence and to problem solve application difficulties. The mindfulness intervention was designed to develop two distinct sets of skills, one to reduce the negative impact of stressful life events and illness burdens, and the other to enhance the ability to sustain positive social engagements despite pain and stress. The treatment modules included: (1) mindfulness and the bidimensional model of emotion; (2) mindfulness and awareness; (3) emotional clarity and well-being; (4) acceptance, negative thoughts, and reframing; (5) positive emotions and pleasant event scheduling; (6) enhancing social relations; (7) intimacy, stress, and mindfulness; and (8) maintenance and generalization. The modules were organized so that early sessions introduced the	Participants were encouraged to perform a 10-minute sitting meditation as a home practice.	Nil	A doctoral-level psychologist and an advanced doctoral student in clinical psychology.	In-person group sessions. Setting not reported.	**Duration of intervention:** 8 weeks **Number of sessions:** 8 **Session frequency:** 1 session per week **Session length:** 2 hours	None reported

		concept of two distinct dimensions of emotional health, positive and negative, and the role of mindfulness meditation practice in promoting awareness and acceptance of the full range of emotional experiences. Subsequent sessions included didactic activities and experiential exercises to more fully develop understanding of emotional awareness and acceptance and to foster the development of positive emotional and social resources. The intervention drew on mindfulness meditation aspects of the MBSR approach developed by Kabat-Zinn and Segal et al's MBCT.						
Zgierska et al., 2016	Mindfulness Meditation Intervention	Patterned after existing mindfulness-based programs and adapted to the psychophysical needs of the study population, the intervention combined mindfulness meditation and pain-specific CBT strategies. Each session included a review of home practice and questions, introduction to the session's core concepts, and session-specific exercises, followed by discussion of participant experiences and skills for coping with challenges related to chronic low back pain. Session-specific exercises	Participants were encouraged to practice formal mindfulness meditation (e.g., breath meditation or body scan meditation) at least 6 days per week for at least 30 minutes per day, and to engage in daily, brief mindfulness	Usual care for chronic low back pain and opioid therapy management was provided to all participants by their regular clinicians (see Appendix 3 for further details).	Two psychologists experienced with working with people with pain, both with over 20 years of personal meditation practice and over 10 years of meditation teaching experience.	In-person group sessions. Setting not reported.	**Duration of intervention:** 8 weeks **Number of sessions:** 8 **Session frequency:** 1 session per week **Session length:** 2 hours	None reported

		included extensive engagement in mindfulness techniques to facilitate learning of mindfulness-based pain coping. Key mindfulness techniques included (1) breath meditation practice of bringing awareness to the breath after the mind wanders away, providing practice of not becoming engaged in catastrophizing or rumination about pain; (2) body scan meditation practice of awareness of interoceptive, bodily sensations while engaged in acceptance and nonjudgment of present-moment experiences, including pain; (3) mindful walking, movement or stretching to further practice mindful awareness of interoceptive sensations, such as pain, during movement and facilitate better understanding of one's physical capacity, which, in turn, may promote a healthy engagement in physical activity; (4) loving kindness meditation to practice kindness to and acceptance of, rather than judgment of, one's pain experience; and (5) brief mindfulness techniques designed to be used throughout the day for an "informal" practice or for as-needed pain or stress coping. These "mini-meditations"	practices through the duration of the study.						

		included pain-wave surfing and SABER (stop, acknowledge, breathe, expand, respond) techniques. Their goal was to enable practice of acceptance and nonjudgmental awareness of sensations, thoughts, and emotions within present-moment experiences to allow opportunity for a mindful, healthy response, rather than a habitual, maladaptive reaction (e.g., catastrophizing, rumination, acting out), to pain and other daily stressors.						
Zgierska et al., 2025	Mindfulness-Based Therapy + Usual Care	Patterned after established MBSR curricula, and the programs that grew from it including MBCT, MBRP, and MORE. Involved training in mindfulness skills and applied mindfulness to deconstruct pain into constituent cognitive, emotional, and sensorial components and adaptively cope with pain and negative emotions. Included formal, longer mindfulness practices and brief techniques for practicing mindfulness skills throughout the day. Weekly topics included: Automatic Pilot and Pain, Awareness of Automatic Reactions, Are Thoughts Facts, Staying Present and Aware During Challenging Pain Situations, Balancing Acceptance and Skillful Mindful Action, Mindful Balance in Daily Life, Mindful	Participants were encouraged to practice formal mindfulness meditation on at least 6 days per week for at least 30 minutes per day.	All participants were instructed to continue with "usual care" as recommended and provided by their regular pain care clinicians. Usual treatment could include physical therapy, acupuncture, chiropractic care, hydrotherapy, medication therapy, vitamins and herbal remedies, procedural	Trained, licensed therapists experienced in Mindfulness-Based Therapy and trained in the intervention protocol.	Group sessions, initially delivered in-person, then remotely due to COVID-19 restrictions.	**Duration of intervention:** 8 weeks **Number of sessions:** 8 **Session frequency:** 1 session per week **Session length:** 2 hours	Intervention sessions were planned to be delivered in person but were transitioned to online delivery during the course of the study due to COVID-19 restrictions.

		Communication and Loving Kindness, and Balanced Living: Building Support Networks and Continuing to Live Mindfully. The intervention sessions started with a review of home practice, experiences, concerns and questions, followed by an introduction to the session-specific core concepts and 2 to 3 mindfulness-based therapy activities, each followed by discussion of participant experiences and skills for coping with challenges related to opioid-treated chronic low back pain. Each session ended with an overview of the home practice for the following week or for the remaining follow-up period.		therapies, and other interventions.				
Zou et al., 2024	MBSR + Exercise Program + Usual Care	Key components of the MBSR program included: (1) Explaining relevant knowledge, methods and effects of MBSR; (2) Body scan meditation – guiding the participant to lie flat, relax the body and close the eyes, listen to the audio and feel the changes in various parts of the body; (3) Mindful breathing – guiding the participant to focus on breathing, imagine that the physical discomfort slowly disappears with each breath; (4) Mindfulness meditation – guiding the participant to release negative emotions in meditation; (5) Walking	Details not reported	All study participants received usual care (see Appendix 3 for further details).<br><br>Intervention group participants were also encouraged to participated in an individual, unsupervised exercise three times per week.	Trained nurses.	In-person group sessions. Setting not reported.	**Duration of intervention:** 4 weeks<br>**Number of sessions:** 4<br>**Session frequency:** 1 session per week<br>**Session length:** 1 hour	None reported

		meditation – guiding the participant to walk slowly and carefully feeling the soles of the feet touching the ground, telling them to focus on the swing of the arms and train their perception of the body; (6) Mindful yoga – the participant follows the guidance of the instructor to perform yoga combined with abdominal breathing, and feel the physical state during the practice; (7) Mindful Introspection – guiding the participant to enter the world of imagination and deeply ponder their inner thoughts; and (8) Guiding participants to face the future, believe that they can overcome their condition, feel life calmly, and practice mindfulness meditation every day.		Participants were instructed to perform a warm-up before exercising, including relaxing the muscles through massage. Prescribed exercises included: standing trunk rotation, standing trunk flexion and extension, standing and sitting neck rotation, standing and sitting neck flexion and extension, standing chest stretch, and prone extension with the arms and legs lifted.				

CBT = Cognitive behavioral therapy; MBCT = Mindfulness-Based Cognitive Therapy; MBRP = Mindfulness-Based Relapse Prevention; MBSR = Mindfulness-Based Stress Reduction; MORE = Mindfulness-Oriented Recovery Enhancement.
