# Supplementary material for: The Effects of Mindfulness‐Based Programs on Pain‐Related Cognitions in Individuals With Chronic Musculoskeletal Pain: A Systematic Review and Meta‐Analysis
Source: Pain Res Manag. 2026 Nov 9;2026:6495015. doi: 10.1155/prm/6495015 (PMC13746321; doi:10.1155/prm/6495015)
Supplement: Supplementary file 1 [file PRM-2026-6495015-s001.zip › Supplemental Digital Content 5_GRADE Profile_MIND SR_v5_01072026.pdf]

Supplemental Digital Content 5: GRADE Evidence Profile

	**Certainty Assessment**				
**Outcomes**	**Risk of Bias[a]**	**Inconsistency of Effect[b]**	**Imprecision[c]**	**Publication Bias[d]**	**Overall Certainty of Evidence**
**Pain Catastrophizing** Mindfulness vs No Intervention	↓	↓		↓	Very Low
**Pain Catastrophizing** Mindfulness vs Education	↓	↓			Low
**Chronic Pain Acceptance** Mindfulness vs No Intervention	↓	↓		↓	Very Low
**Depression** Mindfulness vs No Intervention	↓	↓		↓	Very Low
**Anxiety** Mindfulness vs No Intervention	↓	↓		↓	Very Low
**Psychological Distress** Mindfulness vs No Intervention	↓	↓	↓		Very Low
**Pain Intensity** Mindfulness vs No Intervention	↓	↓			Low
**Pain Intensity** Mindfulness vs Cognitive Behavioral Therapy	↓	↓			Low
**Pain Intensity** Mindfulness vs Education	↓	↓			Low
**Disability** Mindfulness vs No Intervention	↓	↓		↓	Very Low

[a] >25% of participants included in the meta-analysis were from studies with a high risk of bias

[b] Large heterogeneity between studies, defined as I^2 >50%

[c] Pooled sample size of <400 participants

[d] Assessed through visual inspection of funnel plots and the use of Egger's test with p<0.1 considered evidence of publication bias OR suspected if the body of evidence consisted of only small studies showing preferred results and if eligible studies were identified in trial registries but were not published

↓ Downgraded
