# Supplementary material for: The Effects of Mindfulness‐Based Programs on Pain‐Related Cognitions in Individuals With Chronic Musculoskeletal Pain: A Systematic Review and Meta‐Analysis
Source: Pain Res Manag. 2026 Nov 9;2026:6495015. doi: 10.1155/prm/6495015 (PMC13746321; doi:10.1155/prm/6495015)
Supplement: Supplementary file 1 [file PRM-2026-6495015-s001.zip › Supplemental Digital Content 6_v5_13032026.pdf]

Supplemental Digital Content 6: Supplementary Findings

Outcome	Comparison	Study	Main Findings	Overall ROB
Pain Catastrophizing	MBSR + Usual Care *vs* Usual Care Alone	Cherkin et al., 2016	At 12-months post-randomization, pain catastrophizing (13-item PCS) decreased more in the MBSR group than in the control group (MBSR vs control adjusted mean [95% CI] difference in change = -2.82 [-4.68 to -0.96]).	High
		Pérez-Aranda et al., 2019	MBSR was superior to control for reductions in pain catastrophizing (13-item PCS) at 12-months post-randomization (regression coefficient = -7.06, [95% CI -11.41 to -2.07], effect size [Cohen's d] = 0.73).	High
	Mindfulness-Based Program *vs* CBT	Cherkin et al., 2016	Pain catastrophizing (13-item PCS) decreased more from pre- to post-intervention in the MBSR group than in the CBT group (MBSR vs CBT adjusted mean [95% CI] difference in change = -1.81 [-3.30 to -0.01]. There was no significant difference between groups at 12-months (MBSR vs CBT adjusted mean [95% CI] difference in change = 0.76 [-1.07 to 2.59]) .	Some concerns
		Zautra et al., 2008	Among participants with a history of recurrent depression, the mindfulness group showed lower pain catastrophizing scores post-intervention than the CBT group.	High
	MBSR + Usual Care *vs* FibroQoL + Usual Care	Pérez-Aranda et al., 2019	Compared with FibroQoL + Usual Care, MBSR + Usual Care resulted in significant moderate-to-large improvements in pain catastrophizing (13-item PCS) immediately post-intervention (regression coefficient = -4.35 [95% CI -8.13 to -0.56]; effect size [Cohens' d] = 0.65) and at 12 months post-randomization (regression coefficient = -4.35 [95% CI -8.13 to -0.56]; effect size [Cohens' d] = 0.58).	Some concerns
Chronic Pain Acceptance	MBSR + Usual Care *vs* Usual Care Alone	Cherkin et al., 2016	There was no significant difference in chronic pain acceptance (CPAQ-8) between groups at 12-months (MBSR vs control adjusted mean [95% CI] difference in change = 0.71 [-0.87 to 2.29].	High
	Modified MBSR *vs* Pain Education	Pal et al., 2023	Variance comparison between the mindfulness group and pain education group showed a statistically significant difference in chronic pain acceptance (CPAQ-8) ($F[1,326] = 8.8186$; $p = 0.003$), favoring the mindfulness group.	High

		Pal et al., 2024	Mindfulness-based program participants experienced greater improvements in chronic pain acceptance than participants in the education group at post-intervention (F = 56.68; p < 0.001).	High
	MBSR + Usual Care *vs* CBT + Usual Care	Cherkin et al., 2016	There was no significant difference in chronic pain acceptance (CPAQ-8) between groups from pre- to post-intervention (MBSR vs CBT adjusted mean [95% CI] difference in change = 0.94 [-0.48 to 2.36]) or at 12-months (MBSR vs CBT adjusted mean [95% CI] difference in change = -0.82 [-2.45 to 0.82]).	Some concerns
Pain Self-Efficacy	Mindfulness-Based Program *vs* No Intervention	Cherkin et al., 2016	Pain self-efficacy (PSEQ) increased significantly more from pre- to post-intervention in the MBSR group than in the control group (MBSR vs control adjusted mean [95% CI] difference in change = 3.03 [1.23 to 4.82]. There was no significant difference between groups at 12-months (MBSR vs control adjusted mean [95% CI] difference in change = 0.43 (-1.36 to 2.23]).	High
		Shah et al., 2024	There was no significant difference in pain self-efficacy (CPSES self-efficacy for pain management subscale) between groups at post-intervention (between group difference, baseline to post-intervention, mean (95% CI) = -2.1 [-12.3 to 8.1]).	Some concerns
		Zangi et al., 2012	Significant effects on pain self-efficacy (ASES pain subscale) in favour of the mindfulness-based program were found at post-intervention (mean difference between groups = 8.2, [95% CI 2.1 to 14.2]; effect size [Cohens' d] = 0.54), and at 12-months (mean difference between groups = 9.1, [95% CI 3.4 to 14.8]; effect size [Cohens' d] = 0.59).	High
	Mindfulness-Based Program *vs* Education	Davis et al., 2013	The mindfulness group experienced increasing levels of pain self-efficacy (0-5 Likert Scale) from pre- to post-intervention (t = 4.52, p < 0.0001), whereas the education group showed no improvement over time (t = 0.54, p = ns) .	High
		Morone et al., 2009	Both the mindfulness-based program group and CBT group showed improvements in pain self-efficacy immediately post-intervention, with no significant	Some concerns

			difference between the two groups.	
		Morone et al., 2016	Participants in the mindfulness group experienced greater increases in pain self-efficacy (CPSES self-efficacy for pain management subscale) at post-intervention than participants in the education group (adjusted mean between-group difference = 9.8, [95% CI 5.3 to 14.3]).	Some concerns
		Zautra et al., 2008	Participants in the mindfulness group experienced greater increases in pain self-efficacy at post-intervention than participants in the education group.	High
	Mindfulness-Based Program *vs* CBT	Cherkin et al., 2016	Pain self-efficacy (PSEQ) increased from pre- to post-intervention with MBSR and CBT, with no difference between the groups (MBSR vs CBT adjusted mean [95% CI] difference in change = 0.34 [-1.43 to 2.10]). Improvements were not maintained in either group at 12-months.	Some concerns
		Zautra et al., 2008	Both the mindfulness-based program group and CBT group showed improvements in pain self-efficacy immediately post-intervention, with no significant difference between the two groups. However, participants in the mindfulness group with a history of recurrent depression showed lower pain self-efficacy scores post-intervention than participants with recurrent depression in the CBT group.	High
Kinesiophobia	Mindfulness-Based Program *vs* No Intervention	Bagheri et al., 2021	Kinesiophobia (TSK) decreased from pre- to post-intervention for participants in the mindfulness group, but not for participants in the control group (mean between group difference = -7.9, [95% CI -11.6 to -4.1]).	Some concerns
		Robles et al., 2025	There were no significant effects of group ($F[1, 24] = 0.75$; $p = 0.39$) or group x time interaction ($F[1, 24] = 0.81$; $p = 0.37$).	High
		Safavi-Farokhi et al., 2020	Participants in the mindfulness group demonstrated greater reductions in kinesiophobia (TSK) at post-intervention than participants in the control group ($p < 0.05$).	High
Pain Control	Mindfulness-Based Pain Management *vs* No Intervention	Brown & Jones, 2013	Participants in the mindfulness-based program group experienced greater perceived control over pain at post-intervention compared with	High

			participants allocated to no intervention (p = 0.01).	
	Mindfulness-Based Program *vs* Education	Zautra et al., 2008	Participants in the education group reported greater improvements in pain control from pre- to post-intervention than participants in the mindfulness group (education pre M = 5.98 [SE 0.32], post M = 6.24 [SE 0.33]; mindfulness pre M = 7.11 [SE 0.03], post M = 7.04 [SE 0.32]).	High
	Mindfulness-Based Program *vs* CBT	Zautra et al., 2008	Participants in the CBT group reported greater improvements in pain control from pre- to post-intervention than participants in the mindfulness group (CBT pre M = 5.83 [SE 0.30], post M = 6.13 [SE 0.30]; mindfulness pre M = 7.11 [SE 0.03], post M = 7.04 [SE 0.32]).	High
Depression	Mindfulness-Based Program *vs* No Intervention	Burns et al., 2022	Participants allocated to no intervention did not show significant changes in depression (CES-D Short Form) at post-intervention, whereas participants allocated to the mindfulness-based program improved significantly over the course of treatment (Effect size of interaction [Cohen's d] [95% CI] = 0.26 [0.08 to 0.42]).	High
		Cherkin et al., 2016	There was no significant difference in depression (PHQ-8) between groups at 12-months post-randomization (MBSR vs control mean [95% CI] difference in change = -0.62 [-1.48 to 0.23]).	High
	Mindfulness-Based Program *vs* CBT	Cherkin et al., 2016	Depression (PHQ-8) decreased more from pre- to post-intervention in the CBT group than in the MBSR group (CBT vs MBSR mean [95% CI] difference in change = -0.69 [-1.35 to -0.02]). There was no significant difference between groups at 12-months (CBT vs MBSR mean [95% CI] difference in change = -0.21 [-1.03 to 0.61]).	Some concerns
		Sharpe et al., 2025	MBSR and CBT were equally efficacious in reducing depression (PHQ-9) (MSBR: Pre EMM = 8.07 [SE 0.23], Post EMM = 0.5.83 [SE 0.36]; CBT: Pre EMM = 0.8.33 [SE 0.22], Post EMM = 5.66 [SE 0.35]).	High
		Zautra et al., 2008	Depression symptoms decreased as a function of time among mindfulness and CBT participants, but no group differences emerged.	High
	Mindfulness-Based Program *vs* Education	Pal et al., 2024	Mindfulness-based program participants experienced greater improvements in depression than participants in the education group	High

			at post-intervention (F = 39.21; p < 0.001).	
		Zautra et al., 2008	Depression symptoms decreased as a function of time among mindfulness and education participants, but no group differences emerged.	High
	Mind-Body Intervention (Mindfulness Meditation + Qigong) *vs* Health Education	Astin et al., 2003	Both groups registered statistically significant improvements at post-intervention for depression (BDI) (F[3, 186] = 17.0; p < 0.001). There was no significant difference between the groups.	High
	Modified MBCT *vs* Usual Care	Dalili & Bayazi, 2019	MBCT had a statistically significant effect on depression (DASS-21 depression subscale) compared to usual care post-intervention (F = 60.56; p < 0.001).	High
	Mindfulness-Based Program *vs* Cognitive Therapy	Burns et al., 2022	Both groups registered improvements at post-intervention for depression (CES-D Short Form). There was no significant difference between the groups.	Some concerns
		Day et al., 2024	Both groups registered small-to-medium improvements at post-intervention for depression (PROMIS depression domain). There was no significant difference between the groups (between group effect size [Cohens' d] = 0.09 [95% CI -0.21 to 0.38]).	Some concerns
	Mindfulness-Based Program *vs* Behavioral Activation/Behavior Therapy	Burns et al., 2022	Both groups registered improvements at post-intervention for depression (CES-D Short Form). There was no significant difference between the groups.	Some concerns
		Day et al., 2024	Both groups registered small-to-medium improvements at post-intervention for depression (PROMIS depression domain). There was no significant difference between the groups (between group effect size [Cohens' d] = 0.01 [95% CI = -0.29 to 0.31]).	Some concerns
	MBSR *vs* Intensive Short-Term Dynamic Psychotherapy	Farzadkia et al., 2023	Both groups registered improvements at post-intervention for depression (BDI). Intensive Short-Term Dynamic Psychotherapy was slightly more effective than MBSR (mean between group difference = -3.66 [SE 0.61]; p = 0.001).	High
	MBSR *vs* Relaxation	Schmidt et al., 2011	There was no significant difference between the groups (p = 0.19).	High
Anxiety	MBSR + Usual Care *vs* Usual Care Alone	Cherkin et al., 2016	There was no significant difference in anxiety (GAD-2) between groups at 12-months (MBSR vs control mean [95% CI] difference in change = 0.00 [-0.37 to 0.36]).	High

	Mindfulness-Based Program *vs* CBT	Cherkin et al., 2016	There was no significant difference in anxiety (GAD-2) between groups from pre- to post-intervention (CBT vs MBSR mean [95% CI] difference in change = -0.18 [-0.47 to 0.11]) or at 12-months (CBT vs MBSR mean [95% CI] difference in change = -0.24 [-0.56 to 0.08]).	Some concerns
		Sharpe et al., 2025	The treatment × time interaction was not significant for anxiety ($p = 0.14$).	High
	Mindfulness-Based Program *vs* Education	Pal et al., 2024	Mindfulness-based program participants experienced greater improvements in anxiety than participants in the education group at post-intervention ($F = 33.75$; $p < 0.001$).	High
	MBCT *vs* Sham tDCS	Akbarzadeh et al., 2022	MBCT was more effective at reducing anxiety (PSWQ) than sham tDCS ($p < 0.05$).	Some concerns
	Modified MBCT *vs* Usual Care	Dalili & Bayazi, 2019	MBCT had a statistically significant effect on anxiety (DASS-21 anxiety subscale) compared to usual care post-intervention ($F = 48.85$; $p < 0.001$).	High
	Mindfulness Meditation *vs* Cognitive Therapy	Day et al., 2024	Both groups registered small-to-medium improvements at post-intervention for anxiety (PROMIS anxiety domain). There was no significant difference between the groups (between group effect size [Cohens' d] = 0.01 [95% CI -0.28 to 0.31]).	Some concerns
	Mindfulness Meditation *vs* Behavioral Activation	Day et al., 2024	Both groups registered small-to-medium improvements at post-intervention for anxiety (PROMIS anxiety domain). There was no significant difference between the groups (between group effect size [Cohens' d] = 0.09 [95% CI -0.21 to 0.38]).	Some concerns
	MBSR *vs* Usual Care	Donnino et al., 2021	Anxiety (PASS) scores decreased more from pre- to post-intervention in the MBSR group than the usual care group (within group mean difference [95% CI]: MBSR = -13.5 [-23.1, -3.8]; usual care = -4.8 [-15.1, 5.4]).	High
	MBSR *vs* Psychophysiologic Symptom Relief Therapy	Donnino et al., 2021	Mean anxiety (PASS) scores for Psychophysiologic Symptom Relief Therapy lower than those for MBSR at post-intervention (effect size [Cohens' d] = -0.90; $p = 0.01$)	High
	MBSR *vs* Relaxation	Schmidt et al., 2011	There was no significant difference between the groups ($p = 0.19$).	High
Psychological Distress	Mindfulness-Based Program *vs* No Intervention	Pérez-Aranda et al., 2019	MBSR was superior to control for reductions in psychological distress (HADS) at 12-months post-randomization (regression	High

			coefficient = -4.12, [95% CI -7.24 to -1.00], effect size [Cohen's d] = 0.67).	
		Zangi et al., 2012	Significant effects on psychological distress (General Health Questionnaire, 20-item version) in favour of the mindfulness-based program were found at 12-months (mean difference between groups = -3.7, [95% CI -6.3 to -1.1]; effect size [Cohens' d] = 0.58).	High
	MBSR + Usual Care *vs* FibroQoL + Usual Care	Pérez-Aranda et al., 2019	Compared with FibroQoL + Usual Care, MBSR + Usual Care resulted in significant moderate-to-large improvements in psychological distress (HADS) immediately post-intervention (regression coefficient = -2.96 [95% CI -5.57 to -0.34]; effect size [Cohens' d] = 0.49). There was no significant difference between the groups at 12-months post-randomization (regression coefficient = -0.51 [95% CI -3.61 to 2.59]; effect size [Cohens' d] = 0.10).	Some concerns
	MBCT *vs* Compassion Therapy	Shokouhi Nejad et al., 2022	Both groups experienced improvements in psychological distress (DASS-21) at post-intervention. There was no significant difference between the groups (between group mean difference = 2.01 [SE 2.57]; p = 0.441).	High
	Mindfulness Awareness Training *vs* Cognitive Behavioral Therapy for Groups	Van Gordon et al., 2017	Mindfulness Awareness Training participants demonstrated significant improvements in psychological distress (DASS-21) post-intervention over Cognitive Behavioral Therapy for Groups participants (mean difference between groups = -3.70 [95% CI -4.77 to -2.63]; p < 0.001).	High
Pain Intensity	Mindfulness-Based Program *vs* No Intervention	Banth & Ardebil, 2015	Mindfulness-based program participants experienced greater improvements in pain than participants in the control group at post-intervention (F[1, 45] = 110.4; p < 0.001).	High
		Burns et al., 2022	Participants allocated to no intervention did not show significant changes in pain intensity (NRS) at post-intervention, whereas participants allocated to the mindfulness-based program improved significantly over the course of treatment (Effect size of interaction [Cohen's d] [95% CI] = 0.30 [0.15 to 0.49]).	High

		Cherkin et al., 2016	Pain intensity (VAS) decreased more from baseline to 12-months in the MBSR group than in the control group (MBSR vs control mean [95% CI] difference in change = -0.63 [-1.06 to -0.19]).	High
		Mohammadi et al., 2011	MBCT participants experienced greater improvements in pain (Graded Classification of Chronic Pain perceived pain subscale) than participants in the control group at post-intervention (F = 12.34; p = 0.002).	High
		Plews-Ogan et al., 2005	There were no significant differences in pain (NRS) from pre- to post-intervention in either group.	High
		Reiner et al., 2019	There was a significant time x group interaction for self-reported average pain severity measured by BPI (F[1, 32] = 4.49; p = 0.04). The mindfulness group showed a decrease in pain while the control group showed an increase.	High
		Zangi et al., 2012	There was no significant difference in pain intensity (NRS) between groups at 12-months (mean difference between groups = -0.6, [95% CI -1.28 to 0.02]; effect size [Cohens' d] = 0.32).	High
	Mindfulness-Based Program *vs* CBT	Cherkin et al., 2016	Both groups experienced decreases in pain intensity (VAS) from baseline to 12-months, with no significant difference between groups (CBT vs MBSR mean [95% CI] difference in change = -0.02 [-0.44 to 0.48]).	High
		Zgierska et al., 2025	Both groups experienced improvements in pain severity (BPI – pain severity subscale) from baseline to 12-months, with no significant difference between groups (CBT vs Mindfulness-Based Program mean [95% CI] difference in change 0.13 [-0.13 to 0.40]).	Some concerns
	Mindfulness-Based Program *vs* Education	Davis et al., 2013	Neither group showed significant improvement in pain intensity (VAS) at post-intervention (time F = 2.29, p > 0.05; time x group F = 0.55, p > 0.05).	High
		Morone et al., 2009	The differences for pain intensity for the mindfulness-based program and education groups did not reach statistical significance.	Some concerns
	MBCT *vs* Sham tDCS	Akbarzadeh et al., 2022	MBCT was more effective at reducing pain (SF-MPQ) than sham tDCS (p < 0.05).	Some concerns
	Mind-Body Intervention	Astin et al., 2003	Both groups registered statistically significant improvements at post-	High

	(Mindfulness Meditation + Qigong) *vs* Health Education		intervention for pain (SF-36 pain subscale) (F[3, 189] = 10.5; p < 0.001). There was no significant difference between the groups.	
	Mindfulness + Neuromuscular Exercises vs Neuromuscular Exercises + Motivational Interviewing	Chaharmahali et al., 2023	Both groups experienced improvements in pain (VAS) at post-intervention (p < 0.05). Neuromuscular Exercises + Motivational Interviewing had a more significant effect on pain than Mindfulness + Neuromuscular Exercises.	Some concerns
	Mindfulness-Based Program *vs* Cognitive Therapy	Burns et al., 2022	Both groups registered improvements at post-intervention for pain intensity (NRS). There was no significant difference between the groups.	Some concerns
		Day et al., 2019	Improvement in pain intensity (NRS) across all three groups (Mindfulness Meditation, MBCT, and Cognitive Therapy) combined at post-intervention was large and significant (F[1, 65] = 14.30; p < 0.001), but the between-group effect size was small, with no significant between-group differences in improvement F[2, 65] = 2.14; p = 0.126).	Some concerns
		Day et al., 2024	Both groups registered small-to-medium improvements at post-intervention for pain (NRS). There was no significant difference between the groups (between group effect size [Cohens' d] = - 0.10 [95% CI -0.39 to 0.20]).	Some concerns
	Mindfulness-Based Program *vs* Behavioral Activation/Behavior Therapy	Burns et al., 2022	Both groups registered improvements at post-intervention for pain intensity (NRS). There was no significant difference between the groups.	Some concerns
		Day et al., 2024	Both groups registered small-to-medium improvements at post-intervention for pain (NRS). There was no significant difference between the groups (between group effect size [Cohens' d] = -0.05 [95% CI -0.35 to 0.24]).	Some concerns
	MBSR *vs* Massage	Plews-Ogan et al., 2005	Massage was more effective at reducing pain post-intervention than MBSR.	High
	MBCT *vs* Compassion Therapy	Shokouhi Nejad et al., 2022	Both groups experienced improvements in pain intensity (Chronic Pain Grade Questionnaire) at post-intervention. There was no significant difference between the groups (between group mean difference = -2.03 [SE 3.85]; p = 0.60).	High

Disability	Mindfulness-Based Program *vs* No Intervention	Burns et al., 2022	Participants allocated to no intervention did not show significant changes in disability (SF-36 physical function subscale) at post-intervention, whereas participants allocated to the mindfulness-based program improved significantly over the course of treatment (Effect size of interaction [Cohen's d] [95% CI] = -0.21 [-0.34 to -0.04]).	High
		Cherkin et al., 2016	Participants randomized to MBSR were more likely than participants randomized to control to show clinically meaningful improvement in disability (RDQ) at 12-months (RR 1.41, [95% CI 1.13 to 1.77]). The difference was not maintained at two years (MBSR vs control mean [95% CI] between group difference in RDQ = -1.35 [-2.80 to 0.11].	High
		Mohammadi et al., 2011	MBCT participants experienced greater improvements in disability (Pain Disability Questionnaire) than participants in the control group at post-intervention (F = 8.04; p = 0.01).	High
		Safavi-Farokhi et al., 2020	Participants in the mindfulness group demonstrated greater reductions in disability (ODI) at post-intervention than participants in the control group (p < 0.05).	High
		Zgierska et al., 2016	No statistically significant between-group differences in disability (ODI) were noted over time (p > 0.05).	High
	Mindfulness-Based Program *vs* Education	Pal et al., 2024	Mindfulness-based program participants experienced slightly greater improvements in disability (ODI) than participants in the education group at post-intervention (F = 4.50; p < 0.03).	High
		Morone et al., 2009	Both groups experienced improvements in disability (RDQ) at post-intervention. There was no significant difference between the groups.	Some concerns
		Morone et al., 2016	Participants in the mindfulness group experienced greater improvements in disability (RDQ) at post-intervention than participants in the education group (adjusted mean between-group difference = -1.1, [95% CI -2.1 to -0.01]).	Some concerns
	Mindfulness-Based Program *vs* CBT	Cherkin et al., 2016	While both groups showed improvements in disability (RDQ) at all timepoints, there were no significant differences between groups at post-intervention (CBT vs	Some concerns

			MBSR mean [95% CI] difference in change from baseline = 0.04 [-1.11 to 1.18]), at 12-months (CBT vs MBSR mean [95% CI] difference in change from baseline = 0.51 [-0.75 to 1.78]), or at two years (CBT vs MBSR mean [95% CI] difference in change = -0.50 [-1.93 to 0.94]).	
		Sharpe et al., 2025	There was no significant difference in disability (HAQ-DI) between the groups at post-intervention (MSBR: Pre EMM = 0.47 [SE 0.02], Post EMM = 0.48 [SE 0.03]; CBT: Pre EMM = 0.49 [SE 0.02], Post EMM = 0.47 [SE 0.03]).	High
		Zgierska et al., 2025	While both groups showed improvements in disability (ODI) at all timepoints, there were no significant differences between groups at post-intervention (CBT vs Mindfulness-Based Program mean [95% CI] difference in change from baseline = 0.31 [-1.50 to 2.13]), or at 12 months (CBT vs Mindfulness-Based Program mean [95% CI] difference in change from baseline = 0.27 [-1.59 to 2.12]).	Some concerns
	Mindfulness + Neuromuscular Exercises *vs* Neuromuscular Exercises + Motivational Interviewing	Chaharmahali et al., 2023	Both groups experienced significant improvements in disability (WOMAC) at post-intervention ($p < 0.05$). Neuromuscular Exercises + Motivational Interviewing had a more significant effect on disability than Mindfulness + Neuromuscular Exercises ($p = 0.004$).	Some concerns
	Mindfulness Meditation *vs* Cognitive Therapy	Burns et al., 2022	Both groups registered improvements at post-intervention for disability (SF-36 physical function subscale). There was no significant difference between the groups.	Some concerns
		Day et al., 2019	Large improvements in post-intervention scores for disability (PROMIS physical function domain) were found ($p < 0.001$), with no significant differences between the three groups (Mindfulness Meditation, MBCT, and Cognitive Therapy).	Some concerns
		Day et al., 2024	Both groups registered small-to-medium improvements at post-intervention for disability (PROMIS physical function domain). There was no significant difference between the groups (between group effect size [Cohens' d] = -0.14 [95% CI -0.43 to 0.16]).	Some concerns
	Mindfulness-Based Program *vs* Behavioral	Burns et al., 2022	Both groups registered improvements at post-intervention	Some concerns

	Activation/Behavior Therapy		for disability (SF-36 physical function subscale). There was no significant difference between the groups.	
		Day et al., 2024	Both groups registered small-to-medium improvements at post-intervention for disability (PROMIS physical function domain). There was no significant difference between the groups (between group effect size [Cohens' d] = 0.28 [95% CI -0.02 to 0.57]).	Some concerns
	MBSR *vs* Usual Care	Donnino et al., 2021	Disability (RDQ) scores decreased more from pre- to post-intervention in the MBSR group than the usual care group (within group mean difference [95% CI]: MBSR = -2.5 [-7.6, -1.4]; usual care = 1.1 [-2.3, 4.5]).	High
	MBSR *vs* Psychophysiologic Symptom Relief Therapy	Donnino et al., 2021	Mean disability (RDQ) scores for Psychophysiologic Symptom Relief Therapy lower than those for MBSR at post-intervention (effect size [Cohens' d] = -0.96; $p < 0.01$)	High
	MBSR + Exercise Program + Usual Care *vs* Usual Care Alone	Zou et al., 2024	MBSR + Exercise Program + Usual Care was more effective at reducing disability (Bath Ankylosing Spondylitis – Functional Index) post-intervention than usual care alone ($p < 0.05$).	High
Abbreviations: ASES = Arthritis Self-Efficacy Scale; BDI = Beck Depression Inventory; BPI = Brief Pain Inventory; CBT = Cognitive Behavioral Therapy; CES-D = Center for Epidemiological Studies Depression Inventory; CPAQ-8 = Chronic Pain Acceptance Questionnaire 8; CPSES = Chronic Pain Self-Efficacy Scale; DASS-21 = Depression Anxiety and Stress Scale-21; EMM = Estimated Marginal Mean; F = F-statistic from an analysis of variance (ANOVA); GAD-2 = Generalized Anxiety Disorder-2; HADS = Hospital Anxiety and Depression Scale; HAQ-DI = Health Assessment Questionnaire – Disability Index; M = Mean; MBCT = Mindfulness-Based Cognitive Therapy; MBSR = Mindfulness-Based Stress Reduction; NRS = Numerical Rating Scale; ns = not significant; ODI = Oswestry Disability Index; PASS = Pain Anxiety Symptom Scale; PCS = Pain Catastrophizing Scale; PHQ-8 = Patient Health Questionnaire-8; PHQ-9 = Patient Health Questionnaire-9; PROMIS = Patient-Reported Outcomes Measurement Information System; PSEQ = Pain Self-Efficacy Questionnaire; PSWQ = Penn State Worry Questionnaire; RDQ = Roland-Morris Disability Questionnaire; ROB = Risk of bias; RR = Relative Risk; SE = Standard Error; SF-36 = 36-Item Short Form Survey; SF-MPQ = Short-Form McGill Pain Questionnaire; t = t-statistic from a paired-samples t-test; tDCS = Transcranial direct-current stimulation; TSK = Tampa Scale of Kinesiophobia; VAS = Visual Analogue Scale; WOMAC = Western Ontario and McMaster Universities Osteoarthritis Index.				
