# Supplementary material for: The Effects of Mindfulness‐Based Programs on Pain‐Related Cognitions in Individuals With Chronic Musculoskeletal Pain: A Systematic Review and Meta‐Analysis
Source: Pain Res Manag. 2026 Nov 9;2026:6495015. doi: 10.1155/prm/6495015 (PMC13746321; doi:10.1155/prm/6495015)
Supplement: Supplementary file 1 [file PRM-2026-6495015-s001.zip › Supplemental Digital Content 7_MIND SR_v5_22022026.pdf]

Supplemental Digital Content 7: Sensitivity Analyses

Outcomes	Analysis	Number of Participants (Studies)	Pooled Impact of Mindfulness-Based Programs
**Pain Catastrophizing** (Mindfulness-Based Programs vs No Intervention)	Primary analysis, all studies comparing mindfulness-based programs to no intervention	744 (10 studies)	SMD 1.00<br>95% CI 0.56 to 1.45<br>I^2 = 86.7%
	Sensitivity analysis, removing studies with imputed SDs	515 (9 studies)	SMD 1.11<br>95% CI 0.57 to 1.65<br>I^2 = 87.4%
	Sensitivity analysis, removing outlier studies	644 (7 studies)	SMD 0.38<br>95% CI 0.15 to 0.60<br>I^2 = 48.0%
**Pain Catastrophizing** (Mindfulness-Based Programs vs Education)	Primary analysis, all studies comparing mindfulness-based programs to education	719 (4 studies)	SMD 0.67<br>95% CI -0.05 to 1.39<br>I^2 = 95.2%
	Sensitivity analysis, removing outlier studies	538 (3 studies)	SMD 0.28<br>95% CI 0.10 to 0.47<br>I^2 = 13.0%
**Chronic Pain Acceptance** (Mindfulness-Based Programs vs No Intervention)	Primary analysis, all studies comparing mindfulness-based programs to no intervention, assuming r = 0.7	400 (5 studies)	SMD 1.12<br>95% CI 0.31 to 1.92<br>I^2 = 90.8%
	Sensitivity analysis, assuming r = 0.9	400 (5 studies)	SMD 1.11<br>95% CI 0.29 to 1.93<br>I^2 = 91.1%
	Sensitivity analysis, removing outlier studies	368 4 studies	SMD 0.69<br>95% CI 0.08 to 1.31<br>I^2 = 83.5%
**Depression** (Mindfulness-Based Programs vs No Intervention)	Primary analysis, all studies comparing mindfulness-based programs to no intervention	1277 (18 studies)	SMD 0.94<br>95%CI 0.50 to 1.38<br>I^2 = 92.5%
	Sensitivity analysis, removing studies with imputed SDs	1048 (17 studies)	SMD 0.99<br>95%CI 0.50 to 1.48<br>I^2 = 92.5%
	Sensitivity analysis, removing outlier studies	811 (12 studies)	SMD 0.47<br>95% CI 0.30 to 0.64<br>I^2 = 28.2%
**Anxiety** (Mindfulness-Based Programs vs No Intervention)	Primary analysis, all studies comparing mindfulness-based programs to no intervention, assuming r = 0.7	946 (12 studies)	SMD 1.08<br>95% CI 0.57 to 1.59<br>I^2 = 92.1%
	Sensitivity analysis, assuming r = 0.9	946 (12 studies)	SMD 1.08<br>95% CI 0.56 to 1.59<br>I^2 = 92.2%
	Sensitivity analysis, removing outlier studies	439 (7 studies)	SMD 0.59<br>95% CI 0.30 to 0.88<br>I^2 = 52.1%
**Pain Intensity** (Mindfulness-Based Programs vs No Intervention)	Primary analysis, all studies comparing mindfulness-based programs to no intervention	1090 (15 studies)	SMD 0.80<br>95% CI 0.38 to 1.23<br>I^2 = 90.1%
	Sensitivity analysis, removing studies with imputed SDs	826 (13 studies)	SMD 0.81<br>95% CI 0.28 to 1.34

			I^2 = 91.5%
	Sensitivity analysis, removing outlier studies	927<br>(13 studies)	SMD 0.49<br>95% CI 0.24 to 0.73<br>I^2 = 63.8%
**Pain Intensity** (Mindfulness-Based Programs vs Cognitive Behavioral Therapy)	Primary analysis, all studies comparing mindfulness-based programs to cognitive behavioral therapy, assuming r = 0.7	1365<br>(5 studies)	SMD -0.19<br>95% CI -0.80 to 0.43<br>I^2 = 95.9%
	Sensitivity analysis, assuming r = 0.5	1365<br>(5 studies)	SMD -0.19<br>95% CI -0.80 to 0.43<br>I^2 = 95.9%
	Sensitivity analysis, assuming r = 0.9	1365<br>(5 studies)	SMD -0.19<br>95% CI -0.80 to 0.43<br>I^2 = 95.9%
	Sensitivity analysis, removing outlier studies	1198<br>(4 studies)	SMD 0.13<br>95% CI -0.03 to 0.30<br>I^2 = 36.1%
**Pain Intensity** (Mindfulness-Based Programs vs Education)	Primary analysis, all studies comparing mindfulness-based programs to education	715<br>(4 studies)	SMD 0.74<br>95% CI -0.16 to 1.64<br>I^2 = 96.8%
	Sensitivity analysis, removing outlier studies	534<br>(3 studies)	SMD 0.28<br>95% CI -0.11 to 0.67<br>I^2 = 78.4%
**Disability** (Mindfulness-Based Programs vs No Intervention)	Primary analysis, all studies comparing mindfulness-based programs to no intervention	815<br>(11 studies)	SMD 0.48<br>95% CI 0.04 to 0.92<br>I^2 = 88.2%
	Sensitivity analysis, removing studies with imputed SDs	586<br>(10 studies)	SMD 0.54<br>95% CI 0.01 to 1.07<br>I^2 = 89.3%
	Sensitivity analysis, removing outlier studies	604<br>(8 studies)	SMD 0.21<br>95% CI 0.04 to 0.38<br>I^2 = 5.4%
