# Supplementary material for: The Effects of Mindfulness‐Based Programs on Pain‐Related Cognitions in Individuals With Chronic Musculoskeletal Pain: A Systematic Review and Meta‐Analysis
Source: Pain Res Manag. 2026 Nov 9;2026:6495015. doi: 10.1155/prm/6495015 (PMC13746321; doi:10.1155/prm/6495015)
Supplement: Supplementary file 1 [file PRM-2026-6495015-s001.zip › Supplemental Digital Content 8_MIND SR_v5_22022026.pdf]

Supplemental Digital Content 8: Funnel Plots of Standard Error by Hedges' g

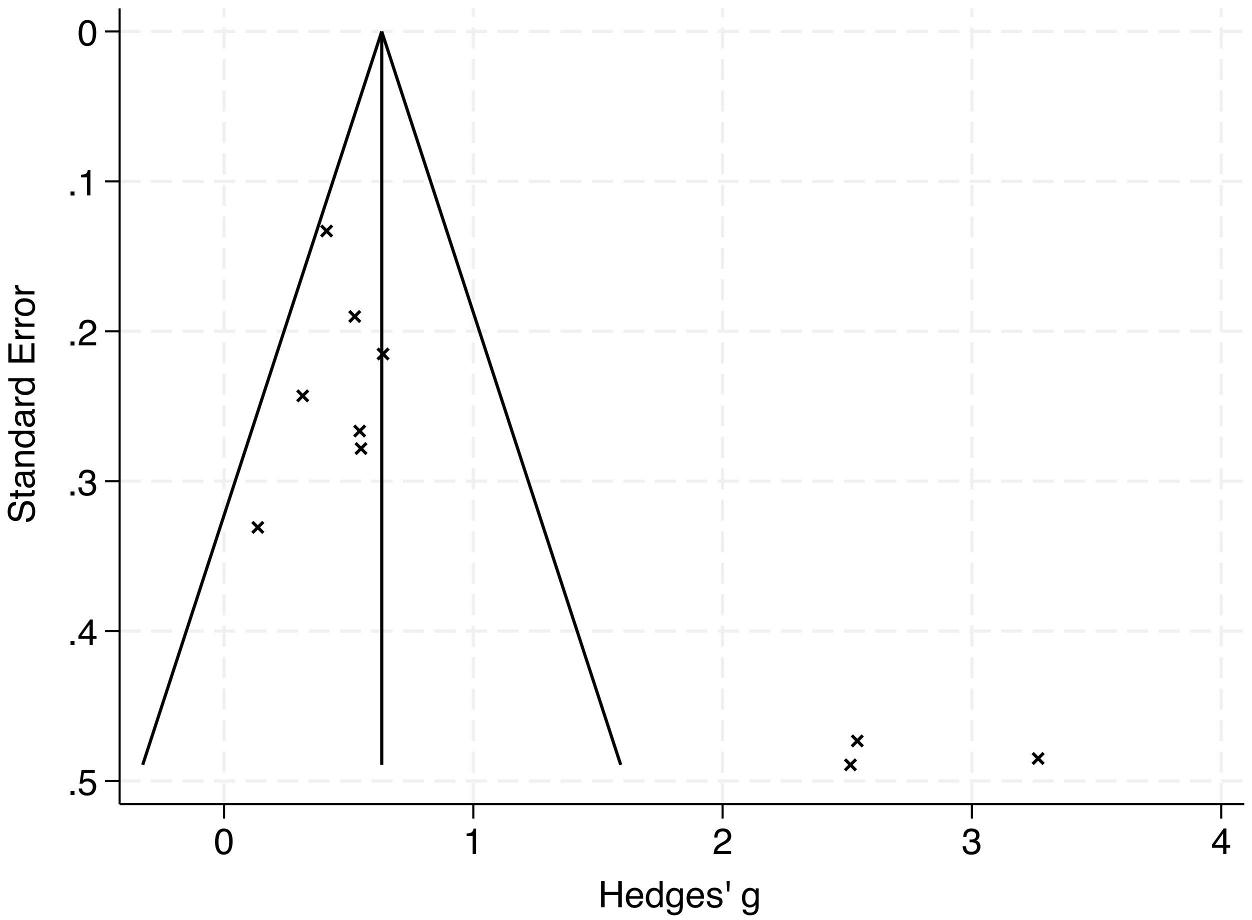

A: Funnel plot of standard error by Hedges' g for trials included in the meta-analysis for the effect of mindfulness-based programs on pain catastrophizing compared with no intervention immediately post-intervention.

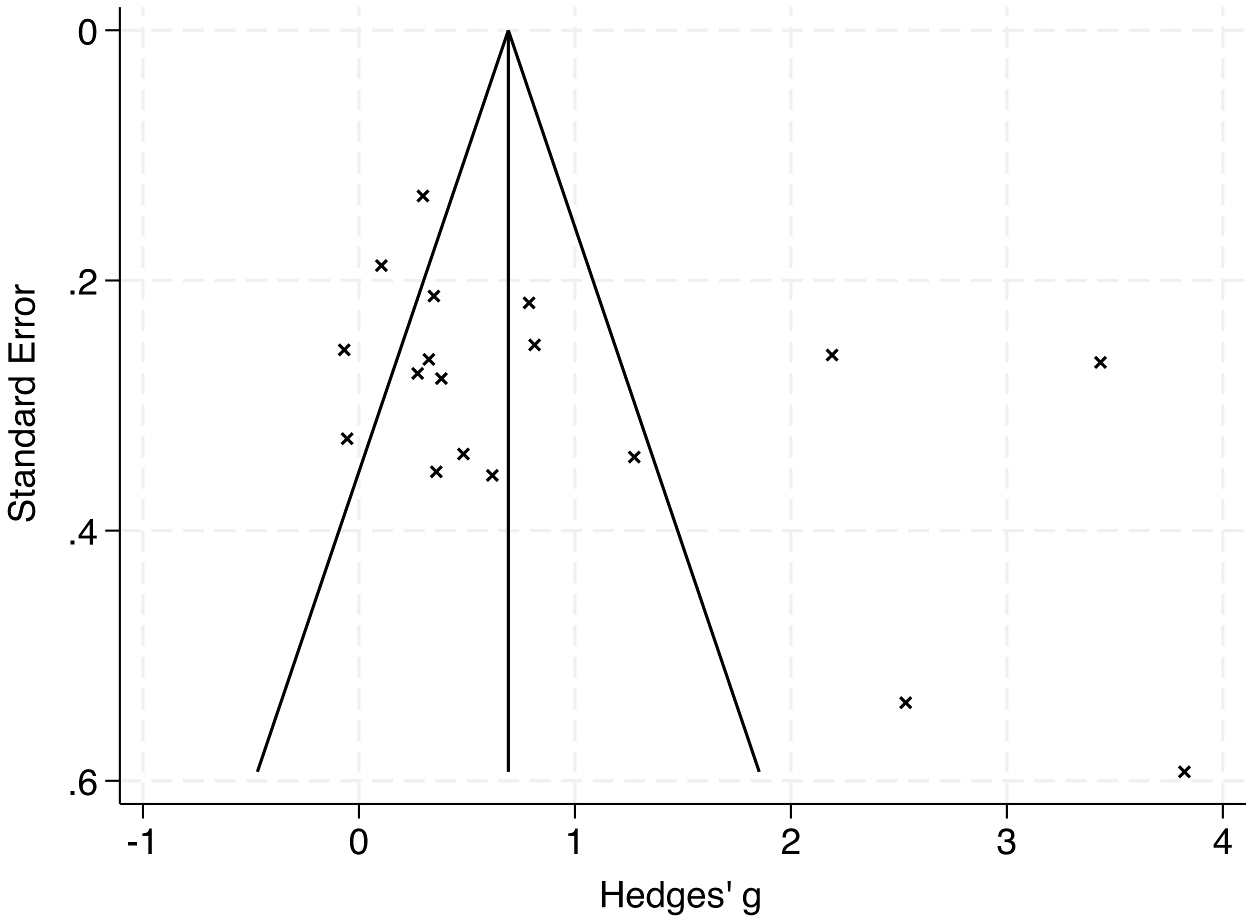

B: Funnel plot of standard error by Hedges’ g for trials included in the meta-analysis for the effect of mindfulness-based programs on depression compared with no intervention immediately post-intervention.

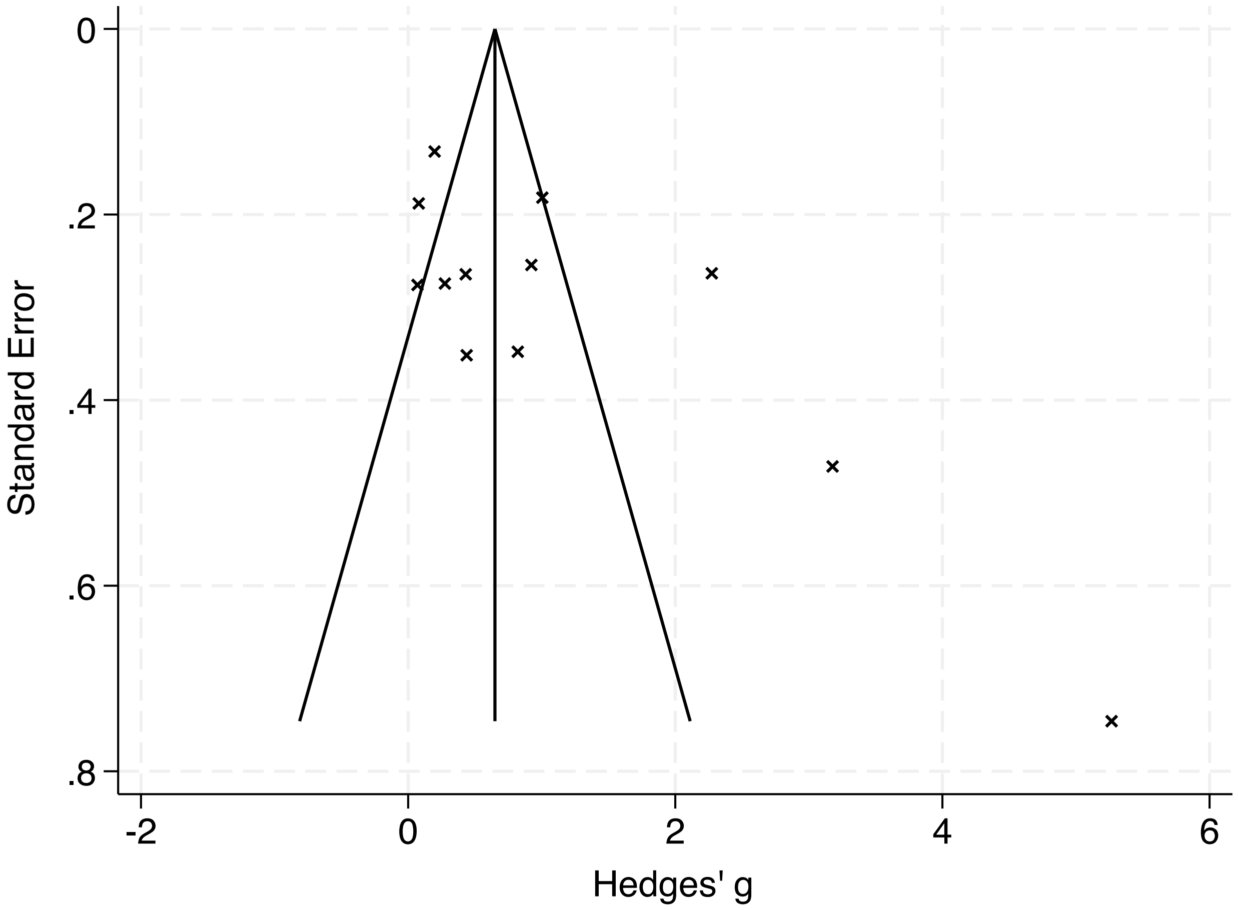

C: Funnel plot of standard error by Hedges' g for trials included in the meta-analysis for the effect of mindfulness-based programs on anxiety compared with no intervention immediately post-intervention.

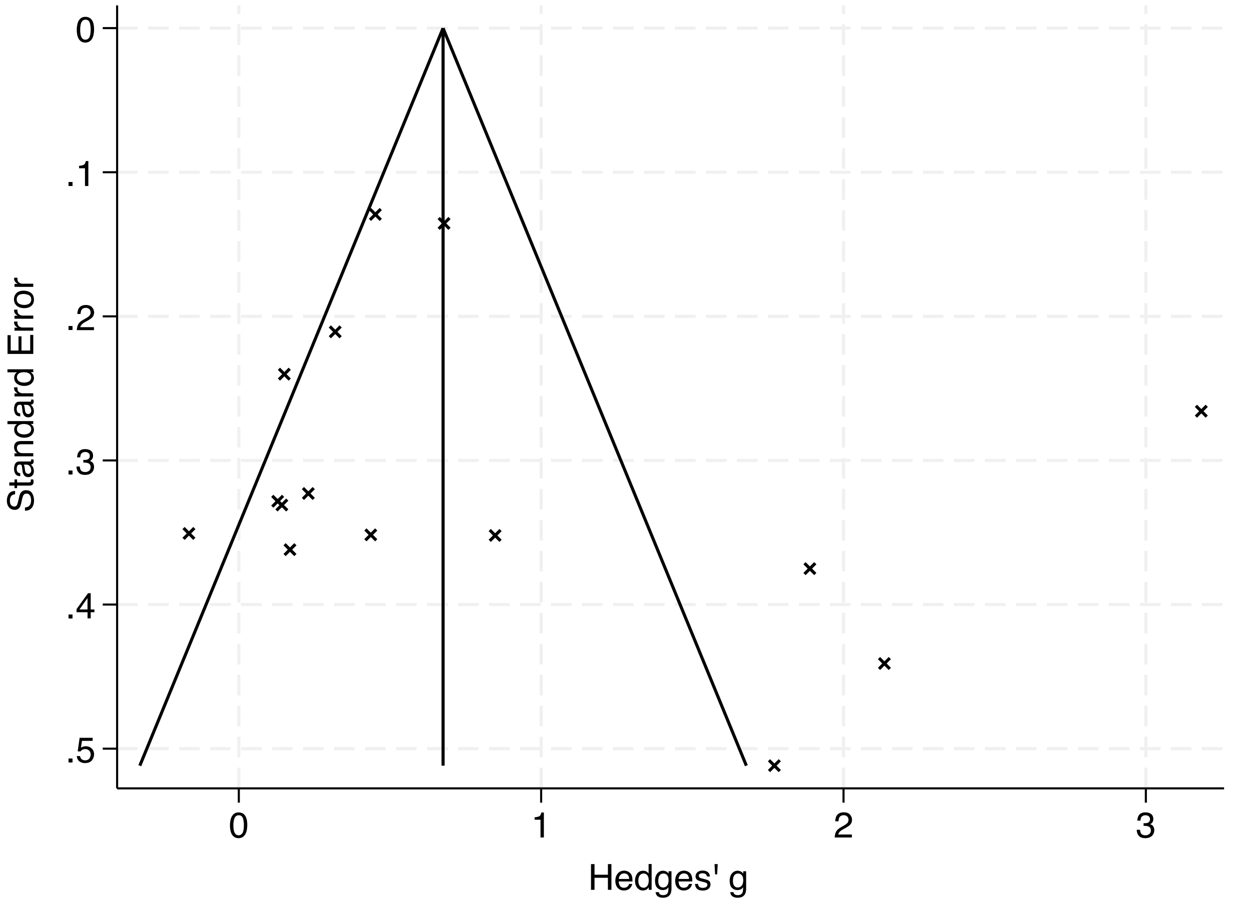

D: Funnel plot of standard error by Hedges' g for trials included in the meta-analysis for the effect of mindfulness-based programs on pain intensity compared with no intervention immediately post-intervention.

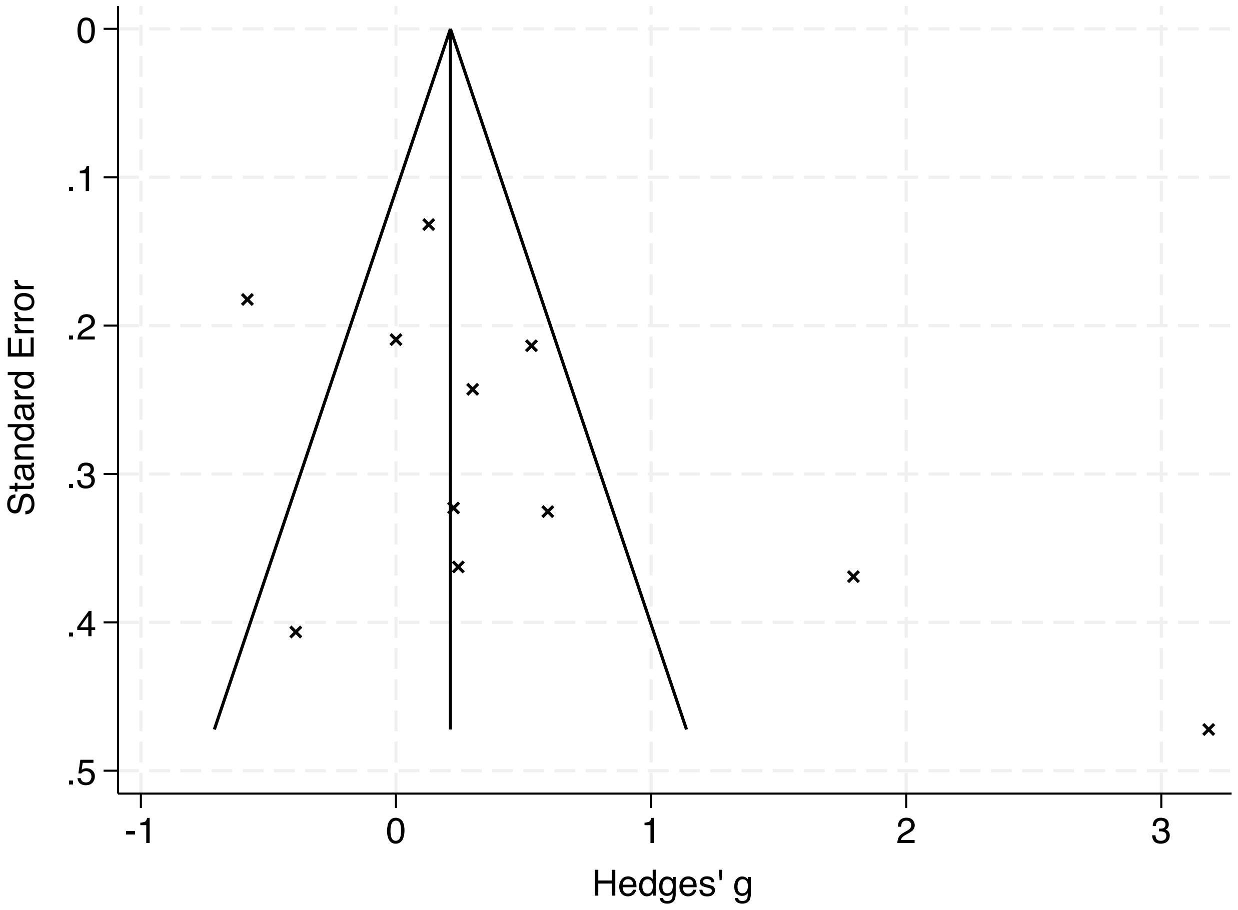

E: Funnel plot of standard error by Hedges' g for trials included in the meta-analysis for the effect of mindfulness-based programs on disability compared with no intervention immediately post-intervention.
