# Supplementary material for: The Effects of Mindfulness‐Based Programs on Pain‐Related Cognitions in Individuals With Chronic Musculoskeletal Pain: A Systematic Review and Meta‐Analysis
Source: Pain Res Manag. 2026 Nov 9;2026:6495015. doi: 10.1155/prm/6495015 (PMC13746321; doi:10.1155/prm/6495015)
Supplement: Supplementary file 1 [file PRM-2026-6495015-s001.zip › Supplemental Digital Content 9_MIND SR_v5_22022026.pdf]

Supplemental Digital Content 9: Summary of Findings

**Mindfulness-based programs for people with chronic musculoskeletal pain**							
**Patient or population:** People with chronic musculoskeletal pain<br>**Intervention:** Mindfulness-based programs<br>**Comparison:** No restrictions placed on type of comparator<br>**Timepoint:** Immediately post-intervention							
**Outcomes**	**Number of Participants (Studies)**	**SMD**	**95% CI**	**I^2 (%)**	**Egger's p-value (two-tailed)**	**Certainty of Evidence (GRADE)**	**Comments**
**Pain Catastrophizing** Mindfulness vs No Intervention	744 (10 studies)	1.00	0.56 to 1.45	86.7	0.012	⊕⊖⊖⊖ Very Low Due to risk of bias, inconsistency, and publication bias	See Appendix 7 for sensitivity analyses
**Pain Catastrophizing** Mindfulness vs Education	719 (4 studies)	0.67	-0.05 to 1.39	95.2	–	⊕⊕⊖⊖ Low Due to risk of bias and inconsistency	See Appendix 7 for sensitivity analyses
**Chronic Pain Acceptance** Mindfulness vs No Intervention	400 (5 studies)	1.12	0.31 to 1.92	90.8	–	⊕⊖⊖⊖ Very Low Due to risk of bias, inconsistency, and publication bias	See Appendix 7 for sensitivity analyses
**Depression** Mindfulness vs No Intervention	1277 (18 studies)	0.94	0.50 to 1.38	92.5	0.088	⊕⊖⊖⊖ Very Low Due to risk of bias, inconsistency, and publication bias	See Appendix 7 for sensitivity analyses
**Anxiety** Mindfulness vs No Intervention	946 (12 studies)	1.08	0.57 to 1.59	92.1	0.025	⊕⊖⊖⊖ Very Low Due to risk of bias, inconsistency, and publication bias	See Appendix 7 for sensitivity analysis
**Psychological Distress** Mindfulness vs No Intervention	261 (4 studies)	0.75	0.12 to 1.38	81.5	–	⊕⊖⊖⊖ Very Low Due to risk of bias, inconsistency,	–

						and imprecision	
**Pain Intensity** Mindfulness vs No Intervention	1090 (15 studies)	0.80	0.38 to 1.23	90.1	0.464	⊕⊕⊖⊖ Low Due to risk of bias and inconsistency	See Appendix 7 for sensitivity analyses
**Pain Intensity** Mindfulness vs Cognitive Behavioral Therapy	1365 (5 studies)	-0.19	-0.80 to 0.43	95.9	–	⊕⊕⊖⊖ Low Due to risk of bias and inconsistency	See Appendix 7 for sensitivity analyses
**Pain Intensity** Mindfulness vs Education	715 (4 studies)	0.74	-0.16 to 1.64	96.8	–	⊕⊕⊖⊖ Low Due to risk of bias and inconsistency	See Appendix 7 for sensitivity analyses
**Disability** Mindfulness vs No Intervention	815 (11 studies)	0.48	0.04 to 0.92	88.2	0.081	⊕⊕⊖⊖ Very Low Due to risk of bias and inconsistency, and publication bias	See Appendix 7 for sensitivity analyses
**SMD** = standardised mean difference; **95% CI** = 95% confidence interval							
**GRADE Working Group grades of evidence**<br>**High quality:** Further research is very unlikely to change our confidence in the estimate of effect.<br>**Moderate quality:** Further research is likely to have an important impact on our confidence in the estimate of effect and may change the estimate.<br>**Low quality:** Further research is very likely to have an important impact on our confidence in the estimate of effect and is likely to change the estimate.<br>**Very low quality:** We are very uncertain about the estimate.							
